# Biotin‐ and Chloroalkane‐Modified Nucleotides for Dual Display of Proteins on DNA Through Streptavidin and Halotag Binding

**DOI:** 10.1002/anie.6259985

**Published:** 2026-08-19

**Authors:** Giammarco Liuni, Lenka Poštová Slavětínská, Miroslav Hájek, Veronika Raindlová, Michal Hocek

**Affiliations:** ^1^ Institute of Organic Chemistry and Biochemistry Czech Academy of Sciences Prague Czech Republic; ^2^ Department of Organic Chemistry Faculty of Science Charles University Prague Czech Republic

**Keywords:** affinity tags, DNA polymerases, DNA‐protein conjugates, nucleotides, protein display

## Abstract

We designed and synthesized 2’‐deoxyribonucleoside triphosphates (dNTPs) bearing chlorohexyl and biotin modifications attached to position 7 of 7‐deazaadenine or to position 5 of cytosine. The modified dNTPs served as substrates for KOD XL DNA polymerase in the enzymatic construction of DNA bearing either one or a combination of the two modifications. The chlorohexyl‐linked DNA underwent covalent reactions with halotag protein, while the biotin‐linked DNA formed stable non‐covalent complexes with monomeric streptavidin. The dually modified DNA bearing both modifications at different distances apart from each other were used for sequence‐specific, spatially defined display of both halotag and streptavidin proteins. The study provides the foundation for display of proteins on DNA rulers.

## Graphical abstract

Modified 2’‐deoxy‐7‐deazaadenosine bearing a chlorohexyl moiety and 2’‐deoxycytidine nucleotides bearing biotin were used in the enzymatic synthesis of dually modified dsDNAs that served as scaffolds for display of halotag and monomeric streptavidin proteins.

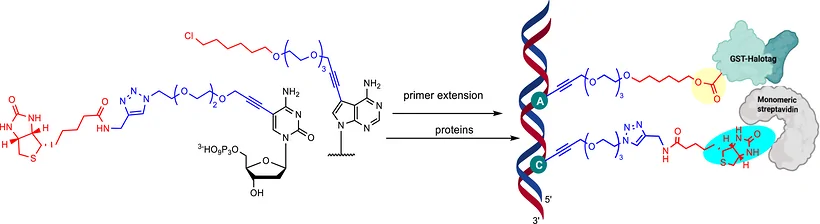

## 1 Introduction

The intrinsic properties of DNA based on its well‐defined structure, sequence‐based programmability, straightforward synthetic accessibility, high stability, and structural flexibility, make it a highly attractive and promising scaffold in the self‐assembly of nanomaterials [1]. The DNA duplex can be engineered to enable the spatially defined arrangement of diverse functional moieties along its sequence, effectively serving as a well‐defined ruler for the precise positioning of small molecules, or (bio)macromolecules [2, 3]. Such functionalized constructs can be obtained either through the hybridization of two modified oligonucleotides (ONs) or by two or more hybridizing short modified ONs or peptide‐nucleic acids (PNA) oligomers, to a complementary single‐stranded template [4]. These approaches have been successfully applied to the display of (oligo)saccharides [5, 6, 7] and peptides [8, 9], enabling multivalent interactions that have been exploited for protein targeting [10]. Furthermore, DNA‐templated protein assemblies have been achieved via hybridization of chemically synthesized protein‐oligonucleotide conjugates [11, 12], including DNA origami displaying arrays of enzymes [13, 14, 15, 16, 17, 18]. In most of these applications, the protein‐binding or ‐recruiting modifications were chemically linked to either the 3’‐ or the 5’‐end of the oligonucleotide sequence, which are easy to synthesize but significantly limit the potential arrangement of the modifications in the assembled constructs. Sequence‐specific internal modification of oligonucleotides or dsDNA with proteins or protein‐binding modifications would be highly desirable but not yet reported.

Enzymatic synthesis of base‐modified DNA is an established approach [19, 20, 21] using diverse 5‐substituted pyrimidine and/or 7‐substituted 7‐deazapurine 2’‐deoxyribonucleoside triphosphates (dNTPs) that can be used for sequence‐specific incorporation of a modification either through the design of the sequence in primer extension (PEX) or through single nucleotide incorporation (SNI) followed by PEX with natural nucleotides [22]. We [23, 24, 25, 26] and others [27, 28] have previously reported 2’‐deoxyribonucleoside triphosphates (dNTPs) bearing reactive groups for cross‐linking with proteins after enzymatic incorporation to DNA but the cross‐linking reactions are only efficient for DNA‐binding proteins recognizing the particular DNA sequence or using a protein‐binding aptamer [28]. For a general display of proteins, the DNA should recruit and bind the proteins solely based on the affinity to the modification.

To that end, we selected biotin as a well‐known ligand to streptavidin (and its monomeric form mSA) [29, 30] and haloalkane modification, which is known to form a covalent ester bond with halotag protein [31]. Several biotin‐linked dNTPs (mostly to position 5 of uracil) are commercially available and are often used for pull down of the DNA [32, 33, 34]. Haloalkane‐ linked dNTPs were not reported to the best of our knowledge, although chemically synthesized 3’‐haloalkane‐linked ONs were used for origami display of enzymes through halotag ligation [15]. Hence, the aim of this work was the design and synthesis of dNTPs bearing biotin and haloalkane linked through different tethers and test them in enzymatic synthesis of dually modified DNA for site‐specific display of two different proteins (mSA and halotag).

## 2 Results and Discussion

We designed and synthesized a modified 2’‐deoxy‐7‐deazaadenosine nucleotide **dA^pegHalo^TP** in which a chlorohexyl group as a halotag ligand was attached at position 7 via triethyleneglycol (PEG3) linker, which worked very well in the attachment of other reactive groups [35]. For attachment of biotin, we chose 2’‐deoxycytidine, and we linked the biotin at position 5 either via a short amidopropargyl linker (**dC^Bio^TP**) or a longer PEG3 linker (**dC^pegBio^TP**) (Scheme 1) to investigate the influence of the length of the linker on the complex formation with mSA. The synthesis of **dA^pegHalo^TP** (41% yield) was achieved by single‐step Sonogashira cross‐coupling reaction on 7‐iodo‐2’‐deoxy‐7‐deazaadenosine 5’‐*O*‐triphosphate (**dA^I^TP**) [36] with terminal alkyne (**2**), which was easily prepared according to published procedures [37, 38], starting from the PEG3 moiety, and subsequent reaction of propargyl alcohol **1** with 1‐chloro‐6‐iodohexane (Scheme 1A). The synthesis of biotin modified nucleotides started with the preparation of propargyl biotin building block (**4**), via succinimidyl ester intermediate (**3**) [39]. We chose amino‐modified **dC^NH2^TP** [40, 41, 42] and azido‐modified **dC^peg3N3^TP** (Scheme S4 in Supporting Information) [43], as intermediate nucleotides for further functionalization. The reaction of NHS‐ester **3** with **dC^NH2^TP** afforded the amide‐linked modified nucleotide **dC^Bio^TP** in 43% yield (Scheme 1B). The PEG‐triazine‐linked **dC^pegBio^TP** (60% yield) was obtained through a copper‐catalyzed alkyne‐azide cycloaddition (CuAAC) [44] reaction of terminal alkyne **4** with **dC^peg3N3^TP** (Scheme 1B). The final modified nucleotides were purified by HPLC and obtained in sufficient quantities for the enzymatic syntheses of DNA.

**SCHEME 1:**
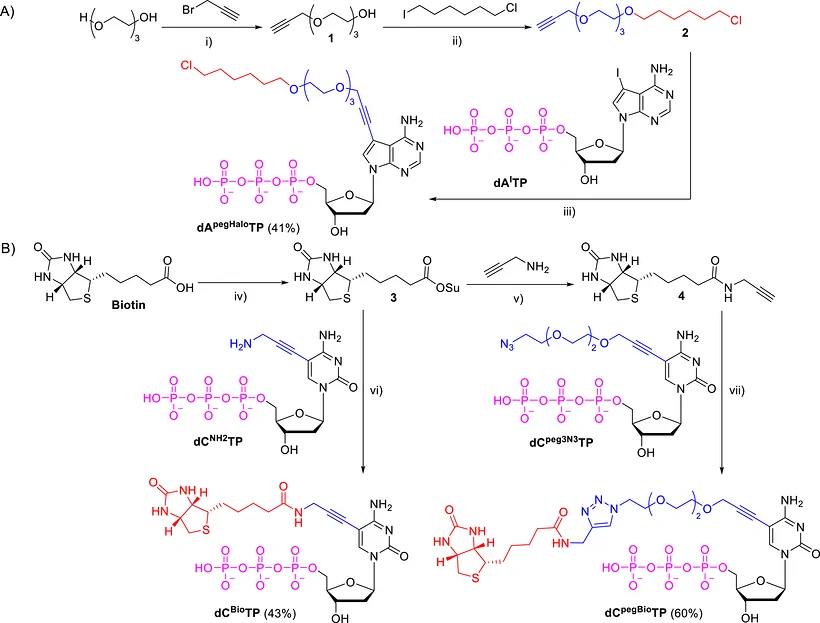
(A) Synthesis of halotag modified nucleotide **dA^pegHalo^TP**. (B) Synthesis of biotin modified nucleotides **dC^Bio^TP** and **dC^pegBio^TP**. Reaction conditions: (i) *t*BuOK, THF, 0°C to 25°C, 17h; (ii) NaH, THF/DMF (1:1), 0°C to 25°C, 17h; (iii) Pd(OAc)_2_, CuI, TPPTS, Et_3_N, H_2_O/DMF (2:1), 45’, 75°C; (iv) NHS, EDC^.^HCl, DMF, 25°C, 24h; (v) Et_3_N, DMF, 25°C, 24h; (vi) TEAB 1M, 55°C, 3h; (vii) Sodium ascorbate, CuSO_4_^.^5H_2_O, TBTA, H_2_O/DMSO (1:1), 25°C, 5h. For more details see Supporting Information, pages S4–S11.

The modified nucleotides **dA^pegHalo^TP**, **dC^Bio^TP** and **dC^pegBio^TP** were subsequently tested as substrates for KOD XL DNA polymerase in primer extension (PEX) experiments, first with short template encoding just for one modification (Figure 1A). All of them were efficiently incorporated, yielding full‐length 19‐mer PEX products (**19DNA_A^pegHalo^**, **19DNA_C^X^**; X = Bio, pegBio), as confirmed by denaturing PAGE analysis (Figure 1B–D; for the ON sequences see Tables 1 and S1) and MALDI‐TOF MS. Next, we explored the feasibility of synthetizing longer PEX products (30‐, 40‐, and 50‐mers) containing two copies of the modified nucleotide, introduced at defined positions along the oligonucleotides strand, with increasing distance between them (10, 20 and 30 base pairs; for the ON sequences see Tables 1 and S1). For that purpose, we tested only **dA^pegHalo^TP**, and the full‐length double‐modified PEX products (**nDNA_A^pegHalo^**; n = 30, 40, 50) were successfully obtained (Figure S3). All doubly modified ONs were also characterized by MALDI‐TOF analysis (Table S3, Figures S29–S31).

**FIGURE 1:**
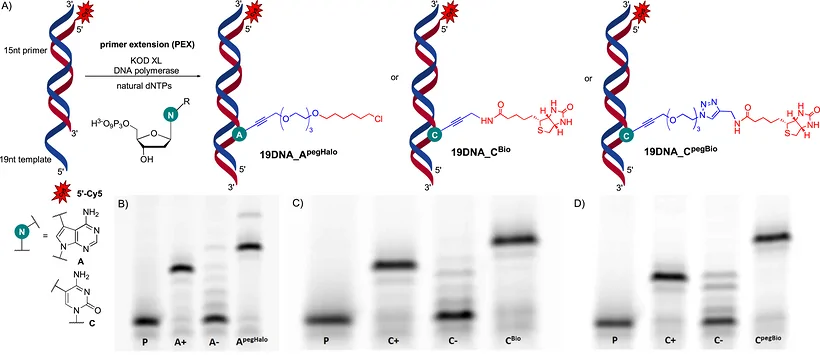
(A) Scheme of enzymatic synthesis of **19DNA_A^pegHalo^**, **19DNA_C^Bio^**, and **19DNA_C^pegBio^**. Denaturing PAGE analyses of PEX reactions using KOD XL DNA polymerase, (P) primer **Prim248short** 5’‐(Cy5)‐labeled with (B) **dA^pegHalo^TP** and template **Oligo 1A**: (A+): dATP, dGTP; (A‐): dGTP; (A^pegHalo^): **dA^pegHalo^TP**, dGTP. (C, D) **dC^X^TP** (X = Bio, pegBio) and template **Oligo 1C**: (C+): dCTP, dGTP; (C‐): dGTP; (C^Bio^): **dC^Bio^TP**, dGTP; (C^pegBio^): **dC^pegBio^TP**, dGTP. For more details see Supporting Information pages S16 and S17.

**TABLE 1:** List of selected sequences of primers and templates used in this study.

ON Name	Length (nt)	Sequence (5’→ 3’)^a^
**Prim248short** ^b^	15	CATGGGCGGCATGGG
**Oligo 1A**	19	CCCTCCCATGCCGCCCATG
**Oligo 1C**	19	CCCGCCCATGCCGCCCATG
**Oligo 2A_10**	30	CCCTCGACGACGACTCCCATGCCGCCCATG
**T30‐10**	30	CCCGACCACCACCCTCCCATGCCGCCCATG
**Oligo 2A_20**	40	CCCTACGACGACGACGACGACGACTCCCATGCCGCCCATG
**T40‐20**	40	CCCGACCACCACCACCACCACCACTCCCATGCCGCCCATG
**Oligo 2A_30**	50	CCCTGACGACGACGACGACGACGACGACGACGACTCCCATGCCGCCCATG
**T50‐30**	50	CCCGCACCACCACCACCACCACCACCACCACCACTCCCATGCCGCCCATG
**T60‐40**	60	CCCGACCACCACCACACCACCACCACCACCACCACCACCACCACTCCCATGCCGCCCATG
**T70‐50**	70	CCCGACACCACCACACCACCACCACACCACCACCACCACCACCACCACCACCACTCCCATGCCGCCCATG

^a^ In the template ONs the segments forming duplex with the primer are underlined.

^b^ 5’‐(Cy5)‐ or 5’‐(6‐FAM)‐labeled.

Then, we designed other five ON sequences of varying lengths 30‐ to 70‐mer (see Tables 1 and S1) for the simultaneous incorporation of **dA^pegHalo^TP**, in combination with either **dC^Bio^TP** or **dC^pegBio^TP** (Figure 2A). In this design, the two modifications were placed at specific positions within the DNA sequence, with increasing distance between them (10, 20, 30, 40, and 50 base pairs). The synthesis of the full‐length double‐modified DNAs (**nDNA_A^pegHalo^C^X^**; n = 30, 40, 50, 60, 70; X = Bio, pegBio) by PEX was successfully achieved for both combinations of modified nucleotides and across all oligonucleotide lengths tested (Figure 2B). All these dually modified oligonucleotides were characterized by MALDI‐TOF or LC‐ESI‐MS analysis (Table S3 and Figures S32–S41).

**FIGURE 2:**
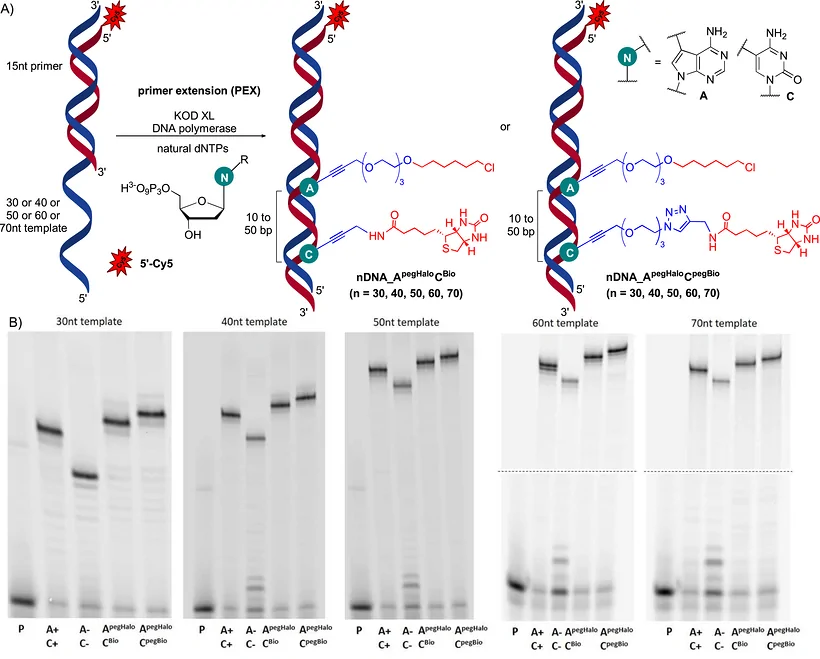
(A) Enzymatic synthesis of **nDNA_A^pegHalo^C^X^** (n = 30, 40, 50, 60, 70; X = Bio, pegBio). (B) Denaturing PAGE analyses of PEX reactions with combination of **dA^pegHalo^TP** and **dC^X^TP** (X = Bio, pegBio) using KOD XL DNA polymerase: (P) primer **Prim248short** 5’‐(Cy5)‐labeled; templates **T30‐10**/**T40‐20**/**T50‐30**/**T60‐40**/**T70‐50**: (A+, C+): dATP, dGTP, dCTP, dTTP; (A‐, C‐): dGTP, dTTP; (A^pegHalo^, C^Bio^): **dA^pegHalo^TP**, **dC^Bio^TP**, dGTP, dTTP; (A^pegHalo^, C^pegBio^): **dA^pegHalo^TP**, **dC^pegBio^TP**, dGTP, dTTP (for the uncropped gels of the 60nt and the 70nt templates, see Figure S4). For more details see Supporting Information pages S18 and S19.

Next, we evaluated the reactivity of halotag‐modified (**19DNA_A^pegHalo^**) and biotin‐modified (**19DNA_C^pegBio^**) double‐stranded 19‐mer DNAs toward recombinant GST‐tagged halotag protein (Figure 3A) and non‐covalent complex formation with recombinant monomeric streptavidin protein (mSA) (Figure 3B), respectively. The reaction between **19DNA_A^pegHalo^** and GST‐Halotag was conducted using two equivalents of protein, at 37°C for 17 h. Formation of the covalent conjugate (**19DNA_A^pegHalo_Halotag^**) was clearly observed by denaturing SDS‐PAGE analysis (lane 4 in Figure 3C). The natural DNA (**19DNA_A^Natural^**) did not show any reactivity or complex formation with the protein, clearly showing that there are no non‐specific interactions involved (lane 2 in Figure 3C). Simple kinetic studies of this reaction indicated that comparable levels of DNA‐protein covalent conjugate formation are achieved within 5–17 h (38%–39% conversions, Figure S8). The kinetic studies revealed substantially reduced reactivity, both in terms of conjugation efficiency and reaction rate, compared with the reported conjugation of free halotag ligands to halotag protein [31]. Nevertheless, similarly reduced conjugation efficiencies have been observed for halotag functionalization of DNA origami nanostructures, even when larger molar excesses of halotag protein were employed [15]. The isoelectric point of halotag protein (pI ∼ 5.0) [45] indicates that the protein is predominantly negatively charged under the reaction conditions. Consequently, electrostatic repulsion between the negatively charged oligonucleotide and the protein surface, particularly in the vicinity of the ligand‐binding site, may hinder productive conjugate formation, thereby reducing both the rate and overall efficiency of the conjugation reaction [46]. Subsequently, the interaction between **19DNA_C^pegBio^** and monomeric streptavidin was examined under analogous conditions (two equivalents of protein, at 37°C for 17 h). Complex formation (**19DNA_C^pegBio_mSA^**) was confirmed by protein electrophoresis using native PAGE (lane 4 in Figure 3D), and no interaction between natural DNA (**19DNA_C^Natural^**) and the protein was detected (lane 2 in Figure 3D). Likewise, the complex formation was observed for the short‐linker biotin analogue **19DNA_C^Bio^** (**19DNA_C^Bio_mSA^**), detected on protein native PAGE, albeit with a lower spot intensity (Figure S7A). The reduced reactivity of **19DNA_C^Bio^** toward mSA, compared to **19DNA_C^pegBio^**, could be attributed to the shorter linker length, which likely increased steric hindrance due to the closer proximity between the DNA and the protein, thereby limiting efficient biotin‐streptavidin interaction. In contrast, the longer PEG3 linker in **19DNA_C^pegBio^** apparently provided greater flexibility and thus better accessibility of the biotin moiety. A similar trend in reactivity was observed by performing kinetic studies of these reactions: **19DNA_C^Bio^** exhibited consistently lower reactivity across all time points investigated (15%–22% conversions, Figure S9), compared to **19DNA_C^pegBio^** (69%–88% conversions, Figure S9). To further characterize the interaction between the biotinylated DNA constructs and mSA protein, the affinity of 5’‐(Cy5)‐labeled **19DNA_C^pegBio^** was determined using fluorescent nickel‐plate binding affinity assay (FNBA), yielding a dissociation constant (K_D_) of 27 nM (Figure S24). Although this value is higher than the reported affinity of free biotin toward mSA [30], it remains within the nanomolar range, indicating a strong binding. The same analysis could not be performed for **19DNA_C^Bio^**, as its interaction with mSA could not be reliably quantified under the assay conditions, consistent with the weaker binding observed by protein native PAGE. Based on its superior interaction with mSA, **dC^pegBio^TP** was evaluated for compatibility with the TUNEL assay [47] in HeLa cells (Figure S25A–D). Following incorporation of **dC^pegBio^TP** by Terminal Deoxynucleotidyl Transferase (TdT) at 3’‐OH ends generated by DNA strand breaks, labelling was detected through binding of fluorescent mSA iFluor 647 protein, resulting in 18% iFluor 647‐positive singlets (Figure S25D), whereas negative controls yielded only 0.29% and 0% iFluor 647‐positive singlets (Figure S25B,C), as determined by flow cytometry. Based on these experiments, only the **dC^pegBio^TP** containing the longer triethyleneglycol linker was used in further studies.

**FIGURE 3:**
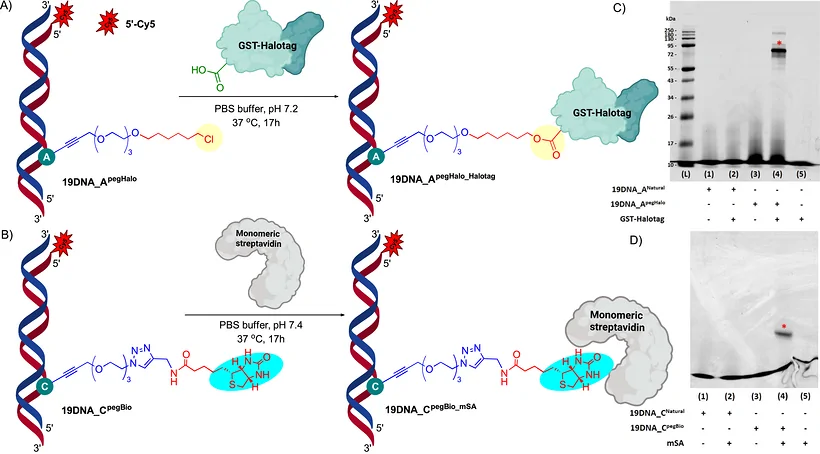
(A) Cross‐linking of **19DNA_A^pegHalo^** with GST‐Halotag protein. (B) Reaction of **19DNA_C^pegBio^** with mSA protein. (C) 10% SDS‐PAGE analysis of 5’‐(Cy5)‐labeled **19DNA_A^X^** (X = Natural, pegHalo) with GST‐Halotag protein (37°C, 17 h). Cy5 channel scan. (L) ladder, (*) DNA‐protein conjugate. (D) 10% protein native PAGE analysis of 5’‐(Cy5)‐labeled **19DNA_C^X^** (X = Natural, pegBio) with mSA protein (37°C, 17 h). Cy5 channel scan. (*) DNA‐protein complex. For more details see Supporting Information pages S23–24.

The next step was to assess the feasibility of simultaneous binding of two molecules of the target protein to the doubly modified DNA bearing two copies of the modified nucleotide. For this purpose, we performed a preliminary investigation with 30‐, 40‐, and 50‐mer double‐stranded DNAs, containing two chlorohexyl modifications at different distances (**30DNA_A^pegHalo^**, **40DNA_A^pegHalo^**, **50DNA_A^pegHalo^**), and tested their reactivity toward GST‐Halotag protein. SDS‐PAGE analysis revealed the formation of the double DNA‐protein conjugate as a minor product (10%–17% conversion), alongside the mono‐conjugation product as the major one (62%–74% conversion), for every tested DNA (Figure S10). We then moved to the main goal of this study, to evaluate the possibility of constructing a spatially defined and sequence‐specific assembly of two different proteins on a DNA scaffold. We first examined the reactivity of dually modified dsDNAs, bearing both chlorohexyl and biotin functionalities (**nDNA_A^pegHalo^C^pegBio^**; n = 30, 40, 50, 60, 70), toward GST‐Halotag or mSA proteins individually (Figure 4A). The first screening was performed on **nDNA_A^pegHalo^C^X^** (n = 30, 40, 50; X = Bio, pegBio), using varying amounts of GST‐halotag (1, 10, and 50 equivalents) or mSA (1, 10, 100, and 500 equivalents). The highest conversions of the covalent DNA‐halotag conjugate formation (**nDNA_A^pegHalo_Halotag^C^X^**; n = 30, 40, 50; X = Bio, pegBio) were obtained for the highest tested amount (50 equivalents; Figures S11–S13). In the case of the DNA‐mSA complexes formation (**nDNA_A^pegHalo^C^X_mSA^**; n = 30, 40, 50; X = Bio, pegBio), no significant difference in the conversions was observed between 100 and 500 equivalents (Figures S16–S18), and hence the lower amount (100 equivalents) was chosen for the continuation of the study. The reactions between double‐modified DNAs (**nDNA_A^pegHalo^C^pegBio^**; n = 30, 40, 50, 60, 70) and GST‐halotag protein (50 equivalents) were carried out at 37°C for 17 h. SDS‐PAGE analysis clearly demonstrated the formation of the covalent conjugates **nDNA_A^pegHalo_Halotag^C^pegBio^** (n = 30, 40, 50, 60, 70) for all tested oligonucleotides, with conversions in the range 35%–67% (lanes 2, 4, 6, 8, 10 in Figure 4B). No covalent conjugate formation was observed with corresponding natural DNAs (lanes 1, 3, 5, 7, 9 in Figure 4B). Gel analysis of the covalent binding of GST‐halotag to **nDNA_A^pegHalo^C^Bio^** (n = 30, 40, 50, 60, 70; short‐linker biotin moiety) showed a reactivity comparable to the one observed for **nDNA_A^pegHalo^C^pegBio^** (n = 30, 40, 50, 60, 70), under the same experimental conditions, with the exception of **50DNA_A^pegHalo^C^Bio^** which exhibited a markedly lower spot intensity (Figure S14A,B). The non‐covalent interactions between **nDNA_A^pegHalo^C^pegBio^** (n = 30, 40, 50, 60, 70) and mSA protein (100 equivalents) were studied at 37°C for 17 h. The complex formation (**nDNA_A^pegHalo^C^pegBio_mSA^**; n = 30, 40, 50, 60, 70) was clearly observed by protein native PAGE, with 55%–64% conversions (lanes 2, 4, 6, 8, 10 in Figure 4C), and compared to the reactivity of natural DNAs toward mSA, showing that no non‐specific interactions occurred (lanes 1, 3, 5, 7, 9 in Figure 4C). The interaction between **nDNA_A^pegHalo^C^Bio^** (n = 30, 40, 50, 60, 70) and monomeric streptavidin was found to be very weak, if not negligible, particularly in the case of **50DNA_A^pegHalo^C^Bio^** (Figure S19A,B). The reduced reactivity of the short‐linker biotin moiety, also in longer DNA constructs, is consistent with the trend observed for shorter oligonucleotides (Figure S7A), thereby supporting the previously proposed steric hindrance hypothesis.

**FIGURE 4:**
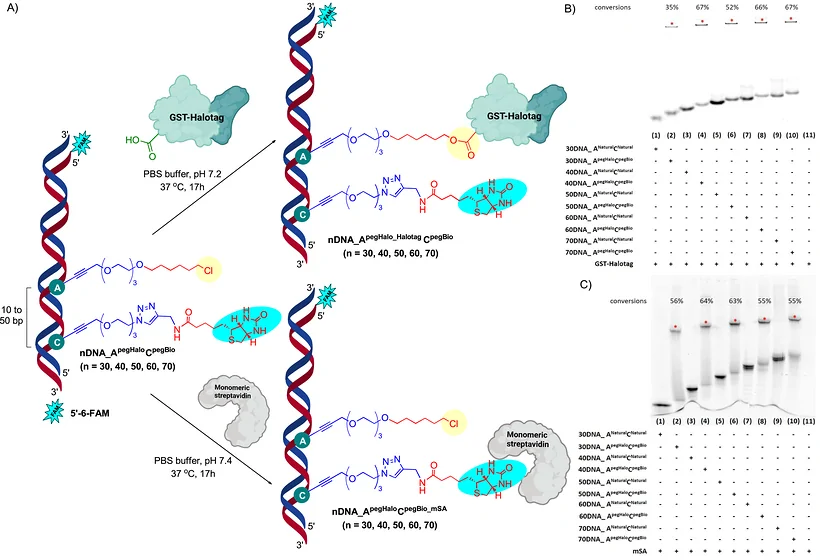
(A) Reaction of **nDNA_A^pegHalo^C^pegBio^** (n = 30, 40, 50, 60, 70) with GST‐Halotag or mSA proteins. (B) 12% SDS‐PAGE analysis of 5’‐(6‐FAM)‐labeled **nDNA_A^X^C^Y^** (n = 30, 40, 50, 60, 70; X = Natural, pegHalo; Y = Natural, pegBio) with GST‐Halotag. FAM channel scan. (*) DNA‐protein conjugate. Calculated conversions of conjugation reactions. For gel image including the protein ladder see Figure S15 in SI. (C) 12% protein native PAGE analysis of 5’‐(6‐FAM)‐labeled **nDNA_A^X^C^Y^** (n = 30, 40, 50, 60, 70; X = Natural, pegHalo; Y = Natural, pegBio) with mSA. FAM channel scan. (*) DNA‐protein complex. Calculated conversions of complexes formation. For more details see SI pages S27‐S35.

Using the previously optimized experimental conditions, we then investigated the simultaneous binding of both proteins to dually modified **nDNA_A^pegHalo^C^pegBio^** (n = 30, 40, 50, 60, 70). The reactions were performed by one‐pot addition of GST‐halotag (50 equivalents) and mSA (100 equivalents) proteins, and incubated at 37°C for 17 h (Figure 5A). All results of these reactions were analyzed only by protein native PAGE, as the non‐covalent biotin‐streptavidin interaction would not be preserved under denaturing SDS‐PAGE conditions. For each oligonucleotide, appropriate control samples were included (DNA only, DNA plus GST‐Halotag, DNA plus mSA). Gel analysis confirmed the formation of ternary DNA‐protein complexes (**nDNA_A^pegHalo_Halotag^C^pegBio_mSA^**; n = 30, 40, 50, 60, 70). The distinct electrophoretic mobility of the double‐bound DNA‐protein products observed in the gels (lane 4 in each gel in Figure 5B), compared to the controls, confirmed the successful formation of the assemblies. Putative structures of representative examples of the dually modified protein‐DNA constructs for **nDNA_A^pegHalo_Halotag^C^pegBio_mSA^** (n = 30, 70) are shown in Figure 5C to provide a visual representation of the expected complexes. The quantification of the conversions (within the range 41%–50%, lane 4 of each gel in Figure S21B) was performed using gels scanned after a shorter time (1 h) of the electrophoretic separations, as lower density (7%) gels were employed (compared to the previously used 10% or 12%) to improve spot resolution and enhance the mobility of DNA‐protein complexes. Under these conditions, spots corresponding to unreacted DNA were no longer visible in the final scans (4 h) of the electrophoretic separations, precluding accurate quantification at later time points. The overall efficiency of the double display of two proteins (conversions 41%–50%) is comparable or even slightly higher than combination of the individual events (conversions 52%–67% each). The resulting dually modified protein‐DNA constructs (**nDNA_A^pegHalo_Halotag^C^pegBio_mSA^**; n = 30, 40, 50, 60, 70) display the two proteins separated by 10, 20, 30, 40 and 50 nucleotides, which corresponds to the distances of 3.4, 6.8, 10.2, 13.6 and 17 nm apart from each other. The conversions in formation of all these constructs did not differ significantly, indicating that even the 10 nucleotides (one turn of the dsDNA helix) could be sufficient for accommodating two proteins if the linker is sufficiently long. On the other hand, no ternary DNA‐proteins complexes were observed for the modified DNAs with short‐linker biotin **nDNA_A^pegHalo^C^Bio^** regardless of the two modifications (Figure S21A).

**FIGURE 5:**
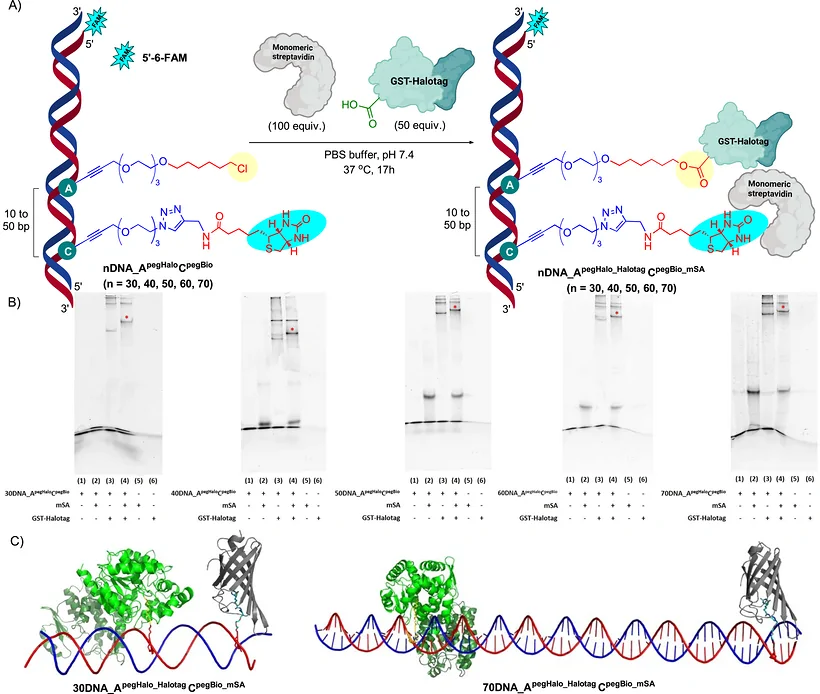
(A) Reaction of **nDNA_A^pegHalo^C^pegBio^** (n = 30, 40, 50, 60, 70) with both GST‐Halotag and monomeric streptavidin proteins at the same time. (B) 7% protein native PAGE analysis of 5’‐(6‐FAM)‐labeled **nDNA_A^pegHalo^C^pegBio^** (n = 30, 40, 50, 60, 70) with either mSA (lanes 2), GST‐Halotag (lanes 3), or combination of mSA and GST‐Halotag (lanes 4). FAM channel scan. (*) Ternary DNA‐Halotag‐mSA complex. For the uncropped gels see Figure S21A. For more details see Supporting Information pages S35–S37. (C) Visualization of putative structures of DNA‐Halotag‐mSA constructs for **nDNA_A^pegHalo_Halotag^C^pegBio_mSA^** (n = 30, 70) based on AlphaFold 3 and molecular modeling.

The versatility of the developed methodology and its applicability to random sequences was further demonstrated by site‐specific incorporation of the two modified nucleotides. As the previously reported SNI followed by PEX was applicable only for one modified nucleotide [22], we complemented it with TdT‐mediated 3‘‐end labelling to introduce the second modification. Thus the SNI with **dA^pegHalo^TP**, followed by PEX with natural dNTPs and TdT mediated incorporation of **dC^pegBio^TP** (Scheme S5) yielded the desired dually modified single strand DNA (ssDNA) **32ON_A^pegHalo^C^pegBio^** (for the ON sequences see Table S1; for denaturing PAGE analysis see Figure S5; for mass characterization, see Table S3 and Figures S42–S44). This dually modified oligonucleotide successfully reacted with the corresponding protein partners (GST‐halotag and mSA iFluor 555 proteins, see Scheme S6) to form the desired DNA‐protein assembly (for protein native PAGE see Figure S22). It should be noted that despite recent efforts [48, 49], a robust and general methodology for enzymatic site‐specific incorporations of multiple modified nucleotides into DNA or RNA is an unsolved problem and challenge for future development.

## 3 Conclusion

In conclusion, we designed and synthesized chlorohexyl‐ and biotin‐modified dNTPs (**dA^pegHalo^TP**, **dC^Bio^TP** and **dC^pegBio^TP**), and proved their efficient incorporation by KOD XL DNA polymerase in primer extension reactions. This enabled the preparation of sequence‐defined, dually modified DNA scaffolds (**nDNA_A^pegHalo^C^X^**; n = 30, 40, 50, 60, 70; X = Bio, pegBio), with defined positioning of the modifications within the DNA sequences. Both modifications retained selective specificity toward their respective protein partners leading to the formation of covalent DNA‐Halotag conjugate (**19DNA_A^pegHalo_Halotag^**) and non‐covalent DNA‐monomeric streptavidin complexes (**19DNA_C^Bio_mSA^** and **19DNA_C^pegBio_mSA^**), in the case of singly modified DNAs, as well as dually modified ones (**nDNA_A^pegHalo_Halotag^C^pegBio^**; **nDNA_A^pegHalo^C^pegBio_mSA^**). Notably, the simultaneous display of both proteins to the same DNA strand (**nDNA_A^pegHalo_Halotag^C^pegBio_mSA^**; n = 30, 40, 50, 60, 70) was successfully achieved only for the double‐modified DNAs bearing the long‐linker biotin modification (**nDNA_A^pegHalo^C^pegBio^**; n = 30, 40, 50, 60, 70) in good conversions 41%–50%. The observed conversions were comparable across all constructs, regardless of the distance between the modification sites, indicating that the double binding was not strongly dependent on the distance between the functional moieties within the oligonucleotide sequence and even one turn of the helix (3.4 nm) is sufficient for arrangement and display of two proteins on DNA. No reactivity was observed for DNAs modified with the short‐linker biotin moiety (**nDNA_A^pegHalo^C^Bio^**) in the double binding with GST‐Halotag and monomeric streptavidin proteins. These results highlighted the critical role of linker length in modulating protein binding, with the PEG3 linker providing improved accessibility and reduced steric hindrance, particularly in the case of the DNA–protein ternary complex formation. Moreover, the flexibility of the synthetic approach allows the generation of tailored DNA constructs through different modification strategies, although a general methodology for site‐specific enzymatic incorporation of multiple modifications remains a challenge for future development. These findings demonstrate the potential of modified DNA as a scaffold for the spatial organization of proteins and provide a basis for the development of more complex biomolecular assemblies. This study represents the first step toward future applications where the halotag and mSA will be used as adaptors for expression of fusion constructs with functional proteins (e.g. enzymes) for the spatially defined display of arrays diverse proteins (for example cascades of enzymes) on DNA ruler in analogy to previous literature [15]. Studies along these lines are under way in our laboratory.

## Author Contributions

**Giammarco Liuni**: conceptualization, investigation, data curation, writing – original draft, writing – review and editing, methodology. **Lenka Poštová Slavětínská**: data curation, investigation, writing – review and editing. **Miroslav Hájek**: data curation, investigation. **Veronika Raindlová**: data curation, supervision, writing – review and editing, writing – original draft. **Michal Hocek**: conceptualization, methodology, supervision, funding acquisition, writing – original draft, writing – review and editing, project administration.

## Conflicts of Interest

The authors declare no conflicts of interest.

## Supporting information



The authors have cited additional references within the Supporting Information [50, 51, 52]. Raw data are reposited and available at: https://doi.org/10.48700/9vzwg‐wxy73 **Supporting File**: anie74276‐sup‐0001‐SuppMat.pdf.

## Data Availability

The data that support the findings of this study are openly available in Narodni repozitar at https://data.narodni‐repozitar.cz/, reference number https://doi.org/10.48700/9vzwg‐wxy73.
