# Supplementary material for: Biotin‐ and Chloroalkane‐Modified Nucleotides for Dual Display of Proteins on DNA Through Streptavidin and Halotag Binding
Source: Angew Chem Int Ed Engl. 2026 Aug 19;65(42):e6259985. doi: 10.1002/anie.6259985 (PMC13653569; doi:10.1002/anie.6259985)
Supplement: Supplementary file 1 [file ANIE-65-e6259985-s001.pdf]

Table of contents

1. Experimental section – Chemistry S3

General remarks S3

1.1. Synthesis of modified nucleotide **dA^{pegHalo}TP** S4

1.2. Synthesis of propargyl biotin S6

1.3. Synthesis of modified nucleotide **dC^{Bio}TP** S7

1.4. Synthesis of modified nucleotide **dC^{pegBio}TP** S8

2. Experimental section – Biochemistry S12

General remarks S12

Modified nucleotides used in the study S13

2.1. List of sequences of primers, templates and oligonucleotides used in the study S14

2.2. Primer Extension (PEX) – Single incorporation of one modified **dN^{X}TP** S16

2.2.1. Single incorporation of **dA^{pegHalo}TP** using 19-mer template – Analytical scale S16

2.2.2. Single incorporation of **dC^{X}TP** using 19-mer template – Analytical scale S17

2.3. Primer Extension (PEX) – Multiple incorporation of modified **dN^{X}TPs** S17

2.3.1. Multiple incorporation of **dA^{pegHalo}TP** using 30-mer, 40-mer and 50-mer templates – Analytical scale S17

2.3.2. Multiple incorporation of combination of **dA^{pegHalo}TP** and **dC^{X}TP** using 30-mer, 40-mer, 50-mer, 60-mer and 70-mer templates – Analytical scale S18

2.4. Single nucleotide incorporation (SNI) of **dA^{pegHalo}TP** and subsequent dCTP, and primer extension (PEX) using 31-mer template, followed by **dC^{pegBio}TP** incorporation mediated by Terminal Deoxynucleotidyl Transferase (TdT) S19

2.5. Procedure for semipreparative scale synthesis of dsDNA S21

2.6. Single nucleotide incorporation (SNI) of **dA^{pegHalo}TP** and subsequent dCTP using 31-mer template – Semipreparative scale S21

2.7. Preparation of ONs for MALDI-TOF analysis S21

2.7.1. Single incorporation of **dA^{pegHalo}TP** using 19-mer template – Semipreparative scale S21

2.7.2. Single incorporation of **dC^{X}TP** using 19-mer template – Semipreparative scale S21

2.7.3. Multiple incorporation of **dA^{pegHalo}TP** using 30-mer, 40-mer and 50-mer templates – Semipreparative scale S22

2.8. Procedures for ssON generation S22

2.8.1. ssON generation via magnetoseparation S22

2.8.2. ssON generation via λ-exonuclease digestion S22

2.9. Electrophoretic mobility shift assay (EMSA) S23

2.9.1. EMSA of natural and modified dsDNA (19-mer) with recombinant GST-Halotag protein S23

2.9.2. EMSA of natural and modified dsDNA (19-mer) with recombinant mSA protein S24

2.9.3. EMSA of modified dsDNA (19-mer) with recombinant GST-Halotag protein – time dependent experiment S25
2.9.4. EMSA of modified dsDNA (19-mer) with recombinant mSA protein – time dependent experiment S26
2.9.5. EMSA of natural and double modified dsDNA (30-, 40- and 50-mer) with recombinant GST-Halotag protein S26
2.9.6. EMSA of double modified dsDNA (30-, 40- and 50-mer) with recombinant GST-Halotag protein – concentration dependent experiment S27
2.9.7. EMSA of natural and double modified dsDNA (30-, 40-, 50-, 60- and 70-mer) with recombinant GST-Halotag protein – optimized reaction conditions S29
2.9.8. EMSA of double modified dsDNA (30-, 40- and 50-mer) with recombinant mSA protein – concentration dependent experiment S31
2.9.9. EMSA of natural and double modified dsDNA (30-, 40-, 50-, 60- and 70-mer) with recombinant mSA protein – optimized reaction conditions S33
2.9.10. EMSA of double modified dsDNA (30-, 40-, 50-, 60- and 70-mer) with combination of recombinant mSA protein and recombinant GST-Halotag protein S35
2.9.11. EMSA of double modified ssDNA (32-mer) with combination of recombinant mSA iFluor 555 protein and recombinant GST-Halotag protein S38
2.10. Representative examples of DNA-Halotag-mSA assemblies S39
2.11. Fluorescent Nickel-plate binding affinity assay (FNBA) S39
2.12. Modified TUNEL assay using Terminal Deoxynucleotidyl Transferase (TdT) mediated incorporation of **dC^{pegBio}TP** and flow cytometric detection with recombinant mSA iFluor 647 protein S40
3. Copies of MALDI-TOF and LC-MS mass spectra S44
4. Copies of NMR spectra S56
5. References S63

1. Experimental section – Chemistry

General remarks

All reagents and solvents were purchased from the commercial suppliers and used as received. Phosphoryl chloride ($POCl_3$) and trimethyl phosphate [$PO(OMe)_3$] were distilled prior to use. Dried solvents were purchased from Acros Organics. The water used in the synthetic part was of HPLC quality. Unless stated otherwise, all reactions were performed in heatgun-dried glassware under argon atmosphere, using standard septa techniques. The reactions were monitored by thin-layer chromatography (TLC) on silica gel 60 F_{254} plates (Merck) and visualized by UV (254 nm), or alternatively by solution of 0.75% [w/v] potassium permanganate and 5% [w/v] potassium carbonate in water with 10% of sodium hydroxide. Triphosphorylation reactions were monitored by TLC using IPAV (*i*PrOH/H_2O/NH_4OH, ratio 11:2:7) as mobile phase. Purifications were performed by manual column chromatography using silica gel (40–63 µm). Purifications of nucleoside triphosphates were performed by HPLC (Waters modular HPLC system), using column Phenomenex Kinetex EVO C18 (Kinetex® 5 µm EVO C18 100 Å, AXIA Packed LC Column 250 × 21.2 mm). NMR spectra were measured on Bruker AVANCE III HD 400 MHz spectrometer (400.1 MHz for ^{1}H and 100.6 MHz for ^{13}C), Bruker AVANCE III HD 500 MHz spectrometer (500.0 MHz for ^{1}H, 125.7 MHz for ^{13}C and 202.4 MHz for ^{31}P), Bruker AVANCE III HD 600 MHz spectrometer (600.1 MHz for ^{1}H and 150.9 MHz for ^{13}C), in $CDCl_3$, DMSO-d_6, CD_3OD (referenced to the residual solvent signal) or D_2O (reference to *t*BuOH as the internal standard – 1.25 ppm for ^{1}H, 31.6 ppm for ^{13}C) at 25 °C. Chemical shifts are given in ppm (δ-scale), coupling constants (J) in Hz with the following splitting abbreviations: s = singlet, d = doublet, t = triplet, q = quartet, m = multiplet. Complete assignment of all NMR signals was performed using a combination of H,H-COSY, H,C-HSQC and H,C-HMBC experiments. Mass spectra and high-resolution mass spectra were measured on a LTQ Orbitrap XL spectrometer (Thermo Fisher Scientific). All mass spectra were acquired by the MS service at IOCB. Purity of all final compounds was determined by NMR spectra.

Chemicals were of analytical grade. 5-iodo-2'-deoxycytidine (**dC$^{\mathbf{I}}$**) was purchased from Fluorochem Ltd. Synthesis and characterization data for 7-iodo-2'-deoxy-7-deazaadenosine 5'-*O*-triphosphate (**dA$^{\mathbf{I}}$TP**)[1] were reported previously.

1.1. Synthesis of modified nucleotide dA^{pegHalo}TP

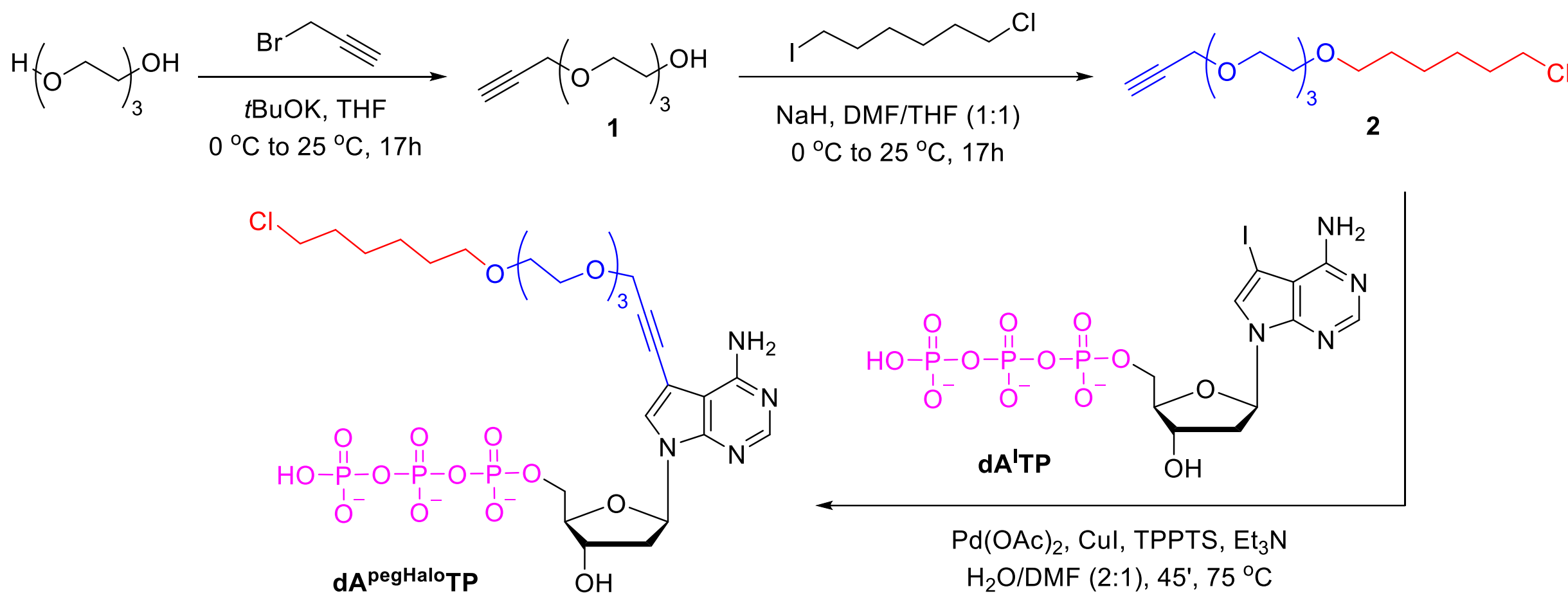

Scheme S1. Chemical synthesis of modified nucleotide **dA^{pegHalo}TP**.

2-{2-[2-(Prop-2-yn-1-yloxy)ethoxy]ethoxy}ethan-1-ol (1)[2]

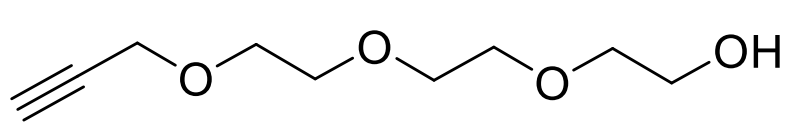

Triethylene glycol (4.5 mL, 33 mmol) was added dropwise into a suspension of *t*BuOK (1.88 g, 16.7 mmol) in THF (75 mL) at 0 °C. The mixture was stirred at 25 °C for 30 min, followed by dropwise addition of propargyl bromide (80% wt in toluene; 1.8 mL, 16.7 mmol) in THF (10 mL). The resulting mixture was stirred at 25 °C for 16 h. Then, the mixture was diluted with EtOAc and filtered through Celite. The filtrate was concentrated under reduced pressure. Purification by column chromatography (EtOAc) gave **1** (2.27 g, 12.06 mmol, 72%) as a pale-yellow liquid.

R_f = 0.32 (SiO_2, EtOAc; $KMnO_4$ stain). Analytical data (NMR, MS) matched those previously reported in literature.[2]

[2-(2-{2-[(6-Chlorohexyl)oxy]ethoxy}ethoxy)ethoxy]prop-1-yne (2)

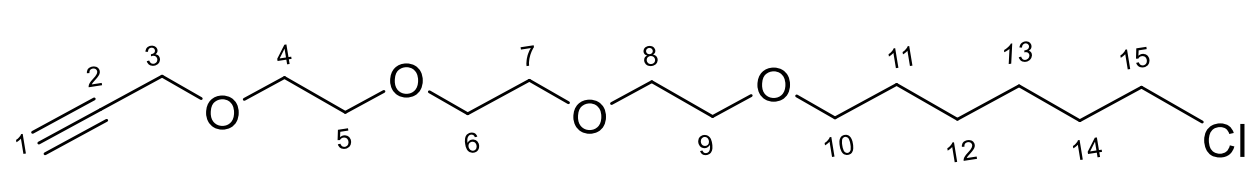

1 (1 g, 5.36 mmol) was added dropwise into a suspension of NaH (60% dispersion in mineral oil; 0.341 g, 8.53 mmol) in THF (1 M, 8.5 mL) and DMF (1 M, 8.5 mL) at 0 °C. The mixture was stirred at 0 °C for 1 h, followed by dropwise addition of 1-chloro-6-iodohexane (1.1 mL, 7.24 mmol). The resulting mixture was warmed up to 25 °C, and stirred for 16 h. Then, the mixture was treated with H_2O (20 mL), diluted with 1 M HCl (8.5 mL) and extracted with chloroform (3×50 mL). The combined organic layers were washed with brine (50 mL), dried over Na_2SO_4, and concentrated under reduced pressure. Purification by column chromatography (EtOAc) gave **2** (697 mg, 2.27 mmol, 42%) as a pale-yellow liquid.

R_f = 0.33 (SiO_2, cyclohexane/EtOAc 3:1; $KMnO_4$ stain); ^{1}H NMR (500.0 MHz, $CDCl_3$): 1.36 (m, 2H, H-12); 1.43 (m, 2H, H-13); 1.58 (m, 2H, H-11); 1.76 (m, 2H, H-14); 2.42 (t, 1H, $J_{1,3}$ = 2.4 Hz, H-1); 3.44 (t, 2H, $J_{10,11}$ = 6.6 Hz, H-10); 3.52 (t, 2H, $J_{15,14}$ = 6.7 Hz, H-15); 3.60- 3.71 (m, 12H, H-4-9); 4.19 (d, 2H, $J_{3,1}$ = 2.4 Hz, H-3); ^{13}C NMR (125.7 MHz, $CDCl_3$): 25.54 (CH_2-12); 26.81 (CH_2-13); 29.57 (CH_2-11); 32.66 (CH_2-14); 45.16 (CH_2-15); 58.51 (CH_2-3); 69.22, 70.21, 70.52, 70.70 and 70.74 (CH_2-4-9); 71.34 (CH_2-10); 74.61 (CH-1); 79.77 (C-2). ESI MS *m*/*z* (rel. %) 307 (100) $[M + H]^+$, 329 (100) $[M + Na]^+$; HR MS (ESI) for $C_{15}H_{28}O_4Cl$ $[M + H]^+$ calcd. 307.16706, found 307.16690; for $C_{15}H_{27}O_4ClNa$ $[M + Na]^+$ calcd. 329.14901. found 329.14893.

7-{3-[2-(2-{2-[(6-Chlorohexyl)oxy]ethoxy}ethoxy)ethoxy]prop-1-yn-1-yl}-2’-deoxy-7-deazaadenosine 5’-*O*-triphosphate (dA^{pegHalo}TP)

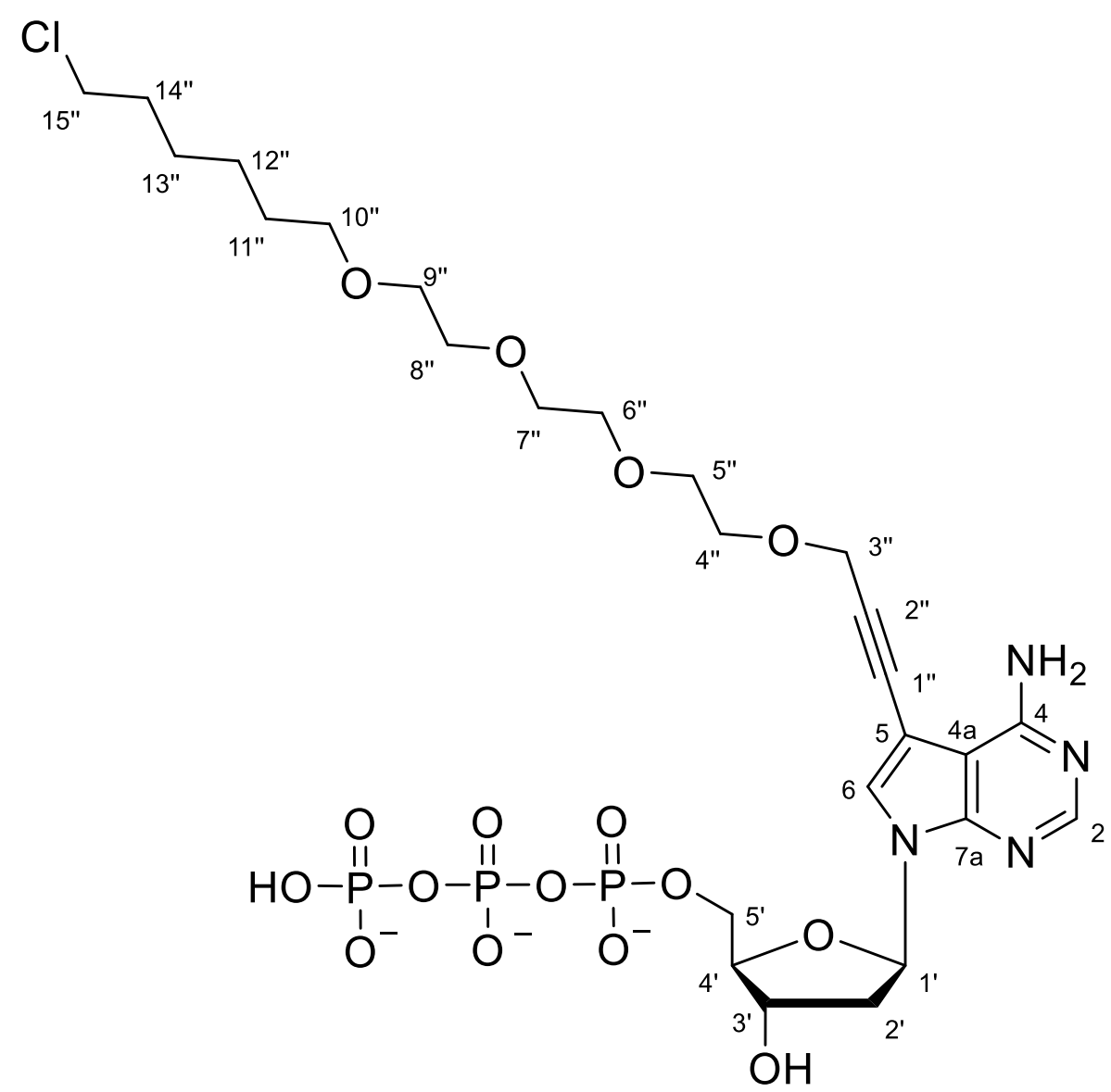

7-Iodo-2’-deoxy-7-deazaadenosine 5’-*O*-triphosphate (**dA^{I}TP**, Et_3NH^+ salt, 57.8 mg, 0.06 mmol), **2** (40 mg, 0.13 mmol), CuI (1.2 mg, 6 µmol) and Et_3N (75 µL, 0.54 mmol), in 4 mL of H_2O/DMF (2:1) were treated with a solution of $Pd(OAc)_2$ (0.8 mg, 3 µmol) and TPPTS (8.3 mg, 14 µmol) in 2 mL of H_2O/DMF (2:1). The mixture was stirred at 75 °C for 45 min. Then, it was cooled down to 25 °C and treated with a saturated solution of EDTA (2 mL), the solvents were evaporated in vacuo, and the residue was co-distilled three times with water. HPLC purification (0.1 M TEAB in H_2O to 0.1 M TEAB in H_2O/MeOH 1:1), several co-distillations with water, followed by freeze-drying from water gave **dA^{pegHalo}TP** (Et_3NH^+ salt, 27.9 mg, 0.025 mmol, 41%) as a green sticky solid.

^{1}H NMR (600.1 MHz, D_2O): 1.21 (m, 2H, H-12´´); 1.27 (m, 2H, H-13´´); 1.46 (m, 2H, H-11´´); 1.64 (m, 2H, H-14´´); 2.45 (ddd, 1H, J_{gem} = 14.0 Hz, $J_{2'a,1'}$ = 6.1 Hz, $J_{2'a,3'}$ = 3.0 Hz, H-2´a); 2.66 (bddd, 1H, J_{gem} = 14.0 Hz, $J_{2'b,1'}$ = 7.8 Hz, $J_{2'b,3'}$ = 6.3 Hz, H-2´b); 3.43 (t, 2H, $J_{10'',11''}$ = 6.7 Hz, H-10´´); 3.52 (t, 2H, $J_{15'',14''}$ = 6.7 Hz, H-15´´); 3.57 (m, 2H, H-9´´); 3.65 (m, 2H, H-8´´); 3.69 and 3.72 (2×m, 2×2H, H-6´´,7´´); 3.77 (m, 2H, H-5´´); 3.85 (m, 2H, H-4´´); 4.12 – 4.24 (m, 2H, H-5´);

4.26 (m, 1H, H-4´); 4.53 (m, 2H, H-3´´); 4.75 (m, 1H, H-3´); 6.61 (dd, 1H, $J_{1´,2´b}$ = 7.8 Hz, $J_{1´,2´a}$ = 6.1 Hz, H-1´); 7.91 (s, 1H, H-6); 8.21 (bs, 1H, H-2); ^{13}C NMR (150.9 MHz, D_2O): 26.45 (CH_2-12´´); 27.80 (CH_2-13´´); 30.33 (CH_2-11´´); 33.75 (CH_2-14´´); 41.01 (CH_2-2´); 47.61 (CH_2-15´´); 60.77 (CH_2-3´´); 67.62 (bd, $J_{C,P}$ = 4.4 Hz, CH_2-5´); 70.84 (CH_2-4´´); 71.14 (CH_2-9´´); 71.55, 71.60 and 71.64 (CH_2-5´´,6´´,7´´,8´´); 72.91 (CH_2-10´´); 73.21 (CH-3´); 80.44 (C-1´´); 85.29 (CH-1´); 87.40 (d, $J_{C,P}$ = 8.5 Hz, CH-4´); 90.43 (C-2´´); 98.49 (C-5); 104.54 (C-4a); 129.53 (CH-6); 150.01 (C-7a); 152.12 (CH-2); 157.63 (C-4); ^{31}P{^{1}H} NMR (202.4 MHz, D_2O): -22.52 (t, 1P, $J_{\beta,\alpha} = J_{\beta,\gamma}$ = 19.8 Hz, P_β); -10.62 (d, 1P, $J_{\alpha,\beta}$ = 20.0 Hz, P_α); -10.10 (d, 1P, $J_{\gamma,\beta}$ = 19.5 Hz, P_γ). ESI MS *m*/*z* (rel. %) 793 (100) [M – H]$^-$; HR MS (ESI) for $C_{26}H_{41}O_{16}N_4ClP_3$ [M – H]$^-$ calcd. 793.14244, found 793.14232.

1.2. Synthesis of propargyl biotin[3]

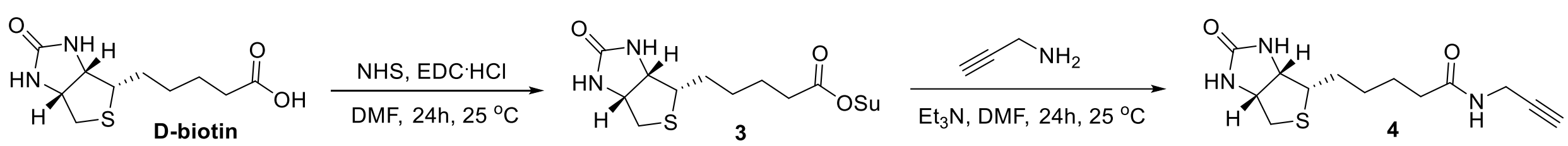

Scheme S2. Chemical synthesis of propargyl biotin **4**.

N-propargyl D-biotinamide (4)

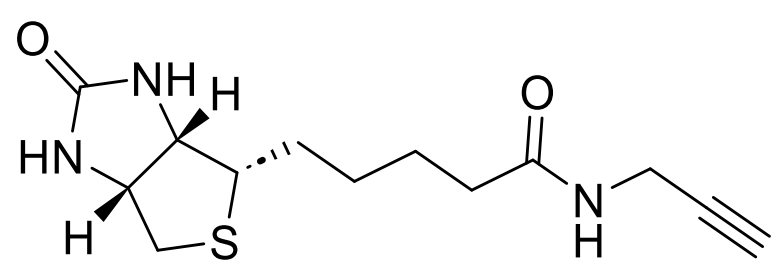

In a flask containing D-biotin (201.2 mg, 0.82 mmol) and *N*-hydroxysuccinimide (148 mg, 1.28 mmol) in DMF (12 mL), it was added 1-(3-dimethylaminopropyl)-3-ethylcarbodimide hydrochloride (195 mg, 1.01 mmol). After stirring at 25 °C for 24 h, the solvent was evaporated under reduced pressure, and the residue was washed several times by MeOH. The solvent excess was evaporated under reduced pressure to give **3** (221.1 mg, 0.65 mmol, 79%) as a white solid. The crude product was used in the following reaction without further purification.

Et_3N (70 µL, 0.5 mmol) was added to a solution of **3** (89.5 mg, 0.26 mmol) and propargyl amine (23 µL, 0.36 mmol) in DMF (5 mL). After stirring at 25 °C for 24 h, the solvent was evaporated under reduced pressure, and the residue was dissolved in 50 mL of DCM. The organic phase was washed with H_2O (3×20 mL), dried over Na_2SO_4, and concentrated under reduced pressure. Purification by column chromatography (gradient from DCM/MeOH 6:1 to DCM/MeOH 3:1) gave **4** (27.6 mg, 98 µmol, 38%) as a white powder.

R_f = 0.42 (SiO_2, DCM/MeOH 6:1; $KMnO_4$ stain). Analytical data (NMR, MS) matched those previously reported in literature.[3]

1.3. Synthesis of modified nucleotide dC^{Bio}TP

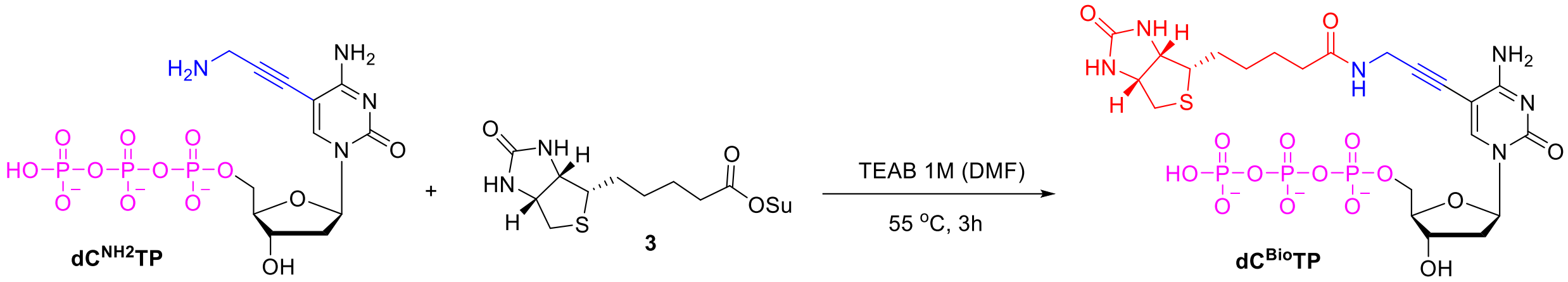

Scheme S3. Chemical synthesis of modified nucleotide **dC^{Bio}TP**.

5-[(D-Biotinylamido)prop-1-yn-1-yl]-2'-deoxycytidine 5'-*O*-triphosphate (dC^{Bio}TP)

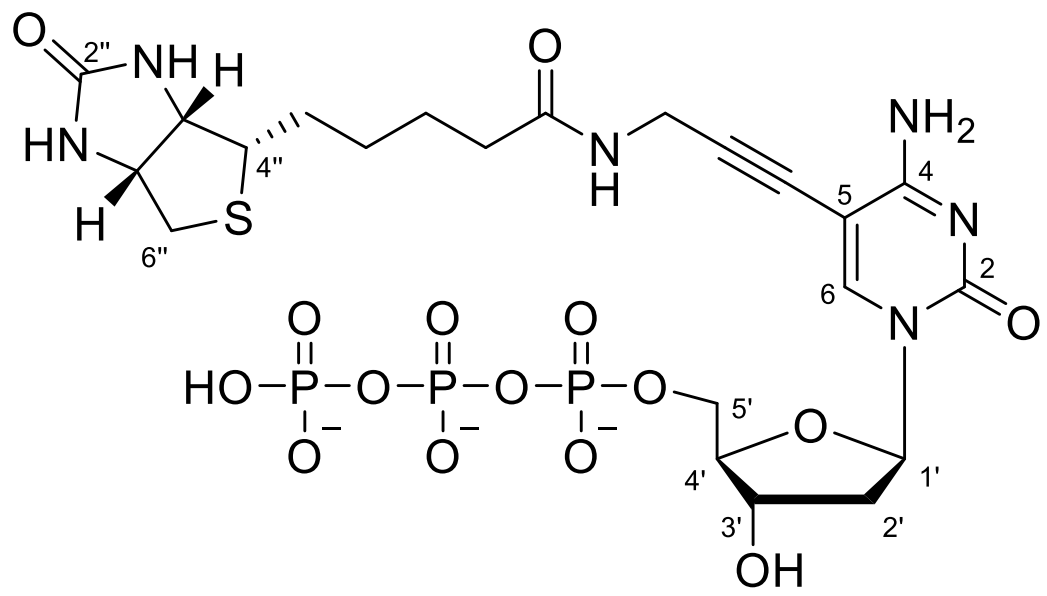

3 (37 mg, 0.11 mmol – previously dissolved in the minimum amount of DMF) was added to **dC^{NH2}TP** [4,5,6] (Et_3NH^+ salt, 15.1 mg, 18 µmol) in 1 M TEAB buffer (1 mL). The mixture was stirred at 55 °C for 3 h. The solvent was evaporated under reduced pressure, and the residue was co-distilled with water three times. HPLC purification (0.1 M TEAB in H_2O to 0.1 M TEAB in H_2O/60% CH_3CN), several co-distillations with water followed by freeze-drying from water gave **dC^{Bio}TP** (Et_3NH^+ salt, 8.1 mg, 7.7 µmol, 43%) as a white solid.

R_f = 0.72 (SiO_2, IPAV). ^{1}H NMR (600.1 MHz, D_2O): 1.36 – 1.44 (m, 2H, $CH_2C\mathbf{H_2}CH_2CH_2CO$); 1.56 (m, 1H, $C\mathbf{H_2}CH_2CH_2CH_2CO$); 1.64 – 1.75 (m, 3H, $C\mathbf{H_2}CH_2C\mathbf{H_2}CH_2CO$); 2.27 – 2.35 (m, 3H, H-2´a, $CH_2CH_2CH_2C\mathbf{H_2}CO$); 2.46 (ddd, 1H, J_{gem} = 14.0 Hz, $J_{2'b,1'}$ = 6.3 Hz, $J_{2'b,3'}$ = 4.1 Hz, H-2´b); 2.70 (d, 1H, J_{gem} = 13.1 Hz, H-6´´a); 2.91 (dd, 1H, J_{gem} = 13.1 Hz, $J_{6''b,6a''}$ = 5.0 Hz, H-6´´b); 3.25 (ddd, 1H, $J_{4'',CH2}$ = 8.8 and 6.0 Hz, $J_{4'',3a''}$ = 4.4 Hz, H-4´´); 4.14 – 4.24 (m, 5H, H-4´,5´, $CH_2C{\equiv}C$); 4.36 (dd, 1H, $J_{3a'',6a''}$ = 8.0 Hz, $J_{3a'',4''}$ = 4.4 Hz, H-3a´´); 4.53 (bdd, 1H, $J_{6a'',3a''}$ = 8.0 Hz, $J_{6a'',6''b}$ = 5.0 Hz, H-6a´´); 4.61 (m, 1H, H-3´); 6.25 (t, 1H, $J_{1',2'}$ = 6.5 Hz, H-1´); 8.20 (s, 1H, H-6); ^{13}C NMR (150.9 MHz, D_2O): 27.27 ($CH_2CH_2\mathbf{C}H_2CH_2CO$); 29.51 ($CH_2\mathbf{C}H_2CH_2CH_2CO$); 29.69 ($\mathbf{C}H_2CH_2CH_2CH_2CO$); 31.56 ($\mathbf{C}H_2C{\equiv}C$); 37.29 ($CH_2CH_2CH_2\mathbf{C}H_2CO$); 41.37 ($CH_2$-2´); 41.71 ($CH_2$-6´´); 57.38 (CH-4´´); 62.20 (CH-6a´´); 63.92 (CH-3a´´); 67.18 (d, $J_{C,P}$ = 5.5 Hz, CH_2-5´); 72.31 (CH-3´); 74.94 ($CH_2C{\equiv}\mathbf{C}$); 87.56 (d, $J_{C,P}$ = 8.7 Hz, CH-4´); 88.35 (CH-1´); 94.32 (C-5, $CH_2\mathbf{C}{\equiv}C$); 147.07 (CH-6); 157.78 (C-2); 166.99 (C-4); 167.19 (C-2´´); 179.16 ($CH_2CH_2CH_2CH_2\mathbf{C}O$); ^{31}P{^{1}H} NMR (202.4 MHz, D_2O): -22.59 (bt, 1P, $J_{\beta,\alpha} = J_{\beta,\gamma}$ = 19.9 Hz, P_β); -10.81 (d, 1P, $J_{\alpha,\beta}$ = 20.1 Hz, P_α); -10.17 (d, 1P, $J_{\gamma,\beta}$ = 19.8 Hz, P_γ). ESI MS *m*/*z* (rel. %) 745 (100) $[M-H]^-$; HR MS (ESI) for $C_{22}H_{32}O_{15}N_6P_3S$ $[M-H]^-$ calcd. 745.08647, found 745.08713.

1.4. Synthesis of modified nucleotide dC^{pegBio}TP

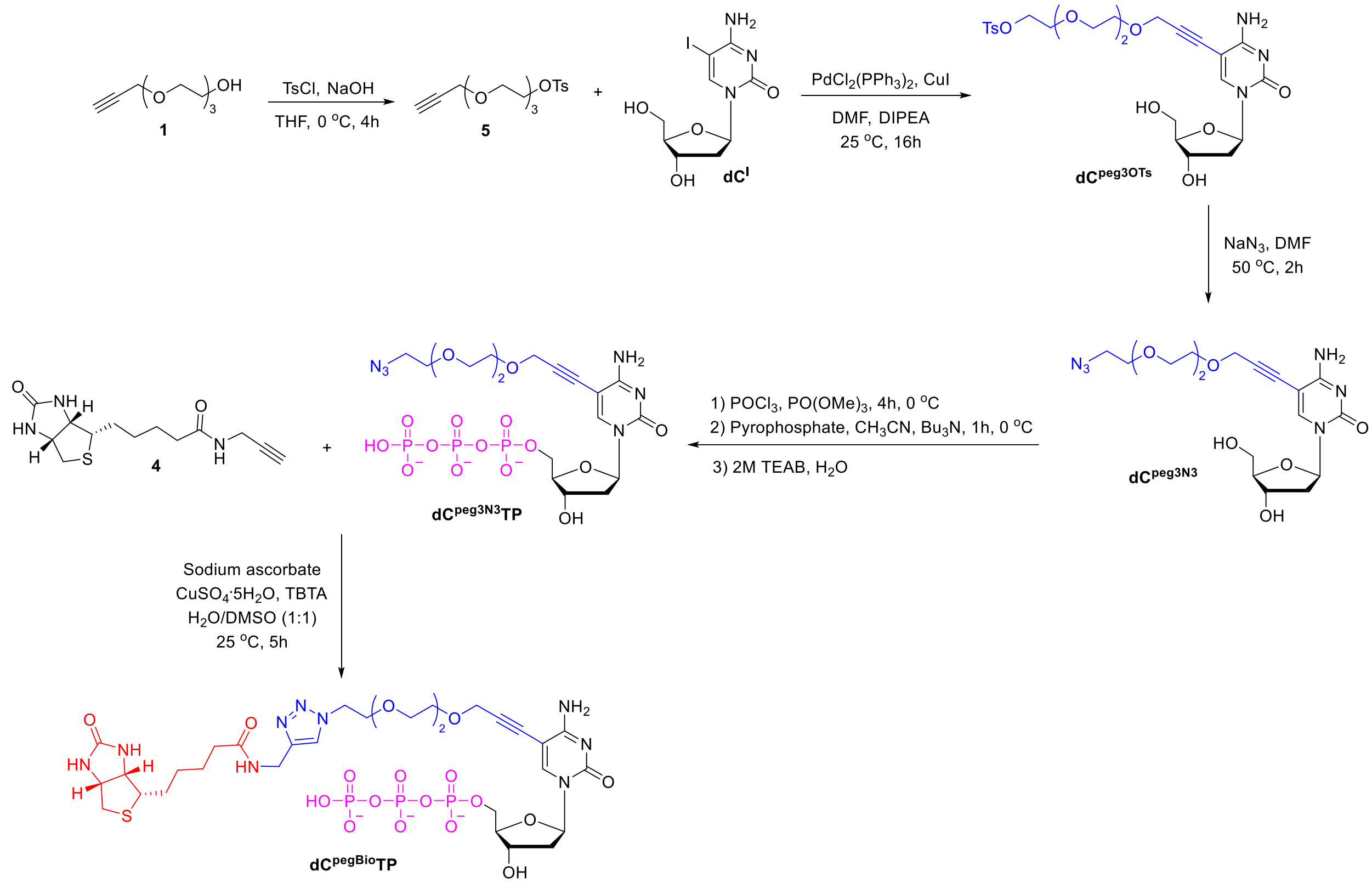

Scheme S4. Chemical synthesis of modified nucleotide **dC^{pegBio}TP**.

2-{2-[2-(prop-2-yn-1-yloxy)ethoxy]ethoxy}ethyl 4-methylbenzenesulfonate (5)[7]

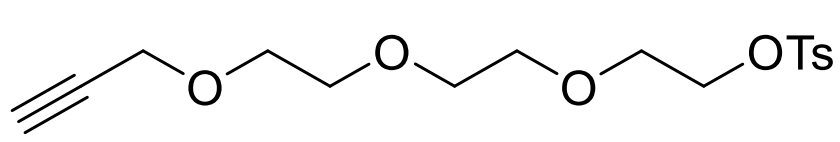

A solution of **1** (2.27 g, 12.06 mmol) in THF (15 mL) and aqueous NaOH (1.66 g, 17 mL, 2.5 M) was added to a solution of TsCl (4.13 g, 21.66 mmol) in THF (20 mL) at 0 °C. The mixture was stirred for 4 h, then DCM (150 mL) was added. The mixture was washed with H_2O (3×70 mL), the organic layer was collected, dried over Na_2SO_4 and concentrated under reduced pressure. Purification by column chromatography (cyclohexane/EtOAc 2:1) gave **5** (3.49 g, 10.19 mmol, 84%) as a pale-yellow oil.

R_f = 0.21 (SiO_2, cyclohexane/EtOAc 2:1; $KMnO_4$ stain). Analytical data (NMR, MS) matched those previously reported in literature.[7]

5-{2-[2-({2-prop-2-yn-1-yl}oxy)ethoxy]ethoxy}ethyl-4-methylbenzenesulfonate-2'-deoxycytidine (dC^{peg3OTs})[8]

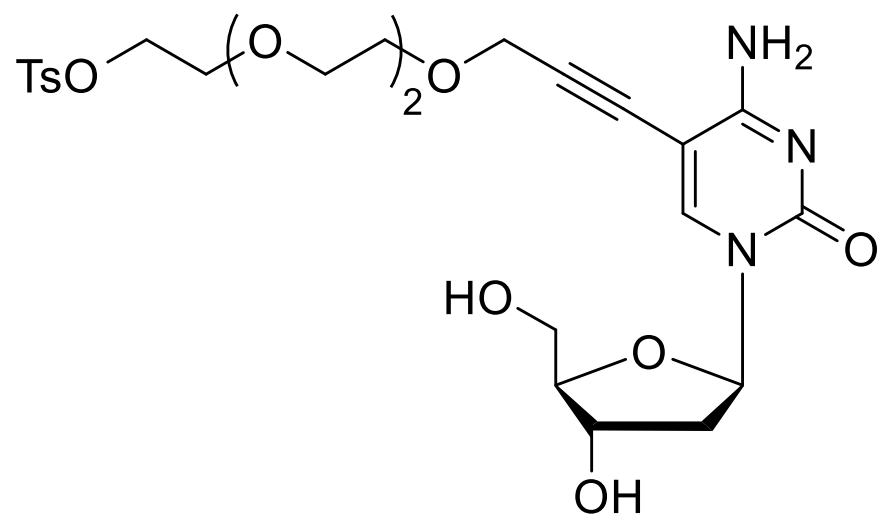

DMF (6 mL) was added to a vial containing **dC^{I}** (200 mg, 0.56 mmol), **5** (394.6 mg, 1.15 mmol), $PdCl_2(PPh_3)_2$ (23.6 mg, 33 µmol), and CuI (10.3 mg, 54 µmol). Then, DIPEA (0.4 mL, 2.3 mmol) was added dropwise, and the mixture was stirred at 25 °C for 16 h. The mixture was concentrated under reduced pressure, and the purification by column chromatography (DCM/MeOH 9:1) gave **dC^{peg3OTs}** (318 mg, 0.56 mmol, 89%) as an orange sticky oil.

R_f = 0.34 (SiO_2, DCM/MeOH 9:1). Analytical data (NMR, MS) matched those previously reported in literature.[8]

5-{3-[2-(2-{2-azidoethoxy}ethoxy)ethoxy]prop-1-yn-1-yl}-2'-deoxycytidine (dC^{peg3N3})[8]

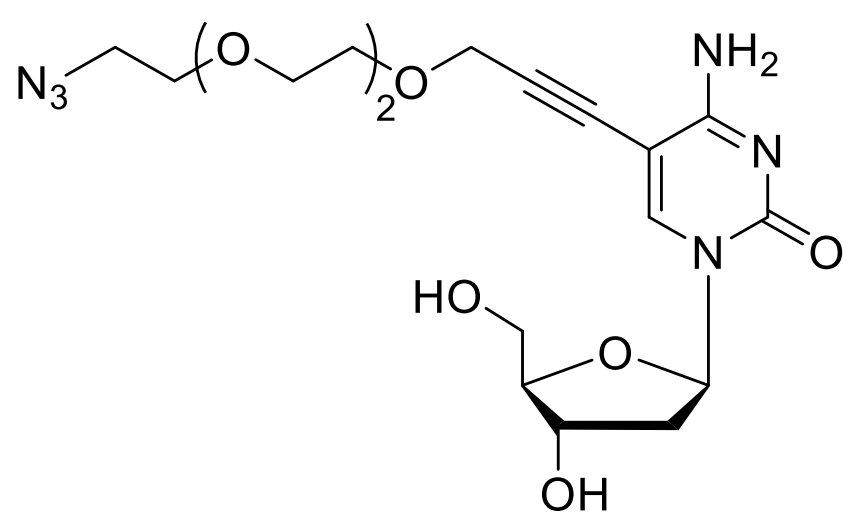

dC^{peg3OTs} (315 mg, 0.55 mmol) was dissolved in DMF (6 mL), followed by addition of NaN_3 (81.8 mg, 1.26 mmol). The mixture was stirred at 50 °C for 2 h, then concentrated under reduced pressure. Purification by column chromatography (DCM/MeOH 9:1) gave **dC^{peg3N3}** (193 mg, 0.44 mmol, 80%) as a pale-yellow sticky oil.

R_f = 0.42 (SiO_2, DCM/MeOH 9:1). Analytical data (NMR, MS) matched those previously reported in literature.[8]

5-{3-[2-(2-{2-azidoethoxy}ethoxy)ethoxy]prop-1-yn-1-yl}-2'-deoxycytidine 5'-*O*-triphosphate (dC^{peg3N3}TP)[8]

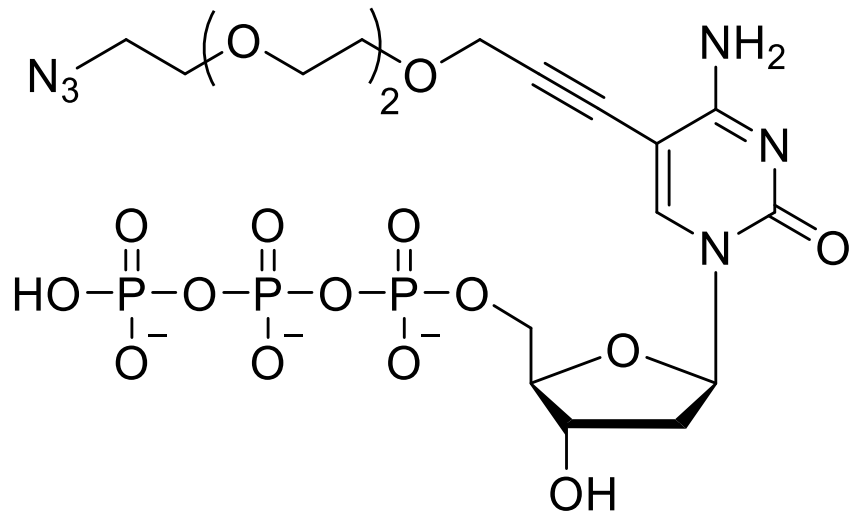

dC^{peg3N3} (42 mg, 96 µmol) was dried overnight at 25 °C. $PO(OMe)_3$ (0.6 mL) was added, the solution was cooled down to 0 °C, and $POCl_3$ (12 µL, 0.13 mmol) was added dropwise. The mixture was stirred at 0 °C for 4 h. Then, an ice-cooled solution of $(NHBu_3)_2H_2P_2O_7$ (284.7 mg, 0.2 mmol) and Bu_3N (65 µL, 0.27 mmol) in dry CH_3CN (1 mL) was added, and the mixture was stirred at 0 °C for another 1 h. The reaction was treated with 2 M aqueous TEAB buffer (2 mL), the solvents were evaporated under reduced pressure, and the residue was co-distilled with water three times. HPLC purification (0.1 M TEAB in H_2O to 0.1 M TEAB in H_2O/MeOH 1:1), several co-distillations with water followed by freeze-drying from water gave **dC^{peg3N3}TP** (Et_3NH^+ salt, 35.9 mg, 36 µmol, 38%) as a white sticky solid.

Analytical data (NMR, MS) matched those previously reported in literature.[8]

5-(3-{2-[2-(2-{[(D-Biotinylamido)methyl]-1*H*-1,2,3-triazol-1-yl}ethoxy)ethoxy]ethoxy}prop-1-yn-1-yl)-2'-deoxycytidine 5'-*O*-triphosphate (dC^{pegBio}TP)

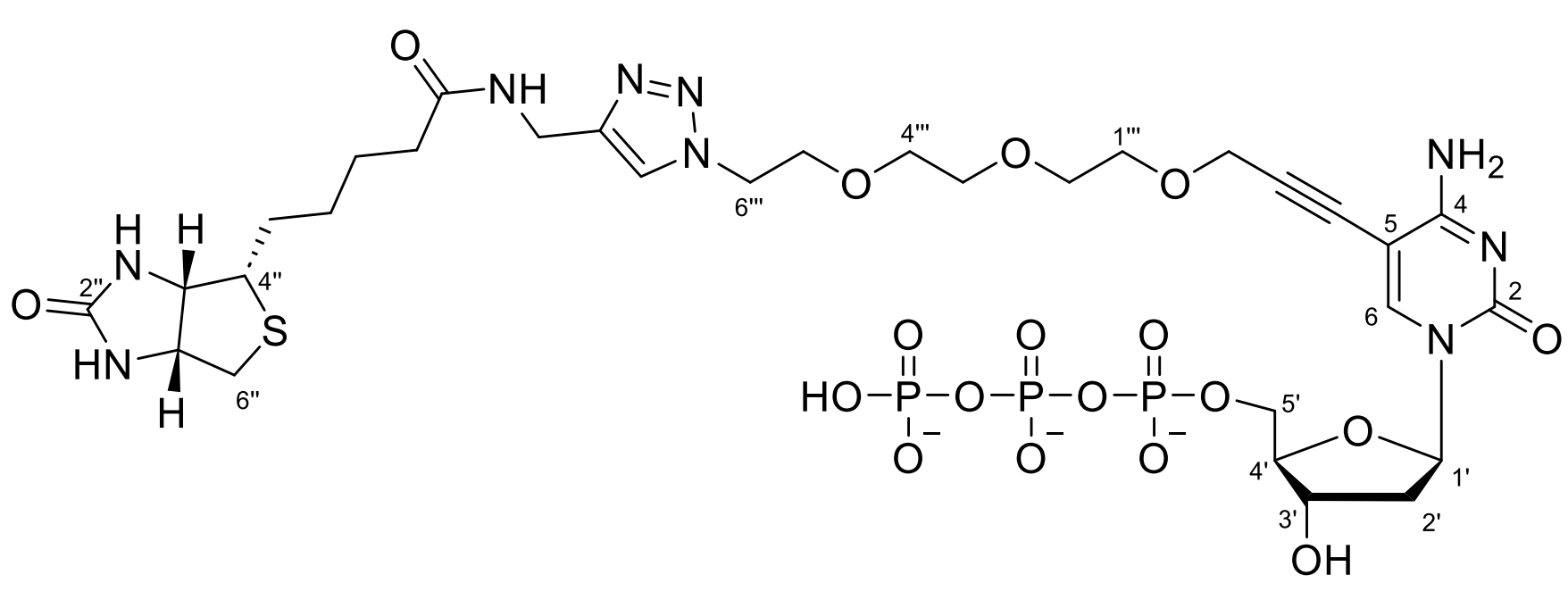

dC^{peg3N3}TP (Et_3NH^+ salt, 11.3 mg, 11 µmol) in H_2O (440 µL) and **4** (5.8 mg, 21 µmol) in DMSO (140 µL) were added to a vial, and the mixture was stirred. To this vial, a freshly prepared solution of $CuSO_4 \cdot 5H_2O$ (1.1 mg, 4.4 µmol) and TBTA (3.1 mg, 5.8 µmol) in DMSO (300 µL) was added. After 2 minutes of stirring, sodium ascorbate (1.4 mg, 7 µmol) was added, and the resulting mixture was stirred at 25 °C for 5 h. Then, the mixture was treated with 0.05 M solution of EDTA (1 mL), and freeze-dried from water. HPLC purification (0.1 M TEAB in H_2O to 0.1 M TEAB in H_2O/MeOH 1:1), several co-distillations with water, followed by freeze-drying from water gave **dC^{pegBio}TP** (Et_3NH^+ salt, 8 mg, 6.56 µmol, 60%) as a white solid.

^{1}H NMR (600.1 MHz, D_2O): 1.29 – 1.37 (m, 2H, $CH_2C\mathbf{H_2}CH_2CH_2CO$); 1.48 – 1.72 (m, 4H, $C\mathbf{H_2}CH_2C\mathbf{H_2}CH_2CO$); 2.28 (t, 2H, $J_{CH2,CH2}$ = 7.2 Hz, $CH_2CH_2CH_2C\mathbf{H_2}CO$); 2.30 (dt, 1H, J_{gem} = 14.1 Hz, $J_{2'a,1'} = J_{2'a,3'}$ = 6.7 Hz, H-2′a); 2.44 (ddd, 1H, J_{gem} = 14.1 Hz, $J_{2'b,1'}$ = 6.3 Hz, $J_{2'b,3'}$ = 4.0 Hz, H-2′b); 2.76 (d, 1H, J_{gem} = 13.1 Hz, H-6′′a); 2.97 (dd, 1H, J_{gem} = 13.1 Hz, $J_{6''b,6a''}$ = 5.0 Hz, H-6′′b); 3.26 (ddd, 1H, $J_{4'',CH2}$ = 9.1 and 5.7 Hz, $J_{4'',3a''}$ = 4.5 Hz, H-4′′); 3.60 – 3.69 (m, 6H, CH_2O-2′′′,3′′′,4′′′); 3.76 (m, 2H, CH_2O-1′′′); 3.95 (t, 2H, $J_{CH2,CH2}$ = 5.0 Hz, CH_2O-5′′′); 4.17 – 4.26 (m, 3H, H-4′,5′); 4.37 (dd, 1H, $J_{3a'',6a''}$ = 7.9 Hz, $J_{3a'',4''}$ = 4.5 Hz, H-3a′′); 4.43 and 4.46 (2×d, 2×1H, J_{gem} = 15.5 Hz, $NHCH_2$); 4.47 (s, 2H, $CH_2C{\equiv}C$); 4.57 – 4.64 (m, 4H, H-6a′′, H-3′,CH_2O-6′′′); 6.24 (t, 1H, $J_{1',2'}$ = 6.6 Hz, H-1′); 7.95 (s, 1H, H-5-triazolyl); 8.17 (s, 1H, H-6); ^{13}C NMR (150.9 MHz, D_2O): 27.07 ($CH_2CH_2\mathbf{C}H_2CH_2CO$); 29.60 ($CH_2\mathbf{C}H_2CH_2CH_2CO$); 29.78 ($\mathbf{C}H_2CH_2CH_2CH_2CO$); 36.33 ($NHCH_2$); 37.29 ($CH_2CH_2CH_2\mathbf{C}H_2CO$); 41.29 ($CH_2$-2′); 41.70 ($CH_2$-6′′); 52.05 ($CH_2O$-6′′′); 57.33 (CH-4′′); 60.58 ($\mathbf{C}H_2C{\equiv}C$); 62.24 (CH-6a′′); 64.00 (CH-3a′′); 67.13 (d, $J_{C,P}$ = 5.6 Hz, CH_2-5′); 70.74 (CH_2O-5′′′); 70.84 (CH_2O-1′′′); 71.48 and 71.71 (CH_2O-2′′′,3′′′,4′′′); 72.25 (CH-3′); 74.00 ($CH_2C{\equiv}\mathbf{C}$); 87.56 (d, $J_{C,P}$ = 8.8 Hz, CH-4′); 88.29 (CH-1′); 93.62 ($CH_2\mathbf{C}{\equiv}C$); 93.84 (C-5); 126.30 (CH-5-triazolyl); 146.68 (C-4-triazolyl); 147.17 (CH-6); 157.92 (C-2); 167.00 (C-4); 167.28 (C-2′′); 178.60 ($CH_2CH_2CH_2CH_2\mathbf{C}O$); ^{31}P{^{1}H} NMR (202.4 MHz, D_2O): -22.39 (t, 1P, $J_{\beta,\alpha} = J_{\beta,\gamma}$ = 20.4 Hz, P_β); -10.76 (d, 1P, $J_{\alpha,\beta}$ = 20.1 Hz, P_α); -8.57 (bs, 1P, P_γ). ESI MS *m/z* (rel. %) 958 (100) $[M-H]^-$; HR MS (ESI) for $C_{31}H_{47}O_{18}N_9P_3S$ $[M-H]^-$; calcd. 958.19781, found 958.19803.

2. Experimental section – Biochemistry

General remarks

Mass spectra of oligonucleotides were measured on UltrafleXtreme MALDI-TOF/TOF (Bruker) mass spectrometer with 1 kHz smartbeam II laser technology by the MS service at IOCB. The matrix consisted of 3-hydroxypicolinic acid (HPA) / picolinic acid (PA) / ammonium tartrate in ratio 9:1:1. LC-ESI-MS spectra were acquired on UPLC Agilent 1290 Infinity II Bio system with DAD detector and mass spectrometer MSD XT (operating in negative mode). LC-ESI-MS analysis of oligonucleotides was carried out using a linear gradient of 12 mM Et_3N and 300 mM HFIP in H_2O to 12 mM Et_3N and 300 mM HFIP in MeOH in 30 min, with bioZen™ Oligo LC column (1.7 µm, 50 × 2.1 mm; Phenomenenx). Mass spectra were deconvoluted using OpenLAB CDS CS LC/MS Deconvolution Bioanalysis software.[9] UV-Vis spectra (ONs concentration) were measured at room temperature on NanoPhotometer® N60 (Implen GmbH). Samples were evaporated on CentriVap Vacuum Concentrator system (Labconco) and/or freeze-dried from water on FreeZone 2.5 L freeze dryer (Labconco). Synthetic oligonucleotides (unmodified primers, 5'-end labelled primers with 6-carboxyfluorescein (6-FAM) or Cy5, unmodified templates, 5'-phosphorylated template; for sequences see Table S1) were purchased from either Generi Biotech (Czech Republic) or Eurofins Genomics (KRD). 5'-Dual biotinylated templates were purchased from Biomers.net GmbH (Germany) (Table S1). Natural nucleoside triphosphates (dATP, dGTP, dTTP, dCTP) were purchased from either ThermoFisher Scientific or New England Biolabs. The recombinant HaloTag® standard (GST-Halotag) protein was purchased from EastPort LifeScience (Czech Republic); the recombinant SAvPhire™ Monomeric Streptavidin (mSA) protein was purchased from Sigma-Aldrich (Czech Republic); the recombinant SAvPhire Monomeric Streptavidin (mSA) iFluor™ 647 and recombinant SAvPhire Monomeric Streptavidin (mSA) iFluor™ 555 proteins were purchased from Sigma-Aldrich (Czech Republic). KOD XL DNA polymerase and corresponding polymerase reaction buffer were purchased from Merck Life Science. Shrimp Alkaline Phosphatase (rSAP) was purchased from New England Biolabs. Terminal Deoxynucleotidyl Transferase (TdT) and corresponding reaction buffer were purchased from either ThermoFisher Scientific or New England Biolabs. Lambda exonuclease was purchased from New England Biolabs. Streptavidin magnetic particles (SMB) were purchased from Merck Life Science, QIAQuick® Nucleotide Removal Kit (QIAGEN) was purchased from BioTech (Czech Republic). Milli-Q water was used for all experiments. Samples after PEX reactions were analyzed by 12.5% PAGE (acrylamide/bisacrylamide 19:1, 25% urea), under denaturing conditions (1 h, 40 mA, 50 °C, 1X TBE buffer pH 8). Samples after SNI and PEX reactions, and reaction with TdT were analyzed by 20% PAGE (acrylamide/bisacrylamide, 19:1, 40% urea) under denaturing conditions (1 h, 40 mA, 50 °C, 1X TBE buffer pH 8). 2X PAGE stop solution used after PEX reactions contains: 95% [v/v] formamide, 0.5 mM EDTA, 0.025% [w/v] bromophenol blue, 0.025% [w/v] SDS in Milli-Q water. Reactions with recombinant HaloTag® standard protein and/or recombinant SAvPhire™ Monomeric Streptavidin protein, or recombinant SAvPhire Monomeric Streptavidin iFluor™ 555 protein, were performed in PBS1 buffer (1X PBS

– 137 mM NaCl, 2.7 mM KCl, 10 mM Na_2HPO_4, 1.8 mM KH_2PO_4 – plus 100 mM NaCl, 1 mM DTT, 20% [v/v] glycerol, pH 7.2 at 25 °C) or PBS2 buffer (1X PBS – 137 mM NaCl, 2.7 mM KCl, 10 mM Na_2HPO_4, 1.8 mM KH_2PO_4 – pH 7.4 at 25 °C). Samples after conjugation with recombinant HaloTag® standard protein were analyzed by 10%, 12% or 15% SDS PAGE (acrylamide/bisacrylamide 37.5:1. 5% stacking gel; 10%, 12% or 15% resolving gel), under denaturing conditions (1 h, 200 V, 1X TRIS-glycine-SDS buffer – 25 mM TRIS, 192 mM glycine, 0.1% [w/v] SDS, pH 8.6). 2X VPS loading buffer used for SDS denaturing PAGE contains: 0.05 M Tris-HCl, 17% [v/v] glycerol, 16 mM mercaptoethanol, 3.5% [w/v] SDS, 0.025% [w/v] bromophenol blue in Milli-Q water, pH 6.8. The protein ladder used for SDS denaturing PAGE was Color Prestained Protein Standard, Broad Range (10-250 kDa), purchased from New England Biolabs. Samples after reaction with recombinant SAvPhire™ Monomeric Streptavidin protein were analyzed by 10%, 12% or 15% protein PAGE (acrylamide/bisacrylamide 19:1. 5% stacking gel; 10%, 12% or 15% resolving gel), under native conditions (4 h, 100 V, with cooling, 0.5X TBE buffer). Samples after reaction with combination of recombinant SAvPhire™ Monomeric Streptavidin protein or recombinant SAvPhire Monomeric Streptavidin iFluor™ 555 protein and recombinant HaloTag® standard protein were analyzed by 7% protein PAGE (acrylamide/bisacrylamide 19:1. 5% stacking gel; 7% resolving gel), under native conditions (4 h, 100 V, with cooling, 0.5X TBE buffer). All PAGE gels were analyzed by fluorescence imaging of 6-FAM or Cy5 labels at 5'-ends using Amersham Typhoon Biomolecular Imager (Cytiva). Conversions were calculated using Fiji software.[10] Fluorescent Nickel-plate binding affinity assay was performed in 96-well Pierce Nickel-coated Plate. Fluorescence intensity was measured at 633ex/678em nm using a Spark multimode microplate reader (Tecan). Dissociation constant (K_D) was calculated using the GraphPad Prism 8.01 software. Data were fitted to a specific binding with a Hill Slope non-linear regression model. The pH values of used reaction buffers were determined using the inoLab pH 720. Other chemicals were of analytical grade.

Modified nucleotides used in the study

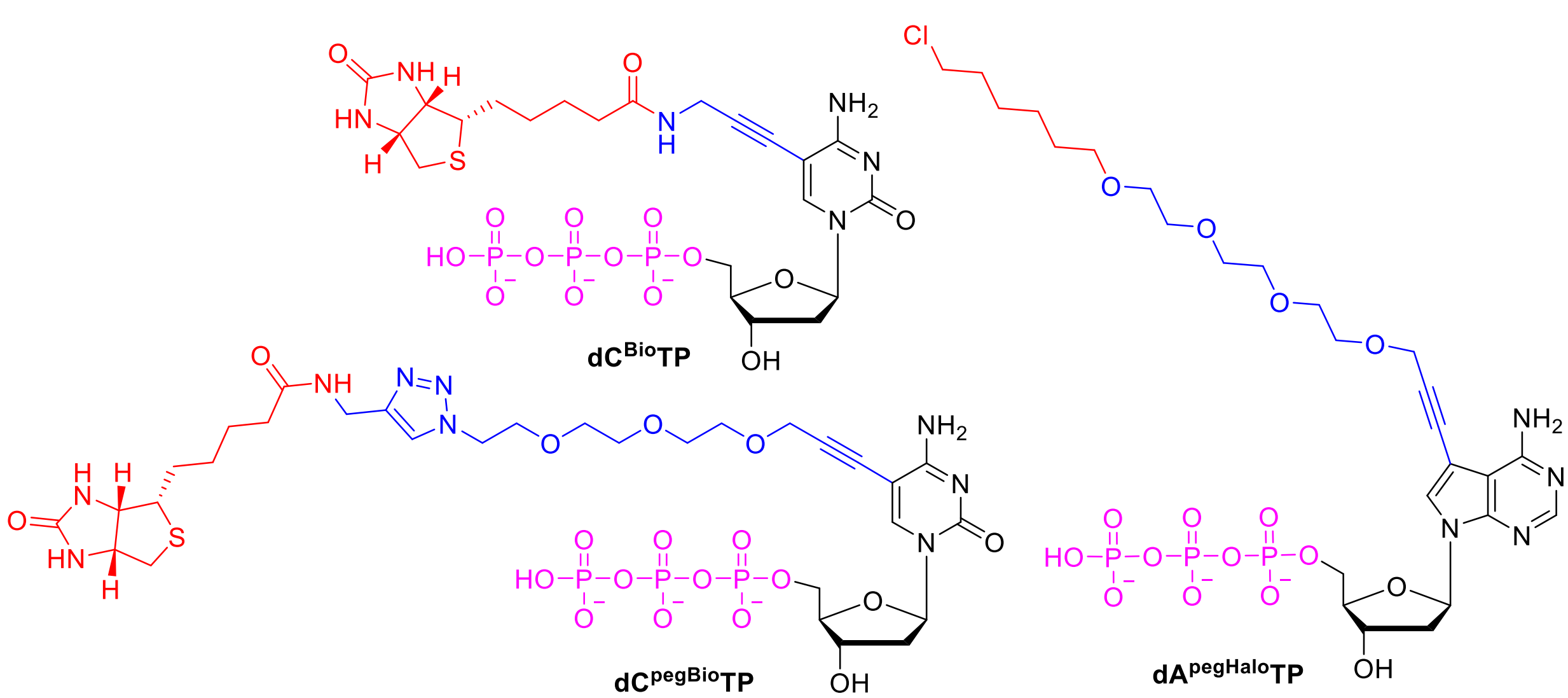

2.1. List of sequences of primers, templates and oligonucleotides used in the study

Table S1. List of sequences of primers and templates used in this study

ON Name	Length (nt)	Sequence (5'→3')
**Prim248short**	15	CATGGGCGGCATGGG
**Prim248short-Cy5** [a] **/ FAM** [b]	15	CATGGGCGGCATGGG
**Oligo 1A**	19	CCCTCCCATGCCGCCCATG
**Oligo 1A-bio** [c]	19	CCCTCCCATGCCGCCCATG
**Oligo 1C**	19	CCCGCCCATGCCGCCCATG
**Oligo 1C-bio** [c]	19	CCCGCCCATGCCGCCCATG
**Oligo 2A_10**	30	CCCTCGACGACGACTCCCATGCCGCCCATG
**Oligo 2A_10-bio** [c]	30	CCCTCGACGACGACTCCCATGCCGCCCATG
**T30-10**	30	CCCGACCACCACCCTCCCATGCCGCCCATG
**Prb4basII-bio** [c]	31	CTAGCATGAGCTCAGTCCCATGCCGCCCATG
**Oligo 2A_20**	40	CCCTACGACGACGACGACGACTCCCATGCCGCCCATG
**Oligo 2A_20-bio** [c]	40	CCCTACGACGACGACGACGACTCCCATGCCGCCCATG
**T40-20**	40	CCCGACCACCACCACCACCACTCCCATGCCGCCCATG
**Oligo 2A_30**	50	CCCTGACGACGACGACGACGACGACGACGACTCCCAT GCCGCCCATG
**Oligo 2A_30-bio** [c]	50	CCCTGACGACGACGACGACGACGACGACGACTCCCAT GCCGCCCATG
**T50-30**	50	CCCGCACCACCACCACCACCACCACCACCACTCCCATG CCGCCCATG
**T60-40**	60	CCCGACCACCACCACACCACCACCACCACCACCACCACCA CCACTCCCATGCCGCCCATG
**T70-50**	70	CCCGACACCACCACACCACCACCACACCACCACCACCACC ACCACCACCACCACTCCCATGCCGCCCATG
**T70-50 5'-P** [d]	70	CCCGACACCACCACACCACCACCACACCACCACCACCACC ACCACCACCACCACTCCCATGCCGCCCATG

[a] 5'-(Cy5)-labelled; [b] 5'-(6-FAM)-labelled; [c] 5'-dual biotinylated; [d] 5'-phosphorylated; in the template ONs the segments forming duplex with the primer are underlined.

Table S2. List of synthetized ssDNA/dsDNA

Name	Sequence in 5'→3' (primer regions underlined)
**17ON_A^{Natural}** [a]	CATGGGCGGCATGGG**A**C
**17ON_A^{pegHalo}** [a]	CATGGGCGGCATGGG**A**^{pegHalo}C
**19ON_A^{Natural}** [a]	CATGGGCGGCATGGG**A**GGG
**19ON_A^{pegHalo}**	CATGGGCGGCATGGG**A**^{pegHalo}GGG
**19ON_A^{pegHalo}** [a]	CATGGGCGGCATGGG**A**^{pegHalo}GGG
**19ON_C^{Natural}** [a]	CATGGGCGGCATGGG**C**GGG
**19ON_C^{Bio}**	CATGGGCGGCATGGG**C**^{Bio}GGG
**19ON_C^{Bio}** [a]	CATGGGCGGCATGGG**C**^{Bio}GGG
**19ON_C^{pegBio}**	CATGGGCGGCATGGG**C**^{pegBio}GGG

**19ON_C**^{pegBio} [a]	CATGGGCGGCATGGG**C**^{pegBio}GGG
**30ON_A**^{Natural} [a]	CATGGGCGGCATGGG**A**GTCGTCGTCG**A**GGG
**30ON_A**^{pegHalo}	CATGGGCGGCATGGG**A**^{pegHalo}GTCGTCGTCG**A**^{pegHalo}GGG
**30ON_A**^{pegHalo} [a]	CATGGGCGGCATGGG**A**^{pegHalo}GTCGTCGTCG**A**^{pegHalo}GGG
**30ON_A**^{Natural}**C**^{Natural} [a]	CATGGGCGGCATGGG**A**GGGTGGTGGT**C**GGG
**30ON_A**^{pegHalo}**C**^{Bio} [a]	CATGGGCGGCATGGG**A**^{pegHalo}GGGTGGTGGT**C**^{Bio}GGG
**30ON_A**^{pegHalo}**C**^{pegBio} [a]	CATGGGCGGCATGGG**A**^{pegHalo}GGGTGGTGGT**C**^{pegBio}GGG
**31ON_A**^{Natural} [a]	CATGGGCGGCATGGG**A**CTGAGCTCATGCTAG
**31ON_A**^{pegHalo} [a]	CATGGGCGGCATGGG**A**^{pegHalo}CTGAGCTCATGCTAG
**32ON_A**^{pegHalo}**C**^{pegBio} [a]	CATGGGCGGCATGGG**A**^{pegHalo}CTGAGCTCATGCTAG**C**^{pegBio}
**40ON_A**^{Natural} [a]	CATGGGCGGCATGGG**A**GTCGTCGTCGTCGTCGTCGT**A**GGG
**40ON_A**^{pegHalo}	CATGGGCGGCATGGG**A**^{pegHalo}GTCGTCGTCGTCGTCGTCGT**A**^{pegHalo}GGG
**40ON_A**^{pegHalo} [a]	CATGGGCGGCATGGG**A**^{pegHalo}GTCGTCGTCGTCGTCGTCGT**A**^{pegHalo}GGG
**40ON_A**^{Natural}**C**^{Natural} [a]	CATGGGCGGCATGGG**A**GTGGTGGTGGTGGTGGTGGT**C**GGG
**40ON_A**^{pegHalo}**C**^{Bio} [a]	CATGGGCGGCATGGG**A**^{pegHalo}GTGGTGGTGGTGGTGGTGGT**C**^{Bio}GGG
**40ON_A**^{pegHalo}**C**^{pegBio} [a]	CATGGGCGGCATGGG**A**^{pegHalo}GTGGTGGTGGTGGTGGTGGT**C**^{pegBio}GGG
**50ON_A**^{Natural} [a]	CATGGGCGGCATGGG**A**GTCGTCGTCGTCGTCGTCGTCGTCGTC**A**GGG
**50ON_A**^{pegHalo}	CATGGGCGGCATGGG**A**^{pegHalo}GTCGTCGTCGTCGTCGTCGTCGTCGTC**A**^{pegHalo}GGG
**50ON_A**^{pegHalo} [a]	CATGGGCGGCATGGG**A**^{pegHalo}GTCGTCGTCGTCGTCGTCGTCGTCGTC**A**^{pegHalo}GGG
**50ON_A**^{Natural}**C**^{Natural} [a]	CATGGGCGGCATGGG**A**GTGGTGGTGGTGGTGGTGGTGGTGGTG**C**GGG
**50ON_A**^{pegHalo}**C**^{Bio} [a]	CATGGGCGGCATGGG**A**^{pegHalo}GTGGTGGTGGTGGTGGTGGTGGTGGTGGTG**C**^{Bio}GGG
**50ON_A**^{pegHalo}**C**^{pegBio} [a]	CATGGGCGGCATGGG**A**^{pegHalo}GTGGTGGTGGTGGTGGTGGTGGTGGTGGTG**C**^{pegBio}GGG
**60ON_A**^{Natural}**C**^{Natural} [a]	CATGGGCGGCATGGG**A**GTGGTGGTGGTGGTGGTGGTGGTGGTGTGGTGGTGGT**C**GGG
**60ON_A**^{pegHalo}**C**^{Bio} [a]	CATGGGCGGCATGGG**A**^{pegHalo}GTGGTGGTGGTGGTGGTGGTGGTGGTGGTGTGGTGGTGGT**C**^{Bio}GGG
**60ON_A**^{pegHalo}**C**^{pegBio} [a]	CATGGGCGGCATGGG**A**^{pegHalo}GTGGTGGTGGTGGTGGTGGTGGTGGTGGTGTGGTGGTGGT**C**^{pegBio}GGG
**70ON_A**^{Natural}**C**^{Natural} [a]	CATGGGCGGCATGGG**A**GTGGTGGTGGTGGTGGTGGTGGTGGTGTGGTGGTGGTGTGGTGGTGT**C**GGG
**70ON_A**^{pegHalo}**C**^{Bio} [a]	CATGGGCGGCATGGG**A**^{pegHalo}GTGGTGGTGGTGGTGGTGGTGGTGGTGGTGTGGTGGTGGTGTGGTGGTGT**C**^{Bio}GGG
**70ON_A**^{pegHalo}**C**^{pegBio} [a]	CATGGGCGGCATGGG**A**^{pegHalo}GTGGTGGTGGTGGTGGTGGTGGTGGTGGTGTGGTGGTGGTGTGGTGGTGT**C**^{pegBio}GGG
**19DNA_A**^{Natural} [a]	CATGGGCGGCATGGG**A**GGG
**19DNA_A**^{pegHalo} [a]	CATGGGCGGCATGGG**A**^{pegHalo}GGG
**19DNA_C**^{Natural} [a]	CATGGGCGGCATGGG**C**GGG
**19DNA_C**^{Bio} [a]	CATGGGCGGCATGGG**C**^{Bio}GGG

**19DNA_C$^{pegBio\ a}$**	CATGGGCGGCATGGG**C**^{pegBio}GGG
**30DNA_A$^{Natural\ a}$**	CATGGGCGGCATGGG**A**GTCGTCGTCG**A**GGG
**30DNA_A$^{pegHalo\ a}$**	CATGGGCGGCATGGG**A**^{pegHalo}GTCGTCGTCG**A**^{pegHalo}GGG
**30DNA_A^{Natural}C$^{Natural\ b}$**	CATGGGCGGCATGGG**A**GGGTGGTGGT**C**GGG
**30DNA_A^{pegHalo}C$^{Bio\ a,b}$**	CATGGGCGGCATGGG**A**^{pegHalo}GGGTGGTGGT**C**^{Bio}GGG
**30DNA_A^{pegHalo}C$^{pegBio\ a,b}$**	CATGGGCGGCATGGG**A**^{pegHalo}GGGTGGTGGT**C**^{pegBio}GGG
**40DNA_A$^{Natural\ a}$**	CATGGGCGGCATGGG**A**GTCGTCGTCGTCGTCGTCGT**A**GGG
**40DNA_A$^{pegHalo\ a}$**	CATGGGCGGCATGGG**A**^{pegHalo}GTCGTCGTCGTCGTCGTCGT**A**^{pegHalo}GGG
**40DNA_A^{Natural}C$^{Natural\ b}$**	CATGGGCGGCATGGG**A**GTGGTGGTGGTGGTGGTGGT**C**GGG
**40DNA_A^{pegHalo}C$^{Bio\ a,b}$**	CATGGGCGGCATGGG**A**^{pegHalo}GTGGTGGTGGTGGTGGTGGT**C**^{Bio}GGG
**40DNA_A^{pegHalo}C$^{pegBio\ a,b}$**	CATGGGCGGCATGGG**A**^{pegHalo}GTGGTGGTGGTGGTGGTGGT**C**^{pegBio}GGG
**50DNA_A$^{Natural\ a}$**	CATGGGCGGCATGGG**A**GTCGTCGTCGTCGTCGTCGTCGTCGTCG TCAGGG
**50DNA_A$^{pegHalo\ a}$**	CATGGGCGGCATGGG**A**^{pegHalo}GTCGTCGTCGTCGTCGTCGTC GTCGTC**A**^{pegHalo}GGG
**50DNA_A^{Natural}C$^{Natural\ b}$**	CATGGGCGGCATGGG**A**GTGGTGGTGGTGGTGGTGGTGGTGGTG GTG**C**GGG
**50DNA_A^{pegHalo}C$^{Bio\ a,b}$**	CATGGGCGGCATGGG**A**^{pegHalo}GTGGTGGTGGTGGTGGTGGTGGTG GTGGTG**C**^{Bio}GGG
**50DNA_A^{pegHalo}C$^{pegBio\ a,b}$**	CATGGGCGGCATGGG**A**^{pegHalo}GTGGTGGTGGTGGTGGTGGTGGTG GTGGTG**C**^{pegBio}GGG
**60DNA_A^{Natural}C$^{Natural\ b}$**	CATGGGCGGCATGGG**A**GTGGTGGTGGTGGTGGTGGTGGTGGTG GTGTGGTGGTGGT**C**GGG
**60DNA_A^{pegHalo}C$^{Bio\ a,b}$**	CATGGGCGGCATGGG**A**^{pegHalo}GTGGTGGTGGTGGTGGTGGTGGTG GTGGTGTGGTGGTGGT**C**^{Bio}GGG
**60DNA_A^{pegHalo}C$^{pegBio\ a,b}$**	CATGGGCGGCATGGG**A**^{pegHalo}GTGGTGGTGGTGGTGGTGGTGGTG GTGGTGTGGTGGTGGT**C**^{pegBio}GGG
**70DNA_A^{Natural}C$^{Natural\ b}$**	CATGGGCGGCATGGG**A**GTGGTGGTGGTGGTGGTGGTGGTGGTG GTGTGGTGGTGGTGTGGTGGTGT**C**GGG
**70DNA_A^{pegHalo}C$^{Bio\ a,b}$**	CATGGGCGGCATGGG**A**^{pegHalo}GTGGTGGTGGTGGTGGTGGTGGTG GTGGTGTGGTGGTGGTGTGGTGGTGT**C**^{Bio}GGG
**70DNA_A^{pegHalo}C$^{pegBio\ a,b}$**	CATGGGCGGCATGGG**A**^{pegHalo}GTGGTGGTGGTGGTGGTGGTGGTG GTGGTGTGGTGGTGGTGTGGTGGTGT**C**^{pegBio}GGG

[a] 5'-(Cy5)-labelled; [b] 5'-(6-FAM)-labelled; ON – single stranded DNA; DNA – double stranded DNA.

2.2. Primer Extension (PEX) – Single incorporation of one modified dN^{X}TP

2.2.1. Single incorporation of dA^{pegHalo}TP using 19-mer template – Analytical scale

The reaction mixture (20 µL) contained 5'-(Cy5)-labelled **Prim248short** (3 µM, 1 µL), **Oligo 1A** (3 µM, 1.5 µL), KOD XL DNA polymerase (0.025 U/µL, 1 µL), natural dGTP (4 mM, 1 µL), either natural dATP or modified **dA^{pegHalo}TP** (4 mM, 1 µL), and enzyme reaction buffer (10X, 2 µL) as supplied by the manufacturer. The reaction mixture was incubated for 30 min at 60 °C in a thermal cycler, stopped by addition of PAGE stop solution (20 µL), and heated for 5 min at 95 °C.

Samples were separated by use of 12.5% denaturing PAGE and visualized using fluorescence imaging (Figure S1).

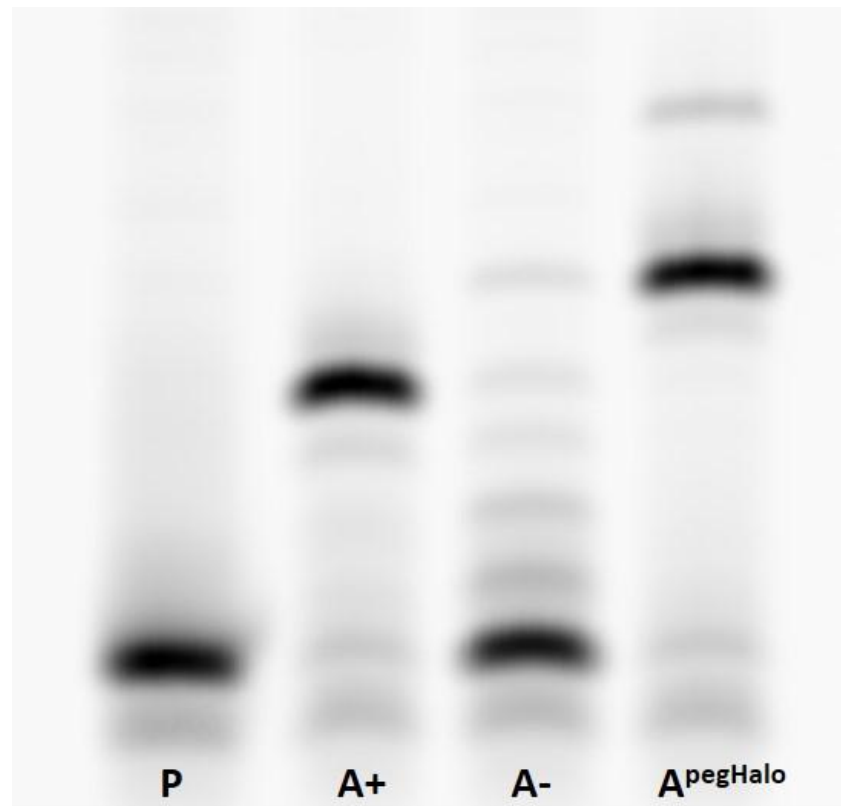

Figure S1. Denaturing PAGE analysis of PEX reaction with **dA^{pegHalo}TP** using KOD XL DNA polymerase: (P) primer **Prim248short** 5'-(Cy5)-labelled; template **Oligo 1A**: (A+): dATP, dGTP; (A-): dGTP; (A^{pegHalo}): **dA^{pegHalo}TP**, dGTP.

2.2.2. Single incorporation of dC^{X}TP using 19-mer template – Analytical scale

The reaction mixture (20 µL) contained 5'-(Cy5)-labelled **Prim248short** (3 µM, 1 µL), **Oligo 1C** (3 µM, 1.5 µL), KOD XL DNA polymerase (0.025 U/µL, 1 µL), natural dGTP (4 mM, 1 µL), either natural dCTP or one of modified **dC^{X}TP** (X = Bio, pegBio; 4 mM, 1 µL), and enzyme reaction buffer (10X, 2 µL) as supplied by the manufacturer. The reaction mixture was incubated for 30 min at 60 °C in a thermal cycler, stopped by addition of PAGE stop solution (20 µL), and heated for 5 min at 95 °C. Samples were separated by use of 12.5% denaturing PAGE and visualized using fluorescence imaging (Figure S2).

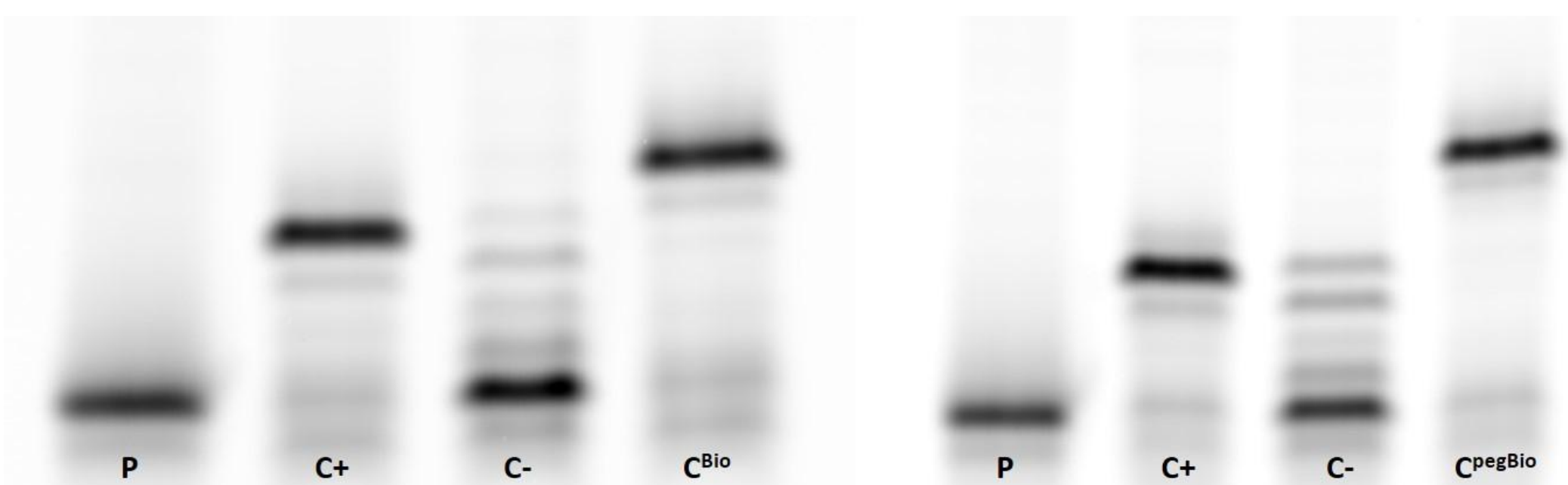

Figure S2. Denaturing PAGE analysis of PEX reaction with **dC^{X}TP** (X = Bio, pegBio) using KOD XL DNA polymerase: (P) primer **Prim248short** 5'-(Cy5)-labelled; template **Oligo 1C**: (C+): dCTP, dGTP; (C-): dGTP; (C^{Bio}): **dC^{Bio}TP**, dGTP; (C^{pegBio}): **dC^{pegBio}TP**, dGTP.

2.3. Primer Extension (PEX) – Multiple incorporation of modified dN^{X}TPs

2.3.1. Multiple incorporation of dA^{pegHalo}TP using 30-mer, 40-mer and 50-mer templates – Analytical scale

The reaction mixture (20 µL) contained 5'-(Cy5)-labelled **Prim248short** (3 µM, 1 µL), one of templates **Oligo 2A_10** / **Oligo 2A_20** / **Oligo 2A_30** (3 µM, 1.5 µL), KOD XL DNA polymerase

(0.5 U/µL, 0.6 µL), natural dGTP, dCTP and dTTP (1 mM, 1 µL), either natural dATP (1 mM, 1 µL) or modified **dA^{pegHalo}TP** (0.5 mM, 1 µL), and enzyme reaction buffer (10X, 2 µL) as supplied by the manufacturer. The reaction mixture was incubated for 10 min at 60 °C in a thermal cycler, stopped by addition of PAGE stop solution (20 µL), and heated for 5 min at 95 °C. Samples were separated by use of 12.5% denaturing PAGE and visualized using fluorescence imaging (Figure S3).

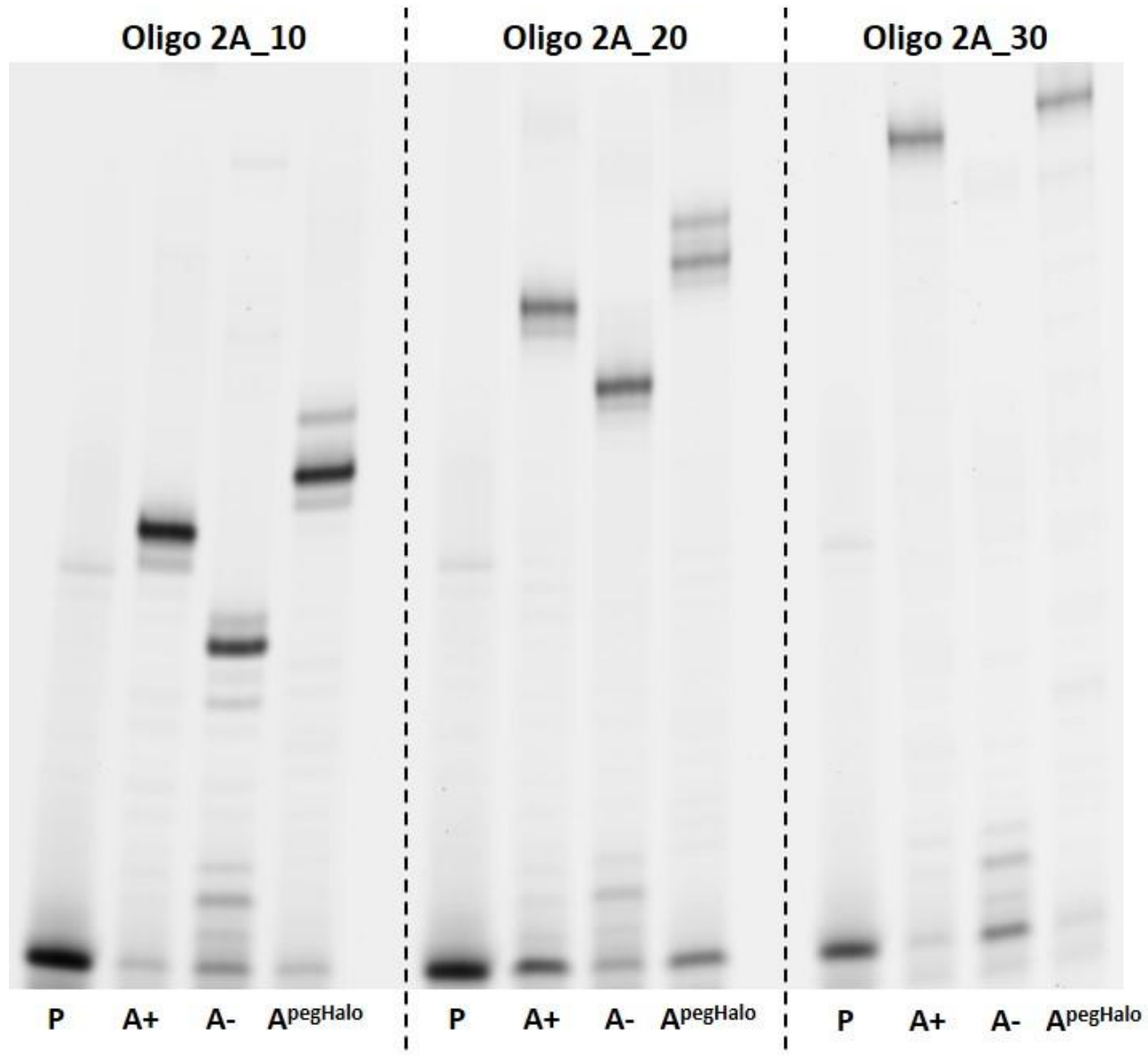

Figure S3. Denaturing PAGE analysis of PEX reactions with **dA^{pegHalo}TP** using KOD XL DNA polymerase: (P) primer **Prim248short** 5'-(Cy5)-labelled; templates **Oligo 2A_10** / **Oligo 2A_20** / **Oligo 2A_30**: (A+): dATP, dGTP, dCTP, dTTP; (A-): dGTP, dCTP, dTTP; (A^{pegHalo}): **dA^{pegHalo}TP**, dGTP, dCTP, dTTP.

2.3.2. Multiple incorporation of combination of dA^{pegHalo}TP and dC^{X}TP using 30-mer, 40-mer, 50-mer, 60-mer and 70-mer templates – Analytical scale

The reaction mixture (20 µL) contained 5'-(Cy5)-labelled **Prim248short** (3 µM, 1 µL), one of templates **T30-10** / **T40-20** / **T50-30** / **T60-40** / **T70-50** (3 µM, 1.5 µL), KOD XL DNA polymerase (0.025 U/µL, 1 µL), natural dGTP and dTTP (4 mM, 1 µL), combination of either natural dATP (2 mM, 0.5 µL) and natural dCTP (4 mM, 1 µL), or modified **dA^{pegHalo}TP** (2 mM, 0.5 µL) and one of modified **dC^{X}TP** (X = Bio, pegBio; 4 mM, 1 µL), and enzyme reaction buffer (10X, 2 µL) as supplied by the manufacturer. The reaction mixture was incubated for 30 min at 60 °C in a thermal cycler, stopped by addition of PAGE stop solution (20 µL) and heated for 5 min at 95 °C. Samples were separated by use of 12.5% denaturing PAGE and visualized using fluorescence imaging (Figure S4).

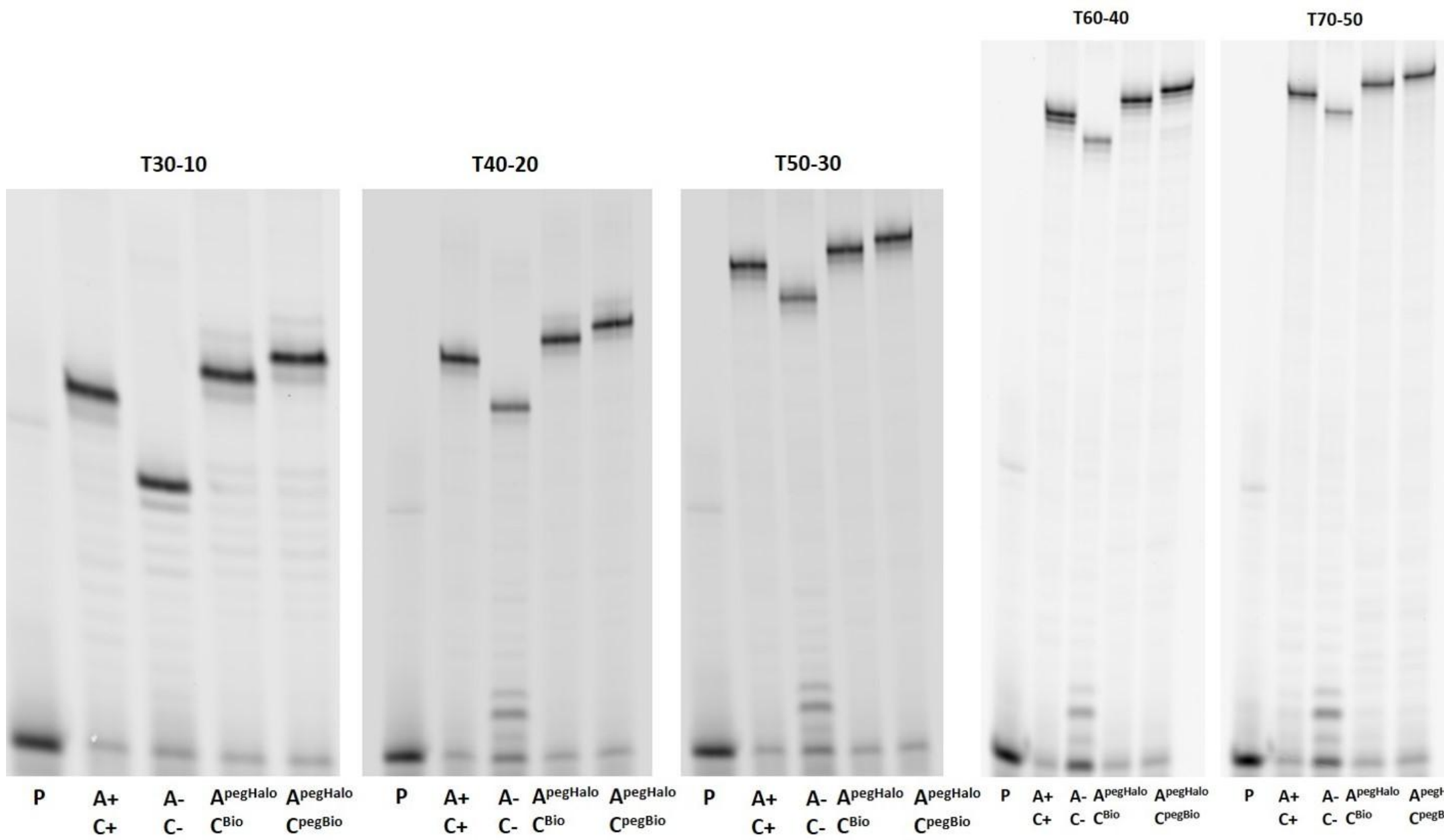

Figure S4. Denaturing PAGE analysis of PEX reactions with combination of **dA^{pegHalo}TP** and **dC^{X}TP** (X = Bio, pegBio) using KOD XL DNA polymerase: (P) primer **Prim248short** 5'-(Cy5)-labelled; templates **T30-10** / **T40-20** / **T50-30** / **T60-40** / **T70-50**: (A+, C+): dATP, dGTP, dCTP, dTTP; (A-, C-): dGTP, dTTP; (A^{pegHalo}, C^{Bio}): **dA^{pegHalo}TP**, **dC^{Bio}TP**, dGTP, dTTP; (A^{pegHalo}, C^{pegBio}): **dA^{pegHalo}TP**, **dC^{pegBio}TP**, dGTP, dTTP.

2.4. Single nucleotide incorporation (SNI) of dA^{pegHalo}TP and subsequent dCTP, and primer extension (PEX) using 31-mer template, followed by dC^{pegBio}TP incorporation mediated by Terminal Deoxynucleotidyl Transferase (TdT)

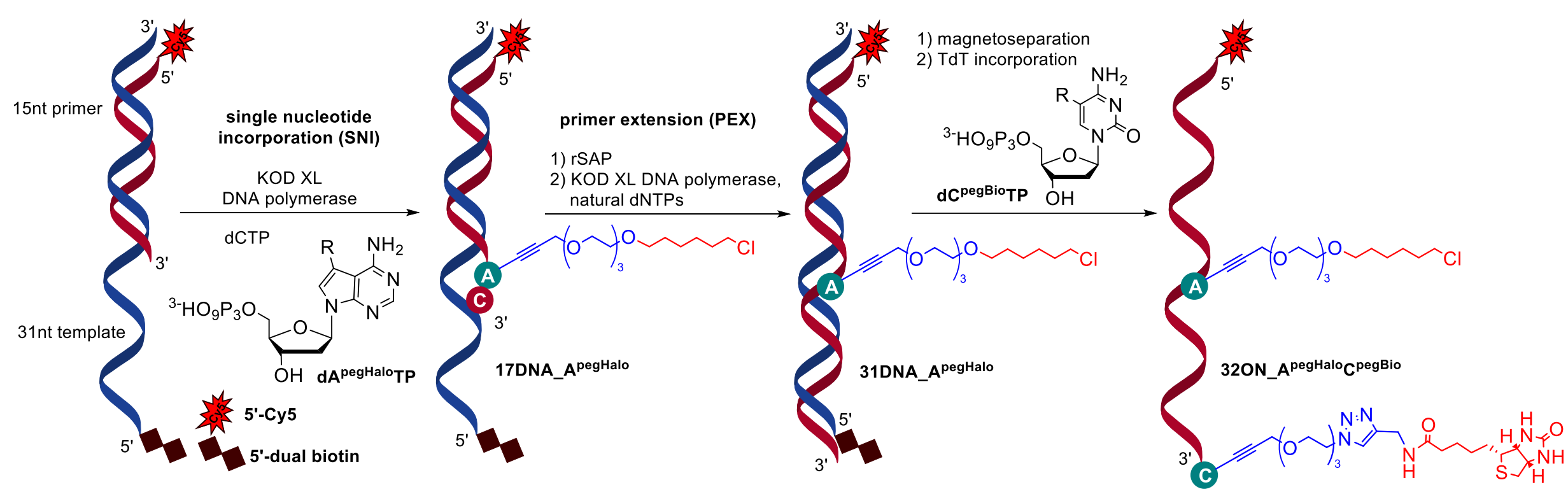

Scheme S5. Scheme of enzymatic synthesis of **31DNA_A^{pegHalo}** by sequential SNI and PEX reactions, followed by magnetoseparation to generate ssDNA, and TdT mediated incorporation of **dC^{pegBio}TP** for synthesis of **32ON_A^{pegHalo}C^{pegBio}**.

The reaction mixture (20 µL) contained 5'-(Cy5)-labelled **Prim248short** (3 µM, 1 µL), 5'-dual biotinylated template **Prb4basII-bio** (3 µM, 1.5 µL), KOD XL DNA polymerase reaction buffer (10X, 2 µL), as supplied by the manufacturer, and water. The reaction mixture was heated for 5 min at 95 °C, and allowed to cool gradually to room temperature for 10 min. Then, KOD XL DNA polymerase (0.025 U/µL, 1 µL), either natural dATP or modified **dA^{pegHalo}TP** (0.25 mM, 0.6 µL), and natural dCTP (0.25 mM, 0.6 µL) were added, and the reaction mixture was incubated for 30

min at 60 °C in a thermal cycler. Subsequently, rSAP (1 U/µL, 2 µL) was added, and the reaction mixture was incubated for 30 min at 37 °C, followed by incubation at 65 °C in a thermal cycler for further 20 min (for rSAP deactivation). Then, a second aliquot of KOD XL DNA polymerase (0.025 U/µL, 1 µL) and mixture of natural dNTPs (dATP, dCTP, dGTP, dTTP; 4 mM, 1 µL) were added, and the reaction mixture was incubated for 30 min at 60 °C in a thermal cycler. The complete reaction procedure was repeated ten times on the same scale. Then, the reaction mixtures were pooled into a single sample, an aliquot (5 µL) was taken, mixed with PAGE stop solution (5 µL), and heated for 5 min at 95 °C. Samples were separated by use of 20% denaturing PAGE and visualized using fluorescence imaging (Figure S5).

The biotinylated PEX product was purified using magnetoseparation procedure (Paragraph 2.8.1.) and analyzed by LC-ESI-MS measurements (Table S3; copies of spectra in Part 3.).

Subsequently, the reaction mixture (20 µL) containing purified PEX product 5'-(Cy5)-labelled **31ON_A^{pegHalo}** (5 pmol), **dC^{pegBio}TP** (50 µM, 1 µL), $CoCl_2$ (2.5 mM, 2 µL), Terminal Deoxynucleotidyl Transferase (TdT, 20 U/µL, 1 µL) and enzyme reaction buffer (10X, 2 µL), as supplied by the manufacturer, was incubated for 2 hours at 37 °C in a thermal cycler. The reaction was repeated ten times on the same scale. Then, the reaction mixtures were pooled into a single sample, an aliquot (5 µL) was taken, and mixed with PAGE stop solution (5 µL). Sample was separated by use of 20% denaturing PAGE and visualized using fluorescence imaging (Figure S5).

The product after TdT mediated incorporation was purified using QIAQuick® Nucleotide Removal Kit using the standard procedure, and analyzed by MALDI-TOF measurements (Table S3; copies of spectra in Part 3.).

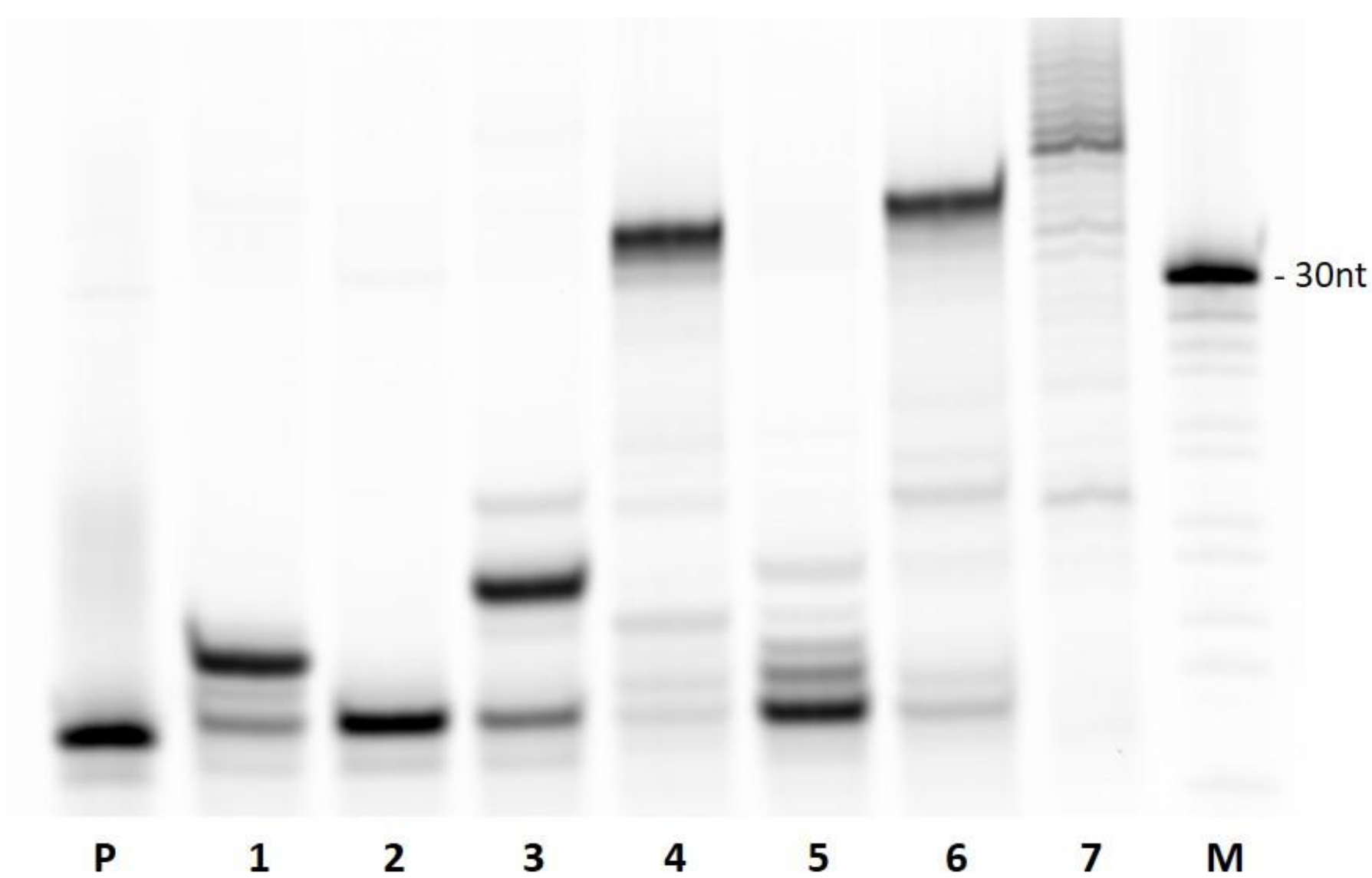

Figure S5. Denaturing PAGE analysis of SNI with **dA^{pegHalo}TP** and subsequent dCTP, and PEX reactions for complete extension using KOD XL DNA polymerase: (P) primer **Prim248short** 5'-(Cy5)-labelled; template 5'-dual biotinylated **Prb4basII-bio**: (1): SNI with dATP, dCTP; (2): no dNTPs were added; (3): SNI with **dA^{pegHalo}TP**, dCTP; (4): PEX with dATP, dCTP, dGTP, dTTP, after SNI with dATP, dCTP; (5): PEX with dCTP, dGTP, dTTP; (6): PEX with dATP, dCTP, dGTP, dTTP, after SNI with **dA^{pegHalo}TP**, dCTP. Denaturing PAGE analysis of TdT mediated incorporation of **dC^{pegBio}TP** on 5'-(Cy5)-labelled **31ON_A^{pegHalo}**: (7): 5'-(Cy5)-labelled **32ON_A^{pegHalo}C^{pegBio}** (after TdT mediated incorporation); (M): 30nt 5'-(Cy5)-labelled ON as size marker.

2.5. Procedure for semipreparative scale synthesis of dsDNA

In PEX reactions for preparation of dsDNA, one of templates **Oligo 1C** / **Oligo 1A** / **T30-10** / **Oligo 2A_10** / **T40-20** / **Oligo 2A_20** / **T50-30** / **Oligo 2A_30** / **T60-40** / **T70-50**, one of 5'-(Cy5)-labelled or 5'-(6-FAM)-labelled primers **Prim248short**, natural dGTP, dATP, dCTP and dTTP, and/or modified **dA^{pegHalo}TP** and/or **dC^{X}TP** (X = Bio, pegBio), KOD XL DNA polymerase and enzyme reaction buffer were used. In order to obtain sufficient amount of dsDNA for further analysis, PEX reactions (described above in Sections 2.2. and 2.3.) were twenty times scaled-up compared to the analytical scale procedure for the preparation of dsDNA for MALDI-TOF and LC-MS measurements (Table S3; copies of spectra in Part 3.), and for electrophoretic mobility shift assay (Section 2.9.) The products were purified using QIAQuick® Nucleotide Removal Kit using the standard procedure, and stored in Milli-Q water.

2.6. Single nucleotide incorporation (SNI) of dA^{pegHalo}TP and subsequent dCTP using 31-mer template – Semipreparative scale

The reaction mixture (50 µL) contained 5'-(Cy5)-labelled **Prim248short** (100 µM, 0.6 µL), 5'-dual biotinylated template **Prb4basII-bio** (100 µM, 0.9 µL), KOD XL DNA polymerase reaction buffer (10X, 5 µL), as supplied by the manufacturer, and water. The reaction mixture was heated for 5 min at 95 °C, and allowed to cool gradually to room temperature for 10 min. Then, KOD XL DNA polymerase (0.5 U/µL, 0.5 µL), modified **dA^{pegHalo}TP** (4 mM, 0.75 µL) and natural dCTP (4 mM, 0.75 µL) were added, and the reaction mixture was incubated for 30 min at 60 °C in a thermal cycler. Crude reaction mixture was analyzed by LC-ESI-MS measurements (Table S3; copies of spectra in Part 3.).

2.7. Preparation of ONs for MALDI-TOF analysis

2.7.1. Single incorporation of dA^{pegHalo}TP using 19-mer template – Semipreparative scale

The reaction mixture (50 µL) contained non-labelled **Prim248short** (100 µM, 0.4 µL), 5'-dual biotinylated template **Oligo 1A-bio** (100 µM, 0.45 µL), KOD XL DNA polymerase (0.5 U/µL, 0.5 µL), natural dGTP (100 mM, 0.4 µL), modified **dA^{pegHalo}TP** (50 mM, 0.8 µL) and enzyme reaction buffer (10X, 5 µL) as supplied by the manufacturer. The reaction mixture was incubated in a thermal cycler for 30 min at 60 °C and stopped by cooling down to 4 °C.
Biotinylated PEX product was purified using magnetoseparation procedure (Paragraph 2.8.1.) and analyzed by MALDI-TOF measurements (Table S3; copies of spectra in Part 3.).

2.7.2. Single incorporation of dC^{X}TP using 19-mer template – Semipreparative scale

The reaction mixture (50 µL) contained non-labelled **Prim248short** (100 µM, 0.4 µL), 5'-dual biotinylated template **Oligo 1C-bio** (100 µM, 0.45 µL), KOD XL DNA polymerase (0.5 U/µL, 0.5 µL), natural dGTP (100 mM, 0.4 µL), one of modified **dC^{X}TP** (X = Bio, pegBio; 100 mM, 0.4 µL) and enzyme reaction buffer (10X, 5 µL) as supplied by the manufacturer. The reaction

mixture was incubated in a thermal cycler for 30 min at 60 °C and stopped by cooling down to 4 °C.
Biotinylated PEX product was purified using magnetoseparation procedure (Paragraph 2.8.1.) and analyzed by MALDI-TOF measurements (Table S3; copies of spectra in Part 3.).

2.7.3. Multiple incorporation of dA^{pegHalo}TP using 30-mer, 40-mer and 50-mer templates – Semipreparative scale

The reaction mixture (50 µL) contained non-labelled **Prim248short** (100 µM, 0.4 µL), one of 5'-dual biotinylated templates **Oligo 2A_10-bio** / **Oligo 2A_20-bio** / **Oligo 2A_30-bio** (100 µM, 0.45 µL), KOD XL DNA polymerase (2.5 U/µL, 1.2 µL), natural dGTP, dCTP and dTTP (4 mM, 2.5 µL), modified **dA^{pegHalo}TP** (50 mM, 0.1 µL) and enzyme reaction buffer (10X, 5 µL) as supplied by the manufacturer. The reaction mixture was incubated in a thermal cycler for 10 min at 60 °C and stopped by cooling down to 4 °C.
Biotinylated PEX products were purified using magnetoseparation procedure (Paragraph 2.8.1.) and analyzed by MALDI-TOF measurements (Table S3; copies of spectra in Part 3.).

2.8. Procedures for ssON generation

2.8.1. ssON generation via magnetoseparation

400 µL of Streptavidin magnetic beads (SMB) were washed with binding buffer TEN100 (3×200 µL; 10 mM Tris-HCl, 1 mM EDTA, 100 mM NaCl, pH 7.5). The PEX reaction mixture (50 µL) was mixed with binding buffer TEN100 (50 µL) and prewashed SMB, and incubated for 30 min (15 °C, 1200 rpm). SMB were captured on magnet (DynaMag-2, Invitrogen), and successively washed with washing buffer TEN500 (3×200 µL; 10 mM Tris-HCl, 1 mM EDTA, 500 mM NaCl, pH 7.5) and water (4×200 µL). Modified strand was then released by denaturation in 50 µL of water (incubation for 2 min, 72 °C, 900 rpm). SMB bearing template strand were immediately captured on magnet and water solution containing modified strand was taken out, freeze-dried, then dissolved in water and analyzed by MALDI-TOF mass spectrometry.

2.8.2. ssON generation via λ-exonuclease digestion

The reaction mixture (50 µL) contained 5'-(Cy5)-labelled **Prim248short** (100 µM, 0.3 µL), 5'-phosphorylated template **T70-50 5'-P** (100 µM, 0.45 µL), KOD XL DNA polymerase (0.5 U/µL, 0.5 µL), natural dGTP and dTTP (50 mM, 0.8 µL), modified **dA^{pegHalo}TP** (25 mM, 0.4 µL), one of **dC^{X}TP** (X = Bio, pegBio; 100 mM, 0.4 µL) and enzyme reaction buffer (10X, 5 µL). The reaction mixture was incubated in a thermal cycler for 30 min at 60 °C. Then, λ-exonuclease (6-8 U) was added to the PEX reaction mixture, followed by incubation at 37 °C for 2 h. The generated ssON was purified by QIAQuick® Nucleotide Removal Kit using the standard procedure, and analyzed by LC-ESI-MS measurements (Table S3; copies of spectra in Part 3.).

Table S3. Overview of modified oligonucleotides and their masses after MALDI-TOF and LC-ESI-MS measurements

Name	Mass calculated [Da]	Mass found [Da]	Δ [Da]	Figure number
**19ON_A^{pegHalo}**	6279.06	6279.39	0.3	Figure S26
**19ON_C^{Bio}**	6230.02	6232.46	2.4	Figure S27
**19ON_C^{pegBio}**	6443.13	6444.07	0.9	Figure S28
**30ON_A^{pegHalo}**	9993.42	9993.63	0.2	Figure S29
**40ON_A^{pegHalo}**	13065.42	13067.15	1.7	Figure S30
**50ON_A^{pegHalo}**	16122.32	16123.41	1.1	Figure S31
**30DNA_A^{pegHalo}C^{Bio}** [a]	10598.18	10598.61	0.4	Figure S32
**30DNA_A^{pegHalo}C^{pegBio}** [a]	10811.29	10816.56	5.3	Figure S33
**40DNA_A^{pegHalo}C^{Bio}** [a]	13792.08	13788.95	3.1	Figure S34
**40DNA_A^{pegHalo}C^{pegBio}** [a]	14005.19	14011.86	6.7	Figure S35
**50DNA_A^{pegHalo}C^{Bio}** [a]	17009.18	17013.35	4.2	Figure S36
**50DNA_A^{pegHalo}C^{pegBio}** [a]	17222.29	17220.96	1.3	Figure S37
**60DNA_A^{pegHalo}C^{Bio}** [a]	20200.08	20192.65	7.4	Figure S38
**60DNA_A^{pegHalo}C^{pegBio}** [a]	20413.19	20414.75	1.6	Figure S39
**70ON_A^{pegHalo}C^{Bio}** [a] *	23392.08	23385.00	7.1	Figure S40B
**70ON_A^{pegHalo}C^{pegBio}** [a] *	23605.19	23595.00	10.2	Figure S41B
**17ON_A^{pegHalo}** [a] *	6114.26	6112.00	2	Figure S42B
**31ON_A^{pegHalo}** [a] *	10455.06	10450.00	5	Figure S43B
**32ON_A^{pegHalo}C^{pegBio}** [a]	11236.49	11238.92	2.4	Figure S44

[a] 5'-(Cy5)-labelled; ON – single stranded DNA; DNA – double stranded DNA. * Analyzed by LC-ESI-MS measurement.

2.9. Electrophoretic mobility shift assays (EMSA)

2.9.1. EMSA of natural and modified dsDNA (19-mer) with recombinant GST-Halotag protein

The reaction mixture (10 µL) containing 5'-(Cy5)-labelled **19DNA_A^{X}** (X = Natural, pegHalo; 0.5 µM, 1 equiv.; prepared as described above in Section 2.5.) in PBS1 buffer was incubated with GST-Halotag protein (1 µM, 2 equiv.) at 37 °C for 17 h in a thermal cycler (with heated lid at 65 °C). After this time, the samples were combined with 10 µL of VPS loading buffer, heated at 95 °C for 5 min, separated by 10% SDS PAGE and visualized using fluorescence imaging (Figure S6).

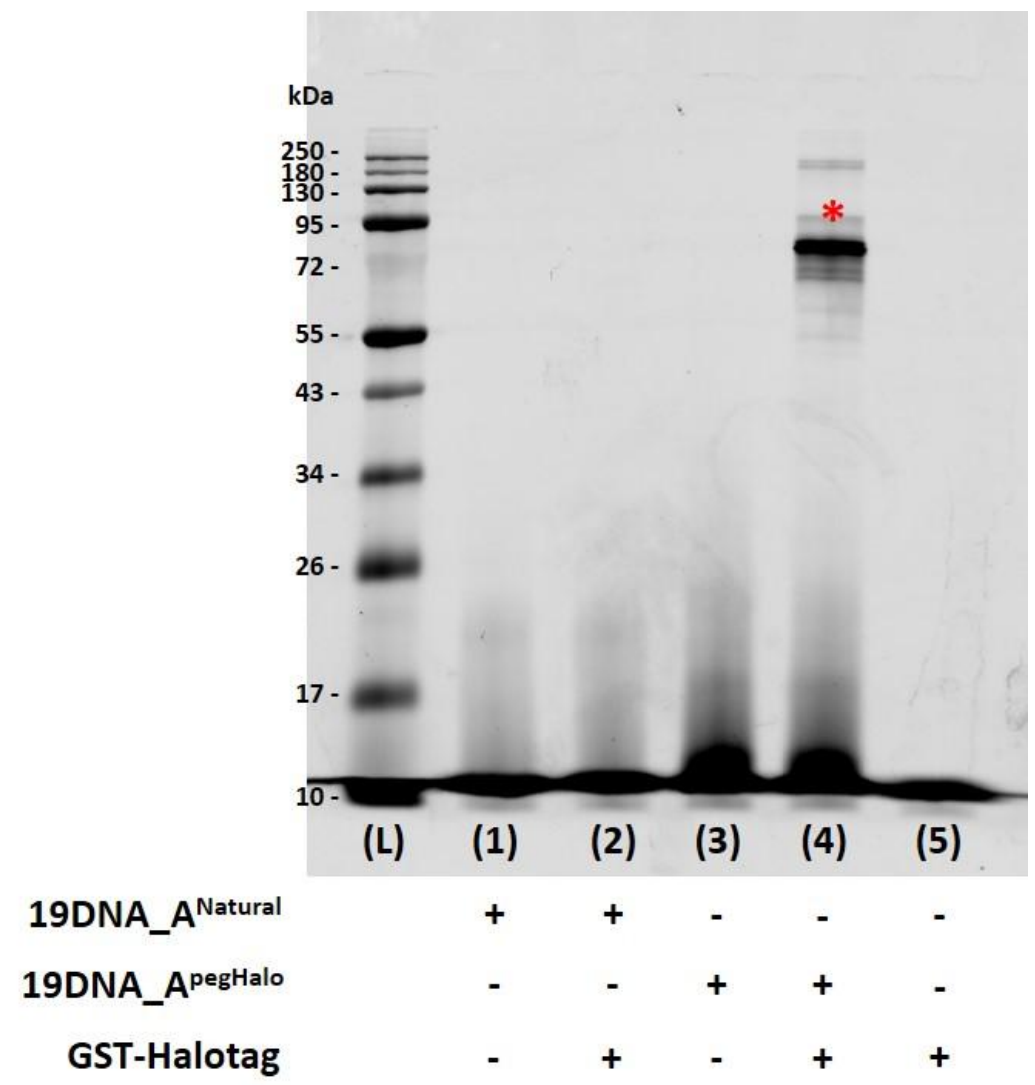

Figure S6. EMSA analysis of 5'-(Cy5)-labelled **19DNA_A^{X}** (X = Natural, pegHalo) with GST-Halotag protein. Cy5 channel scan of 10% SDS PAGE. (L) ladder, (*) DNA-protein conjugate.

2.9.2. EMSA of natural and modified dsDNA (19-mer) with recombinant mSA protein

The reaction mixture (10 µL) containing 5'-(Cy5)-labelled **19DNA_C^{X}** (X = Natural, Bio, pegBio; 0.5 µM, 1 equiv.; prepared as described above in Section 2.5.) in PBS2 buffer was incubated with mSA protein (1 µM, 2 equiv.) at 37 °C for 17 h in a thermal cycler (with heated lid at 65 °C). After this time, the samples were combined with 2 µL of sterile glycerol, separated by 10% protein native PAGE and visualized using fluorescence imaging (Figure S7A,B).

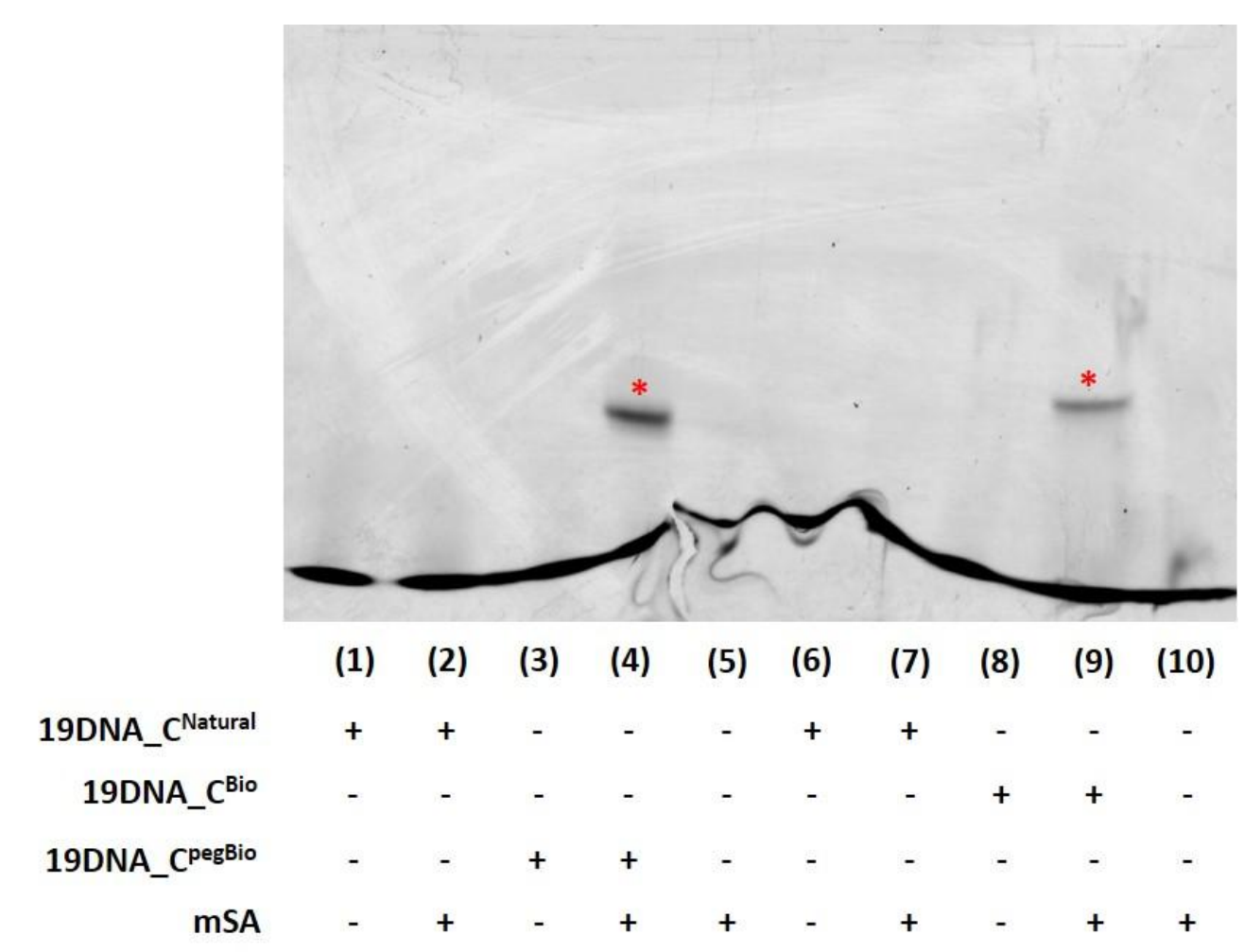

Figure S7A. EMSA analysis of 5'-(Cy5)-labelled **19DNA_C^{X}** (X = Natural, Bio, pegBio) with mSA protein. Cy5 channel scan of 10% protein native PAGE. (*) DNA-protein complex.

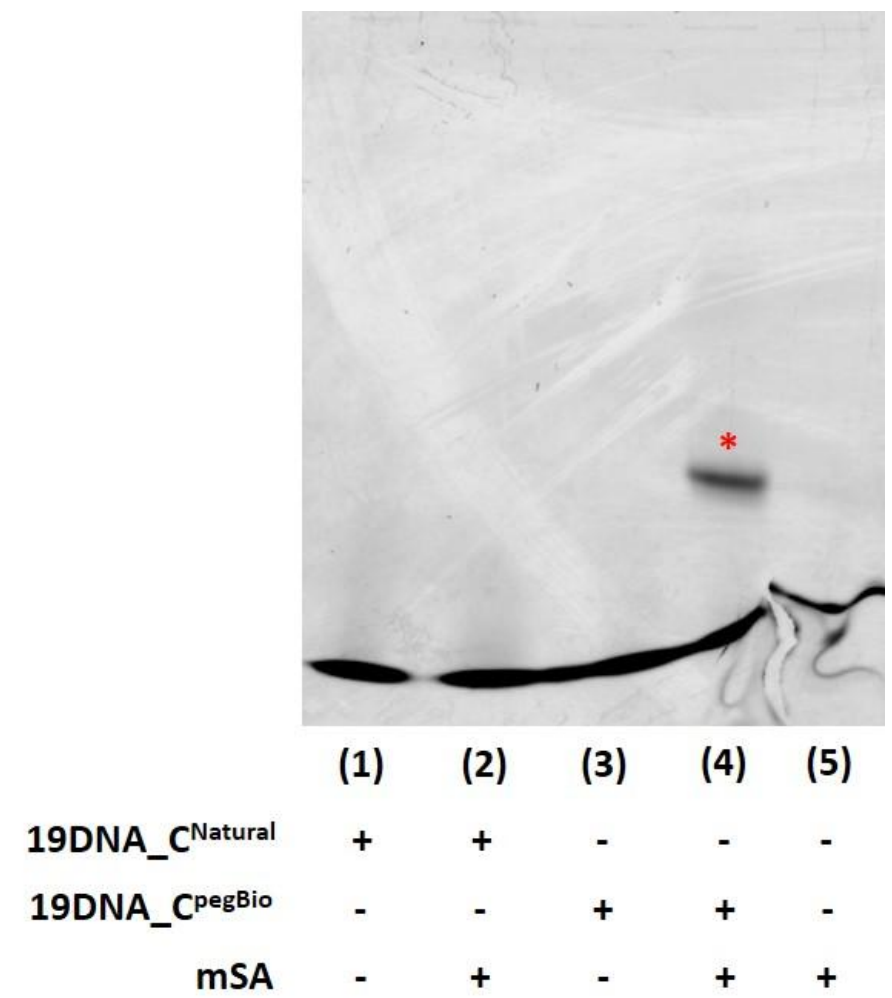

Figure S7B. EMSA analysis of selected samples 5'-(Cy5)-labelled **19DNA_C^{X}** (X = Natural, pegBio) with mSA protein. Cy5 channel scan of 10% protein native PAGE. (*) DNA-protein complex.

2.9.3. EMSA of modified dsDNA (19-mer) with recombinant GST-Halotag protein – time dependent experiment

The reaction mixture (10 µL) containing 5'-(Cy5)-labelled **19DNA_A^{pegHalo}** (0.5 µM, 1 equiv.; prepared as described above in Section 2.5.) in PBS1 buffer was incubated with GST-Halotag protein (1 µM, 2 equiv.) at 37 °C for different times (1 h, 3 h, 5 h, 8 h, 17 h) in a thermal cycler (with heated lid at 65 °C). After these times, the samples were combined with 10 µL of VPS loading buffer, heated at 95 °C for 5 min, separated by 15% SDS PAGE and visualized using fluorescence imaging (Figure S8).

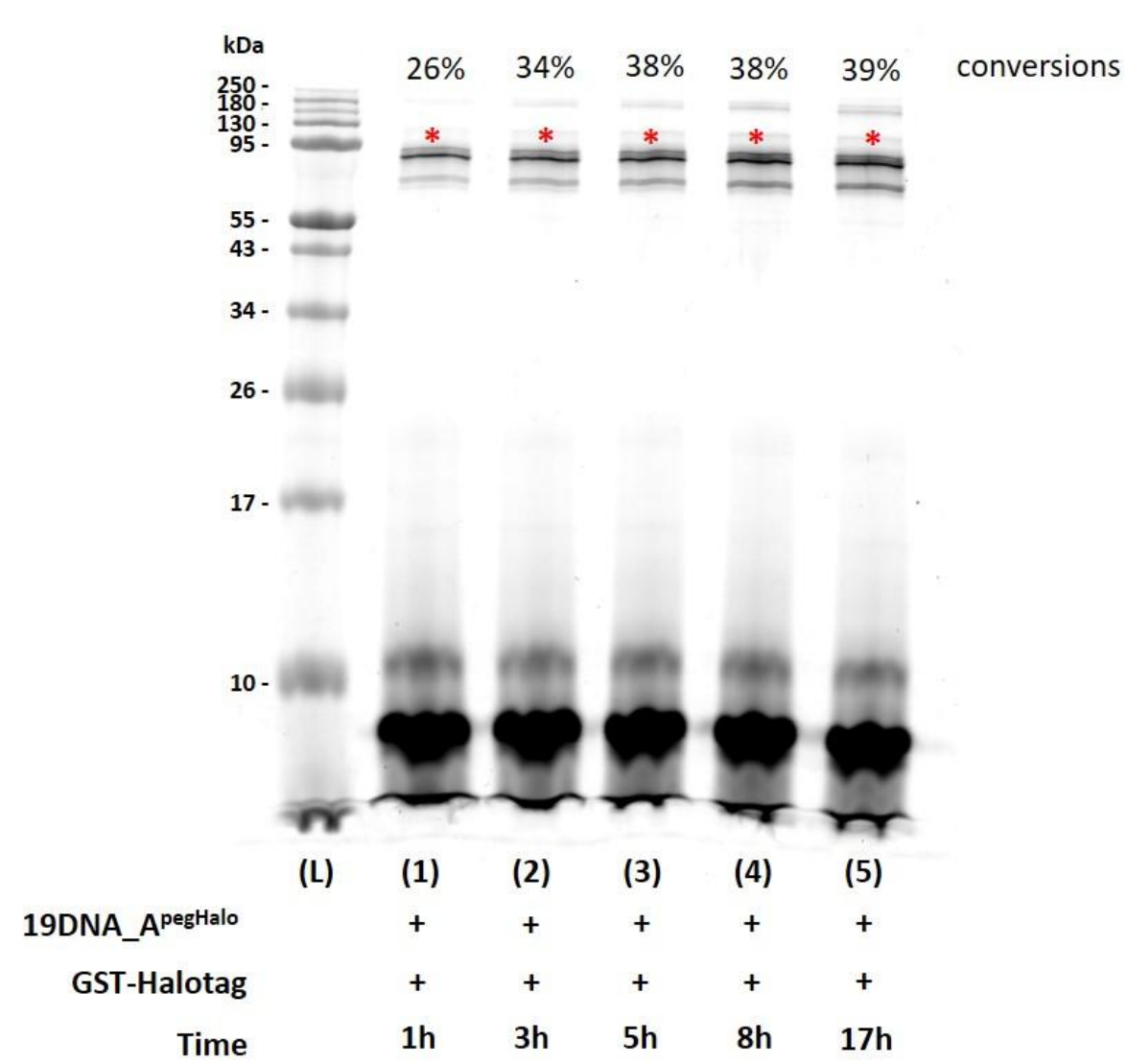

Figure S8. EMSA analysis of 5'-(Cy5)-labelled **19DNA_A^{pegHalo}** with GST-Halotag protein – time dependent experiment. Cy5 channel scan of 15% SDS PAGE. (L) ladder, (*) DNA-protein conjugate. Calculated conversions of conjugation reaction.

2.9.4. EMSA of modified dsDNA (19-mer) with recombinant mSA protein – time dependent experiment

The reaction mixture (10 µL) containing 5'-(Cy5)-labelled **19DNA_C^{X}** (X = Bio, pegBio; 0.5 µM, 1 equiv.; prepared as described above in Section 2.5.) in PBS2 buffer was incubated with mSA protein (0.5 µM, 1 equiv.) at 37 °C for different times (1 h, 3 h, 5 h, 8 h, 17 h) in a thermal cycler (with heated lid at 65 °C). After these times, the samples were combined with 2 µL of sterile glycerol, separated by 10% protein native PAGE and visualized using fluorescence imaging (Figure S9).

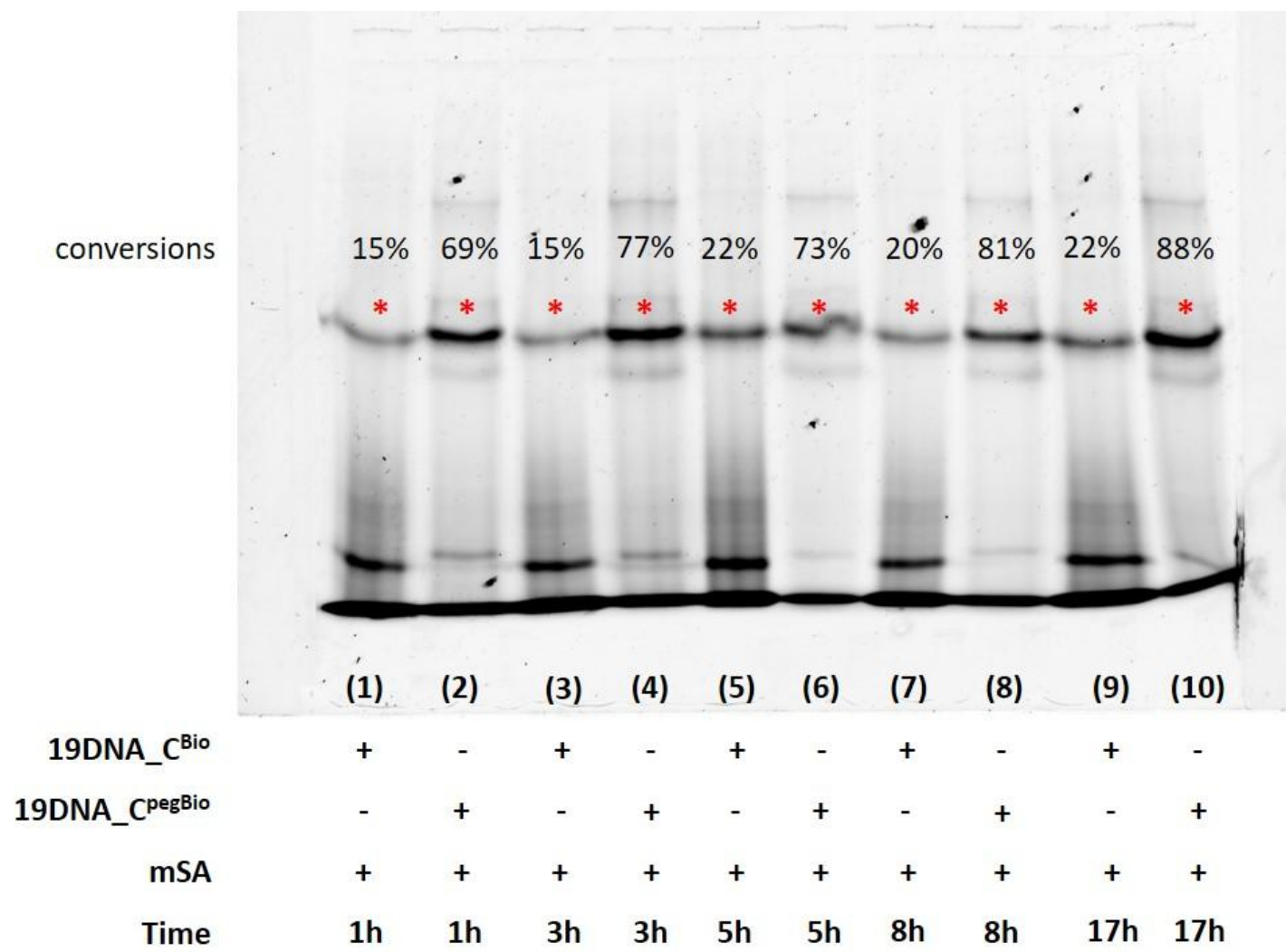

Figure S9. EMSA analysis of 5'-(Cy5)-labelled **19DNA_C^{X}** (X = Bio, pegBio) with mSA protein – time dependent experiment. Cy5 channel scan of 10% protein native PAGE. (*) DNA-protein complex. Calculated conversions of complexes formation.

2.9.5. EMSA of natural and double modified dsDNA (30-, 40- and 50-mer) with recombinant GST-Halotag protein

The reaction mixture (10 µL) containing one of 5'-(Cy5)-labelled **30DNA_A^{X}**, 5'-(Cy5)-labelled **40DNA_A^{X}** or 5'-(Cy5)-labelled **50DNA_A^{X}** (X = Natural, pegHalo; 0.5 µM, 1 equiv.; prepared as described above in Section 2.5.) in PBS1 buffer was incubated with GST-Halotag protein (2 µM, 4 equiv.) at 37 °C for 17 h in a thermal cycler (with heated lid at 65 °C). After this time, the samples were combined with 10 µL of VPS loading buffer, heated at 95 °C for 5 min, separated by 10% SDS PAGE and visualized using fluorescence imaging (Figure S10).

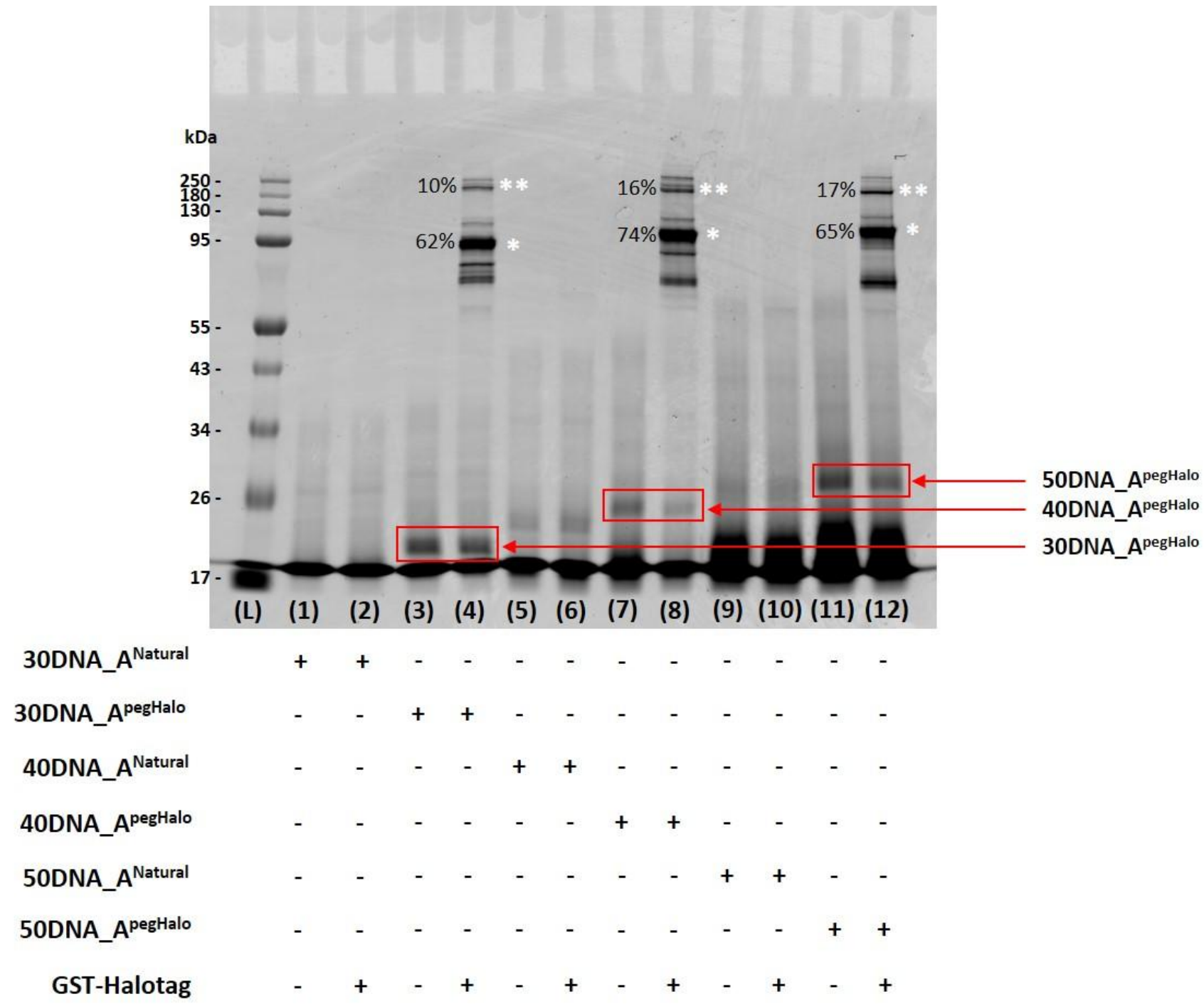

Figure S10. EMSA analysis of 5'-(Cy5)-labelled **30DNA_A^{X}**, 5'-(Cy5)-labelled **40DNA_A^{X}** and 5'-(Cy5)-labelled **50DNA_A^{X}** (X = Natural, pegHalo) with GST-Halotag protein. Cy5 channel scan of 10% SDS PAGE. (L) ladder, (*) DNA-protein mono-conjugate, (**) DNA-protein double-conjugate. Calculated conversions of conjugation reactions.

2.9.6. EMSA of double modified dsDNA (30-, 40- and 50-mer) with recombinant GST-Halotag protein – concentration dependent experiment

The reaction mixture (10 µL) containing one of 5'-(6-FAM)-labelled **30DNA_A^{pegHalo}C^{X}**, 5'-(6-FAM)-labelled **40DNA_A^{pegHalo}C^{X}** or 5'-(6-FAM)-labelled **50DNA_A^{pegHalo}C^{X}** (X = Bio, pegBio; 0.5 µM, 1 equiv.; prepared as described above in Section 2.5.) in PBS1 buffer was incubated with different concentrations of GST-Halotag protein (0.5 µM, 1 equiv.; 5 µM, 10 equiv.; 25 µM, 50 equiv.) at 37 °C for 17 h in a thermal cycler (with heated lid at 65 °C). After this time, the samples were combined with 10 µL of VPS loading buffer, heated at 95 °C for 5 min, separated by 10% SDS PAGE and visualized using fluorescence imaging (Figures S11 – S13).

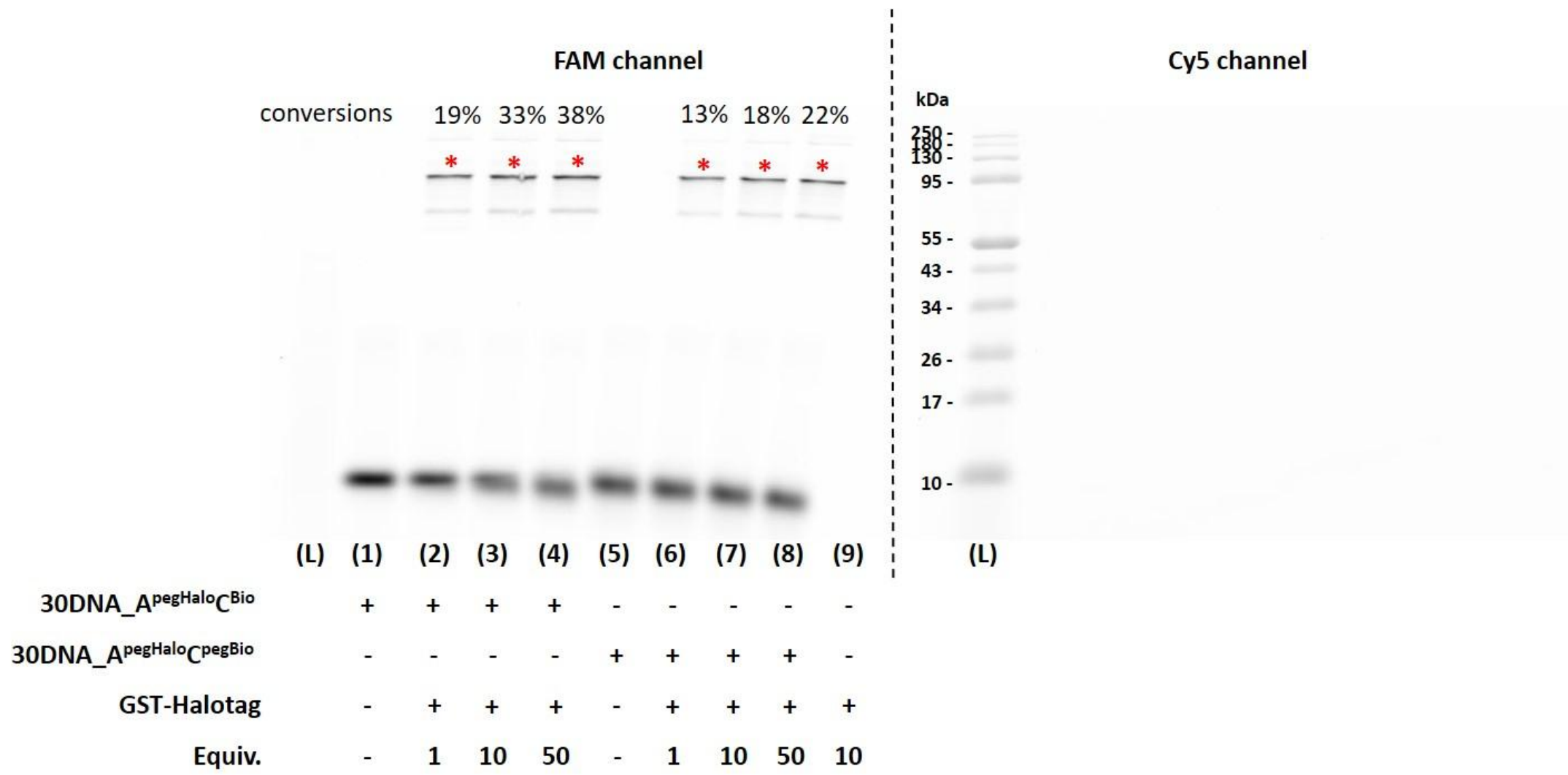

Figure S11. EMSA analysis of 5'-(6-FAM)-labelled **30DNA_A^{pegHalo}C^{X}** (X = Bio, pegBio) with GST-Halotag protein – concentration dependent experiment. On the left: FAM channel scan of 10% SDS PAGE. On the right: Cy5 channel scan of 10% SDS PAGE. (L) ladder, (*) DNA-protein conjugate. Calculated conversions of conjugation reactions. Ladder visible only in Cy5 channel scan.

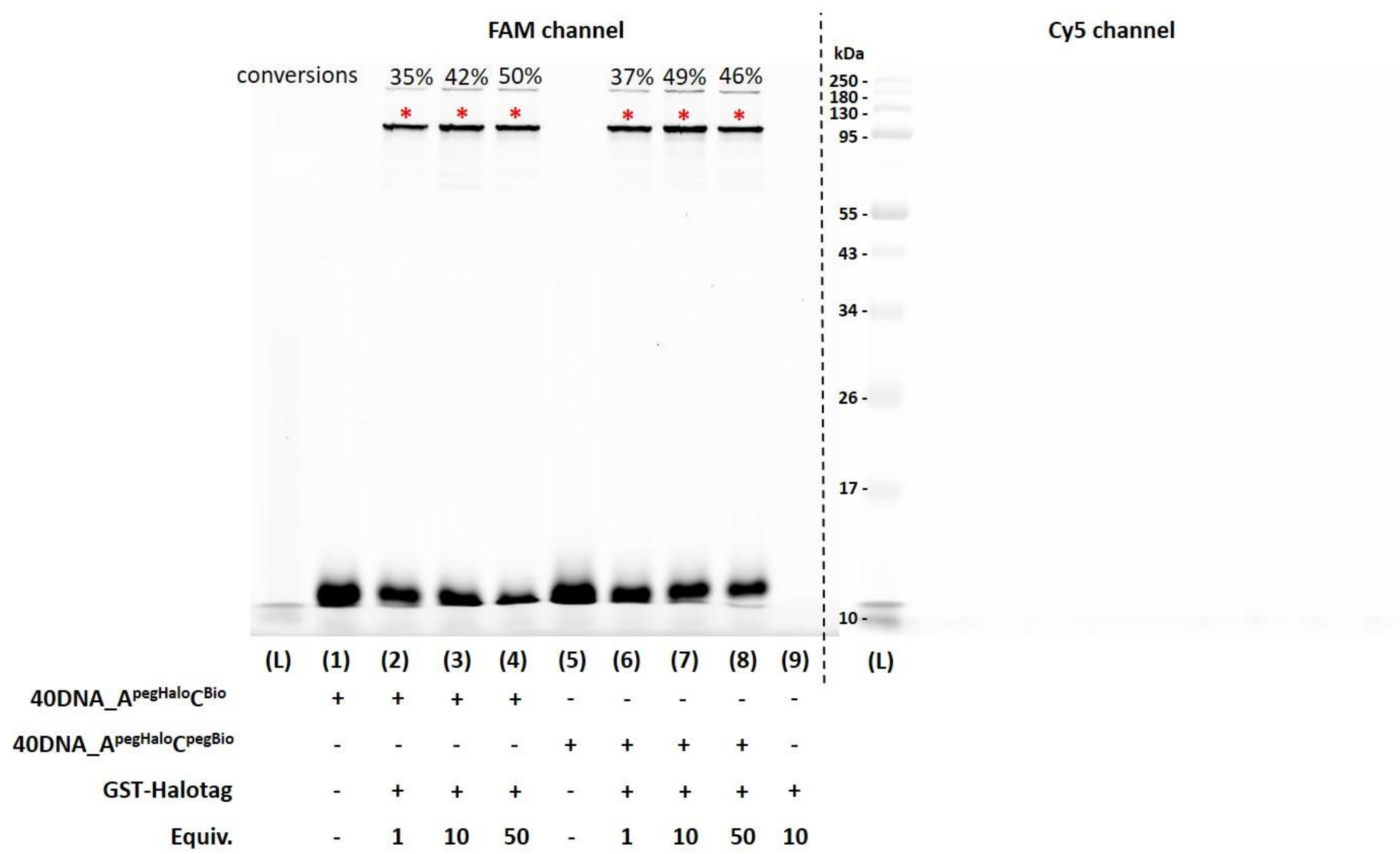

Figure S12. EMSA analysis of 5'-(6-FAM)-labelled **40DNA_A^{pegHalo}C^{X}** (X = Bio, pegBio) with GST-Halotag protein – concentration dependent experiment. On the left: FAM channel scan of 10% SDS PAGE. On the right: Cy5 channel scan of 10% SDS PAGE. (L) ladder, (*) DNA-protein conjugate. Calculated conversions of conjugation reactions. Ladder visible only in Cy5 channel scan.

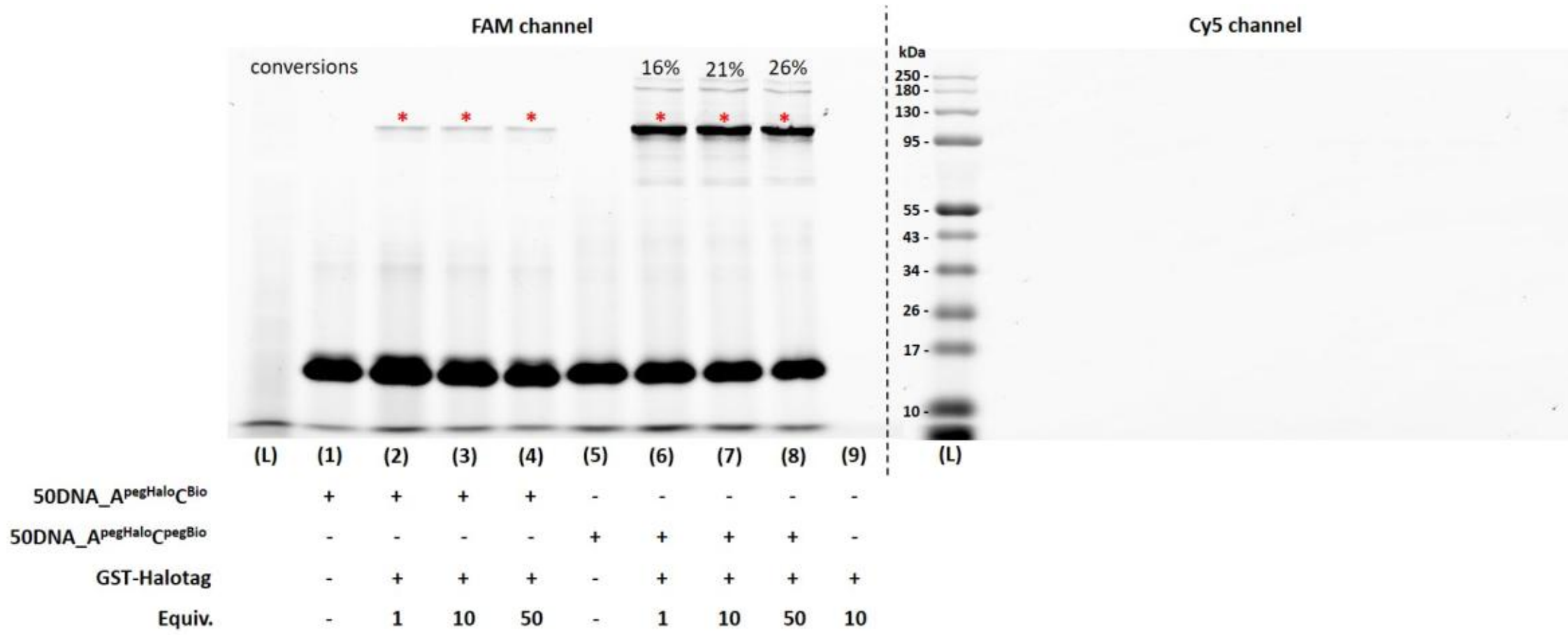

Figure S13. EMSA analysis of 5'-(6-FAM)-labelled **50DNA_A$^{\mathbf{pegHalo}}$C$^{\mathbf{X}}$** (X = Bio, pegBio) with GST-Halotag protein – concentration dependent experiment. On the left: FAM channel scan of 10% SDS PAGE. On the right: Cy5 channel scan of 10% SDS PAGE. (L) ladder, (*) DNA-protein conjugate. Calculated conversions of conjugation reactions. Ladder visible only in Cy5 channel scan.

2.9.7. EMSA of natural and double modified dsDNA (30-, 40-, 50-, 60- and 70-mer) with recombinant GST-Halotag protein – optimized reaction conditions

The reaction mixture (10 µL) containing one of 5'-(6-FAM)-labelled **30DNA_A$^{\mathbf{X}}$C$^{\mathbf{Y}}$**, 5'-(6-FAM)-labelled **40DNA_A$^{\mathbf{X}}$C$^{\mathbf{Y}}$**, 5'-(6-FAM)-labelled **50DNA_A$^{\mathbf{X}}$C$^{\mathbf{Y}}$**, 5'-(6-FAM)-labelled **60DNA_A$^{\mathbf{X}}$C$^{\mathbf{Y}}$** or 5'-(6-FAM)-labelled **70DNA_A$^{\mathbf{X}}$C$^{\mathbf{Y}}$** (X = Natural, pegHalo; Y = Natural, Bio, pegBio; 0.5 µM, 1 equiv.; prepared as described above in Section 2.5.) in PBS1 buffer was incubated with GST-Halotag protein (25 µM, 50 equiv.) at 37 °C for 17 h in a thermal cycler (with heated lid at 65 °C). After this time, the samples were combined with 10 µL of VPS loading buffer, heated at 95 °C for 5 min, separated by 10% or 12% SDS PAGE and visualized using fluorescence imaging (Figures S14A,B – S15).

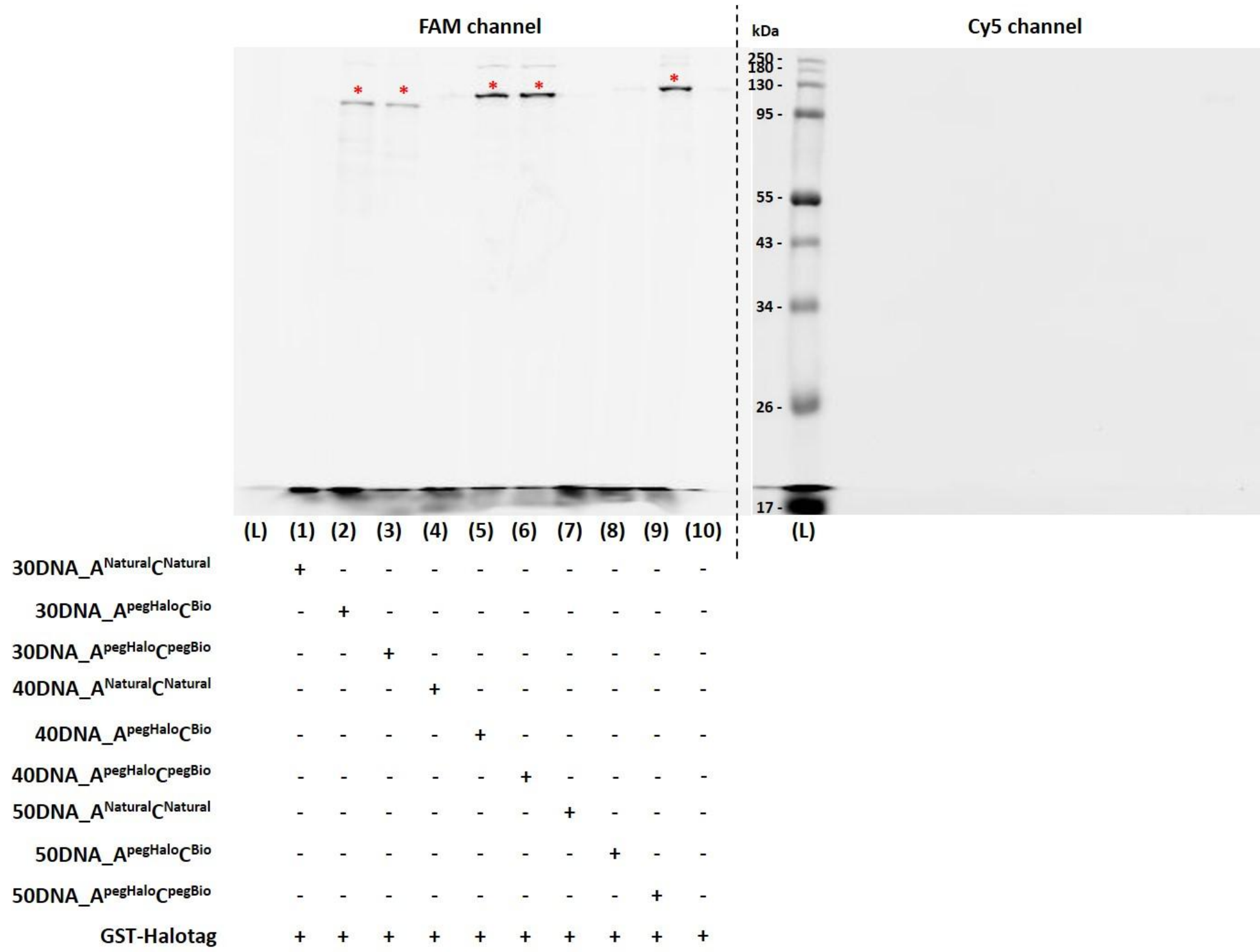

Figure S14A. EMSA analysis of 5'-(6-FAM)-labelled **30DNA_A^{X}C^{Y}**, 5'-(6-FAM)-labelled **40DNA_A^{X}C^{Y}** and 5'-(6-FAM)-labelled **50DNA_A^{X}C^{Y}** (X = Natural, pegHalo; Y = Natural, Bio, pegBio) with GST-Halotag protein. On the left: FAM channel scan of 10% SDS PAGE. On the right: Cy5 channel scan of 10% SDS PAGE. (L) ladder, (*) DNA-protein conjugate. Ladder visible only in Cy5 channel scan.

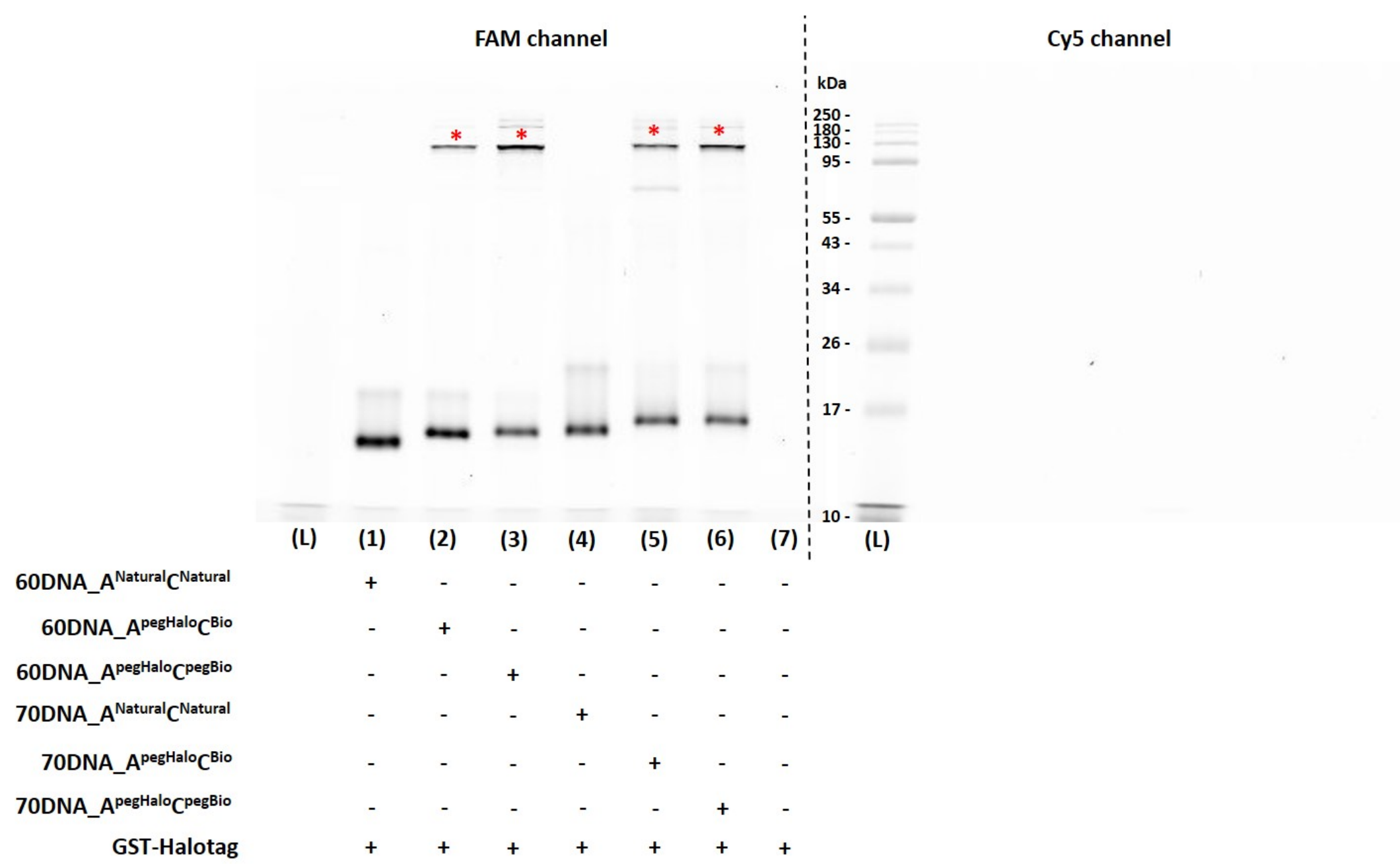

Figure S14B. EMSA analysis of 5'-(6-FAM)-labelled **60DNA_A^{X}C^{Y}** and 5'-(6-FAM)-labelled **70DNA_A^{X}C^{Y}** (X = Natural, pegHalo; Y = Natural, Bio, pegBio) with GST-Halotag protein. On the left: FAM channel scan of 10% SDS PAGE. On the right: Cy5 channel scan of 10% SDS PAGE. (L) ladder, (*) DNA-protein conjugate. Ladder visible only in Cy5 channel scan.

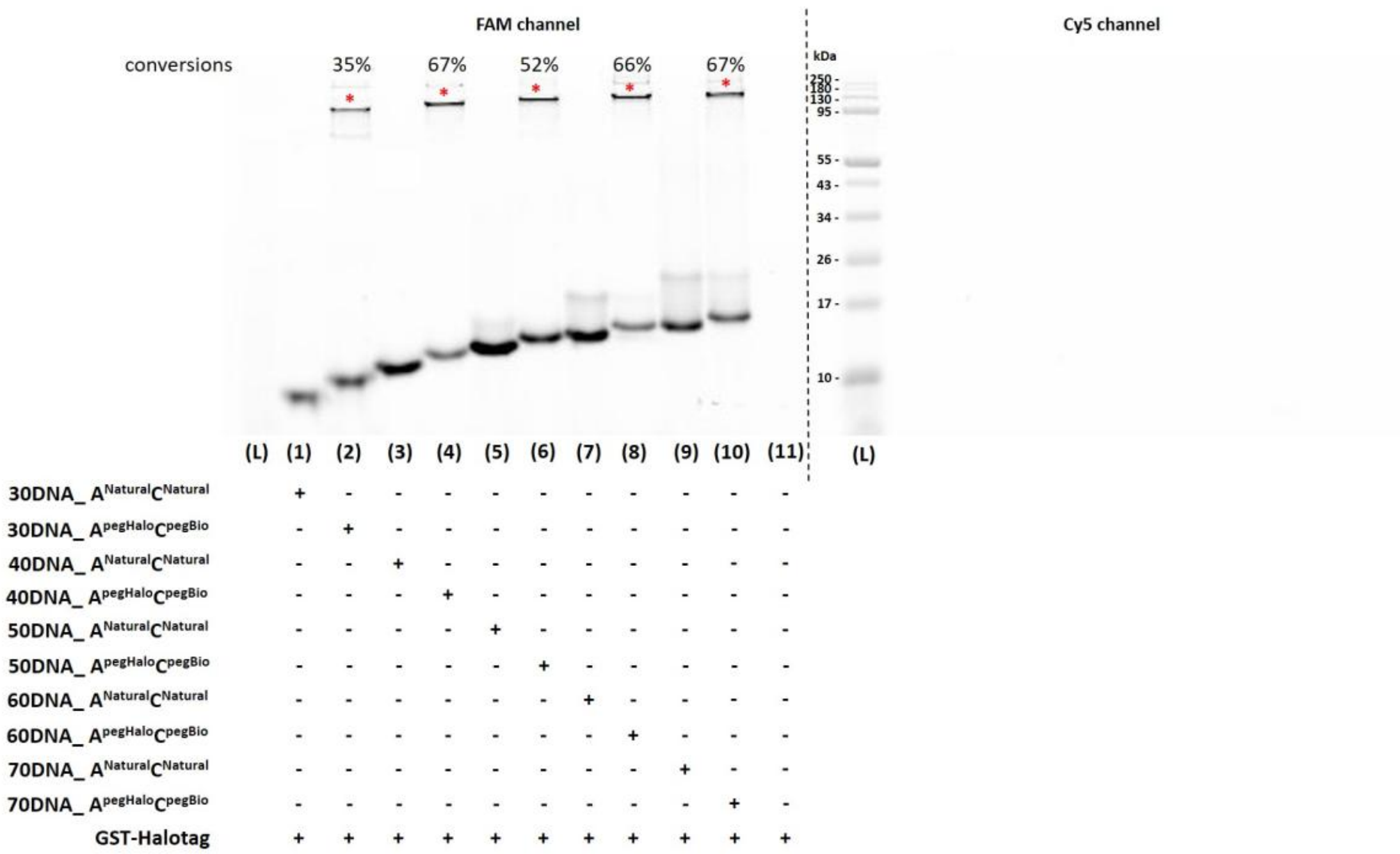

Figure S15. EMSA analysis of selected samples 5'-(6-FAM)-labelled **30DNA_A^{X}C^{Y}**, 5'-(6-FAM)-labelled **40DNA**_A^{X}C^{Y}, 5'-(6-FAM)-labelled **50DNA_A^{X}C^{Y}**, 5'-(6-FAM)-labelled **60DNA_A^{X}C^{Y}** and 5'-(6-FAM)-labelled **70DNA_A^{X}C^{Y}** (X = Natural, pegHalo; Y = Natural, pegBio) with GST-Halotag protein. On the left: FAM channel scan of 12% SDS PAGE. On the right: Cy5 channel scan of 12% SDS PAGE. (L) ladder, (*) DNA-protein conjugate. Calculated conversions of conjugation reactions. Ladder visible only in Cy5 channel scan.

2.9.8. EMSA of double modified dsDNA (30-, 40- and 50-mer) with recombinant mSA protein – concentration dependent experiment

The reaction mixture (10 µL) containing one of 5'-(Cy5)-labelled **30DNA_A^{pegHalo}C^{X}**, 5'-(Cy5)-labelled **40DNA_A^{pegHalo}C^{X}** or 5'-(Cy5)-labelled **50DNA_A^{pegHalo}C^{X}** (X = Bio, pegBio; 0.5 µM, 1 equiv.; prepared as described above in Section 2.5.) in PBS2 buffer was incubated with different concentrations of mSA protein (0.5 µM, 1 equiv.; 5 µM, 10 equiv.; 50 µM, 100 equiv.; 250 µM, 500 equiv.) at 37 °C for 17 h in a thermal cycler (with heated lid at 65 °C). After this time, the samples were combined with 2 µL of sterile glycerol, separated by 15% protein native PAGE and visualized using fluorescence imaging (Figures S16 – S18).

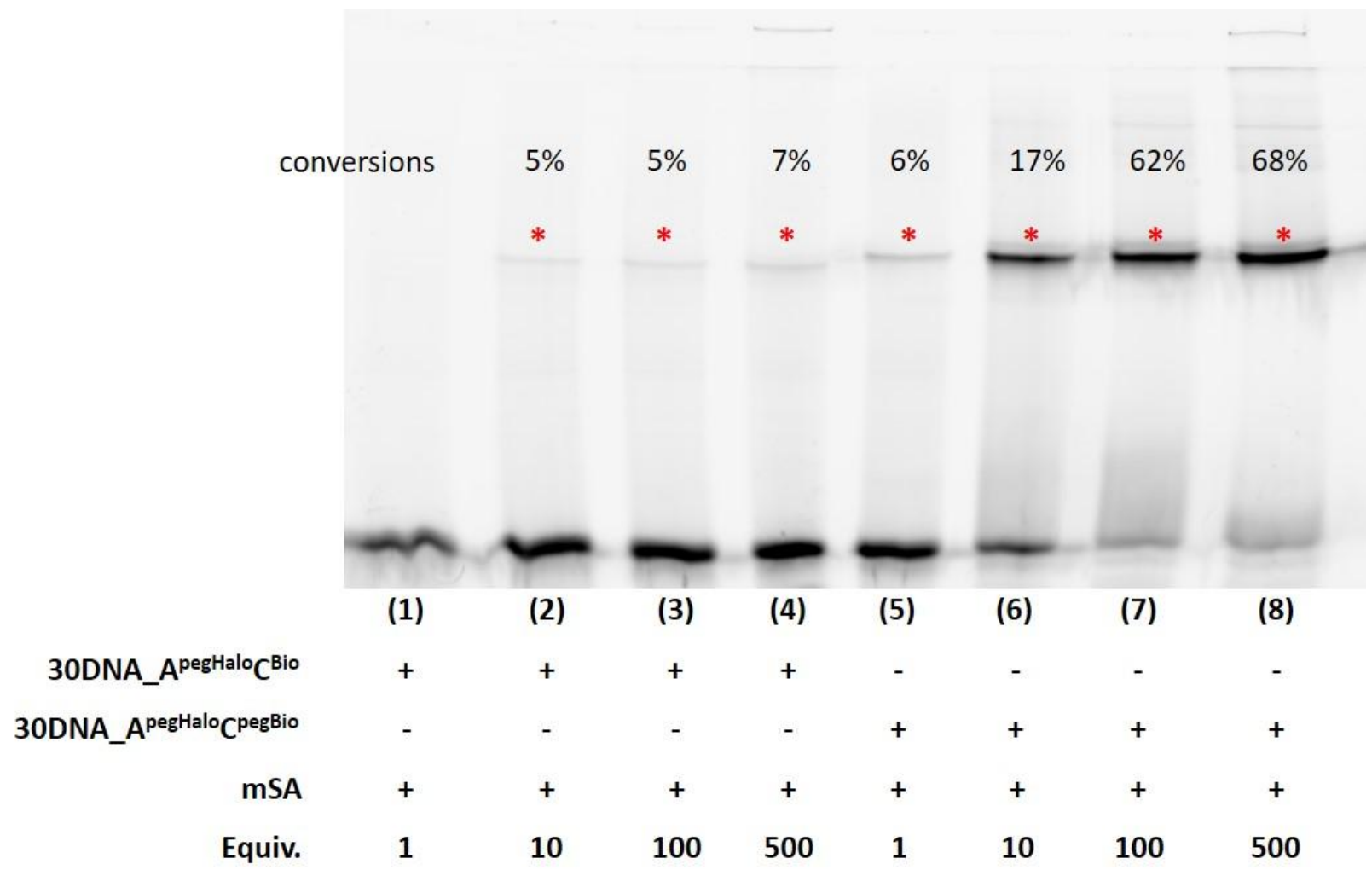

Figure S16. EMSA analysis of 5'-(Cy5)-labelled **30DNA_A^{pegHalo}C^{X}** (X = Bio, pegBio) with mSA protein – concentration dependent experiment. Cy5 channel scan of 15% protein native PAGE. (*) DNA-protein complex. Calculated conversions of complexes formation.

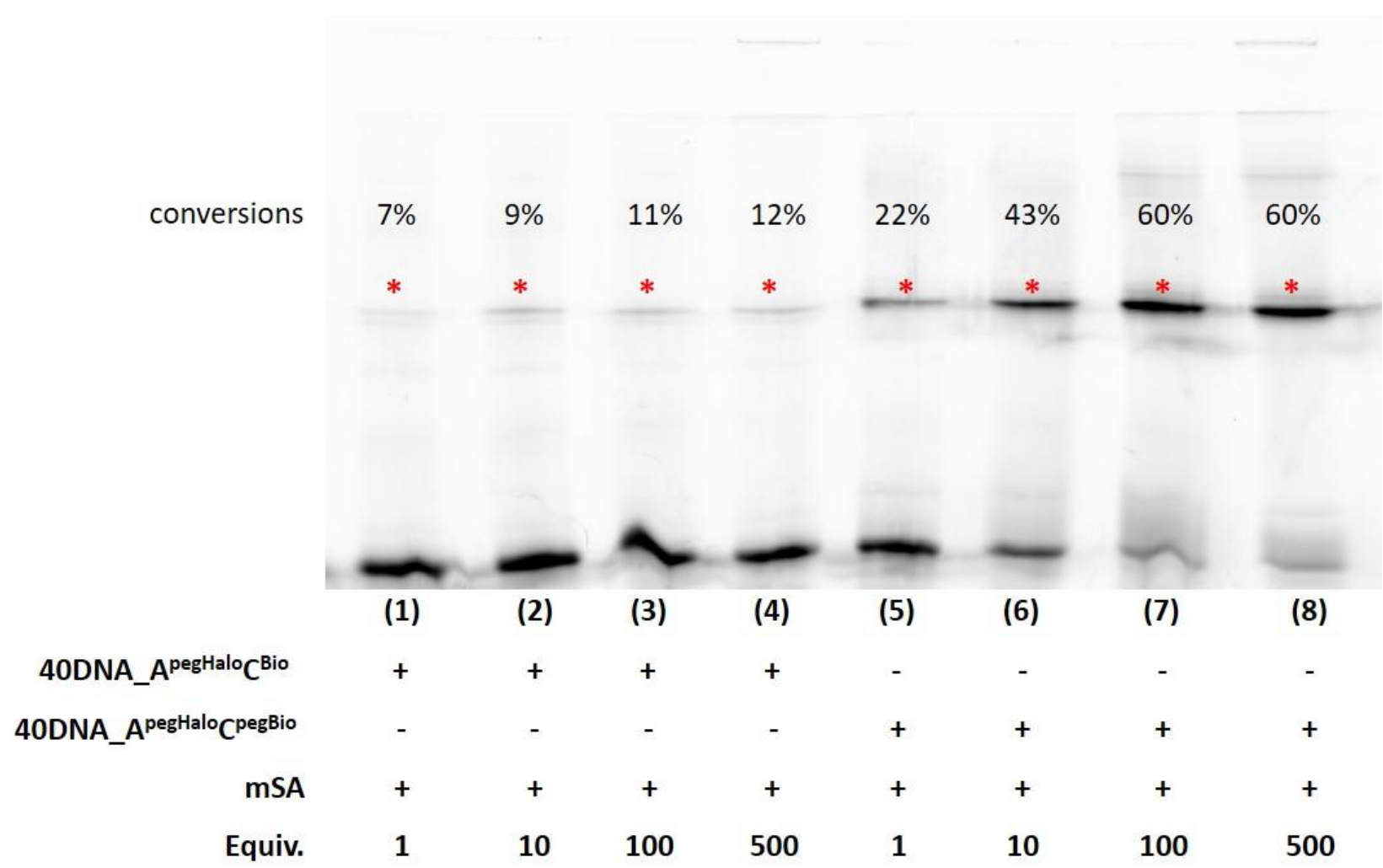

Figure S17. EMSA analysis of 5'-(Cy5)-labelled **40DNA_A^{pegHalo}C^{X}** (X = Bio, pegBio) with mSA protein – concentration dependent experiment. Cy5 channel scan of 15% protein native PAGE. (*) DNA-protein complex. Calculated conversions of complexes formation.

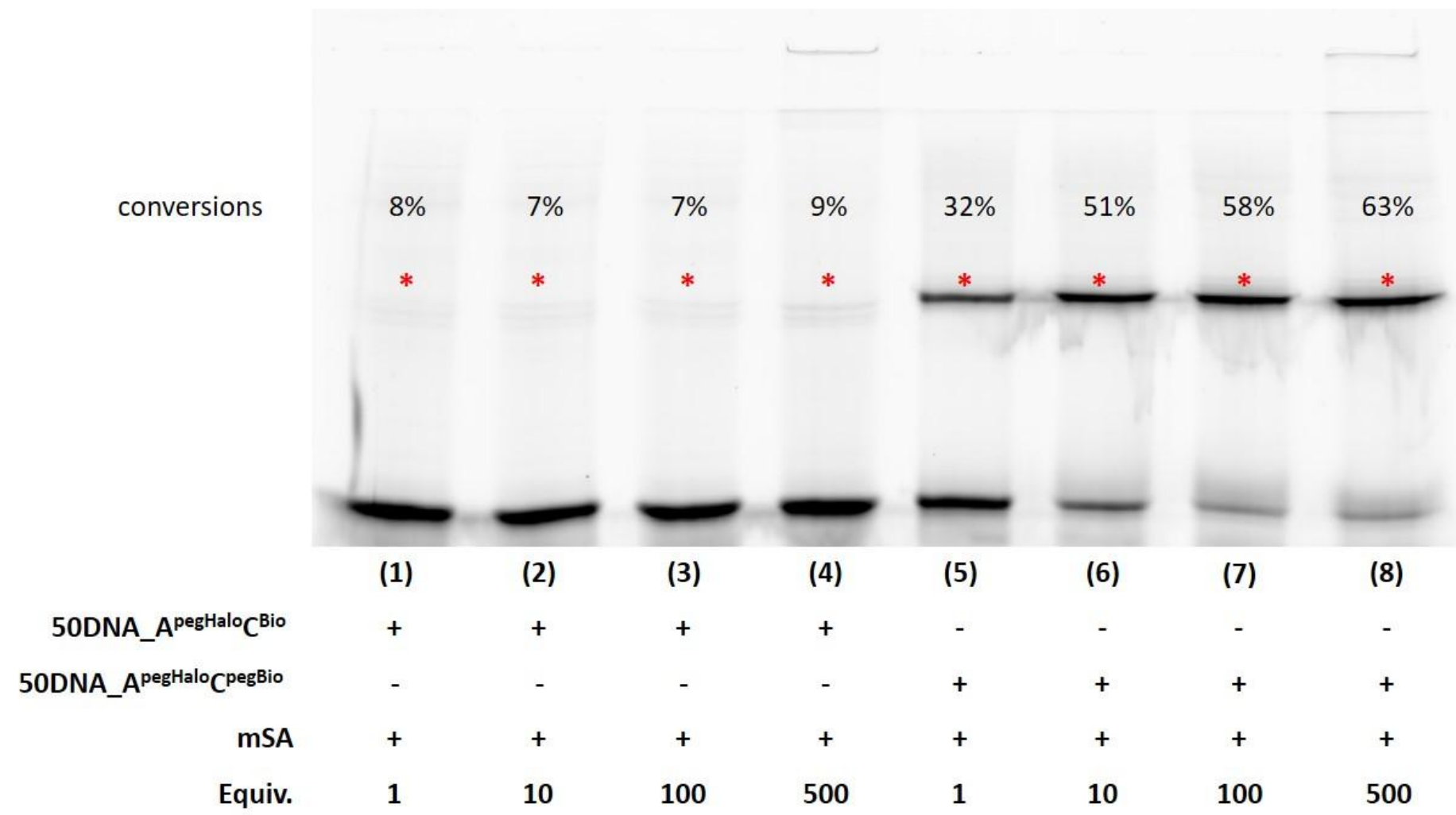

Figure S18. EMSA analysis of 5’-(Cy5)-labelled **50DNA_A$^{\mathbf{pegHalo}}$C$^{\mathbf{X}}$** (X = Bio, pegBio) with mSA protein – concentration dependent experiment. Cy5 channel scan of 15% protein native PAGE. (*) DNA-protein complex. Calculated conversions of complexes formation.

2.9.9. EMSA of natural and double modified dsDNA (30-, 40-, 50-, 60- and 70-mer) with recombinant mSA protein – optimized reaction conditions

The reaction mixture (10 µL) containing one of 5’-(6-FAM)-labelled **30DNA_A$^{\mathbf{X}}$C$^{\mathbf{Y}}$**, 5’-(6-FAM)-labelled **40DNA_A$^{\mathbf{X}}$C$^{\mathbf{Y}}$**, 5’-(6-FAM)-labelled **50DNA_A$^{\mathbf{X}}$C$^{\mathbf{Y}}$**, 5’-(6-FAM)-labelled **60DNA_A$^{\mathbf{X}}$C$^{\mathbf{Y}}$** or 5’-(6-FAM)-labelled **70DNA_A$^{\mathbf{X}}$C$^{\mathbf{Y}}$** (X = Natural, pegHalo; Y = Natural, Bio, pegBio; 0.5 µM, 1 equiv.; prepared as described above in Section 2.5.) in PBS2 buffer was incubated with mSA protein (50 µM, 100 equiv.) at 37 °C for 17 h in a thermal cycler (with heated lid at 65 °C). After this time, the samples were combined with 2 µL of sterile glycerol, separated by 12% or 15% protein native PAGE and visualized using fluorescence imaging (Figures S19A,B – S20).

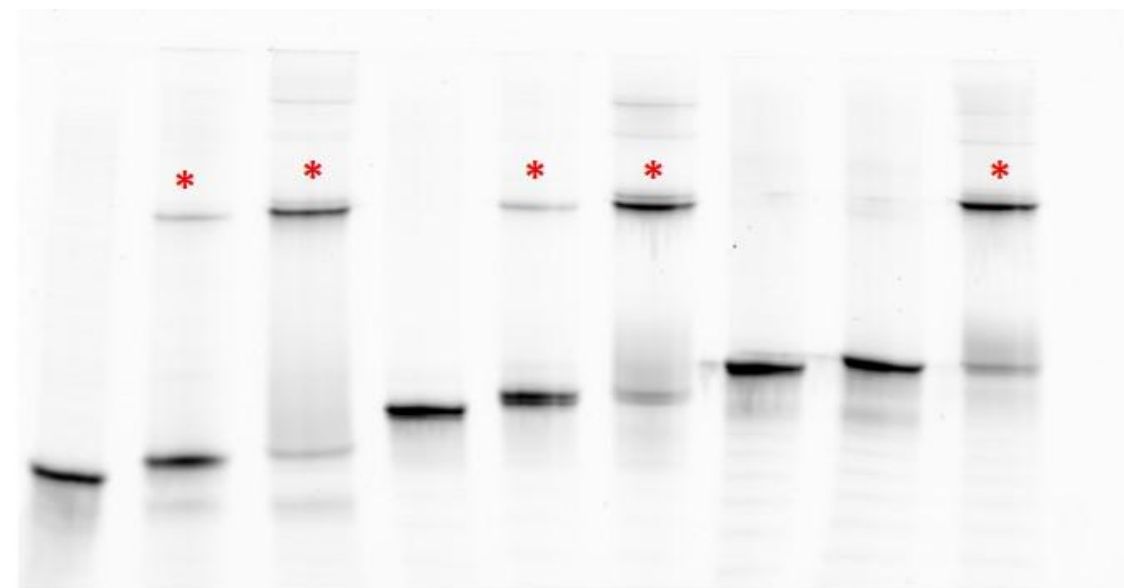

	(1)	(2)	(3)	(4)	(5)	(6)	(7)	(8)	(9)	(10)
30DNA_A^{Natural}C^{Natural}	+	-	-	-	-	-	-	-	-	-
30DNA_A^{pegHalo}C^{Bio}	-	+	-	-	-	-	-	-	-	-
30DNA_A^{pegHalo}C^{pegBio}	-	-	+	-	-	-	-	-	-	-
40DNA_A^{Natural}C^{Natural}	-	-	-	+	-	-	-	-	-	-
40DNA_A^{pegHalo}C^{Bio}	-	-	-	-	+	-	-	-	-	-
40DNA_A^{pegHalo}C^{pegBio}	-	-	-	-	-	+	-	-	-	-
50DNA_A^{Natural}C^{Natural}	-	-	-	-	-	-	+	-	-	-
50DNA_A^{pegHalo}C^{Bio}	-	-	-	-	-	-	-	+	-	-
50DNA_A^{pegHalo}C^{pegBio}	-	-	-	-	-	-	-	-	+	-
mSA	+	+	+	+	+	+	+	+	+	+

Figure S19A. EMSA analysis of 5'-(6-FAM)-labelled **30DNA_A^{X}C^{Y}**, 5'-(6-FAM)-labelled **40DNA_A^{X}C^{Y}** and 5'-(6-FAM)-labelled **50DNA_A^{X}C^{Y}** (X = Natural, pegHalo; Y = Natural, Bio, pegBio) with mSA protein. FAM channel scan of 15% protein native PAGE. (*) DNA-protein complex.

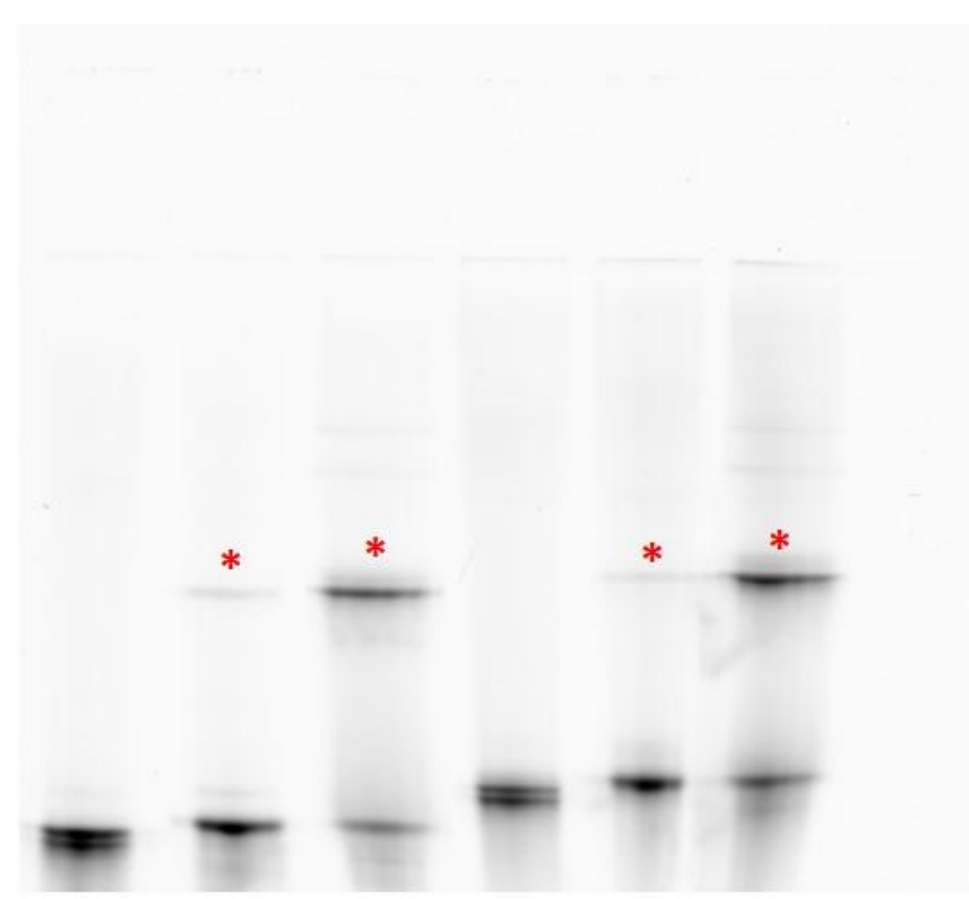

	(1)	(2)	(3)	(4)	(5)	(6)	(7)
60DNA_A^{Natural}C^{Natural}	+	-	-	-	-	-	-
60DNA_A^{pegHalo}C^{Bio}	-	+	-	-	-	-	-
60DNA_A^{pegHalo}C^{pegBio}	-	-	+	-	-	-	-
70DNA_A^{Natural}C^{Natural}	-	-	-	+	-	-	-
70DNA_A^{pegHalo}C^{Bio}	-	-	-	-	+	-	-
70DNA_A^{pegHalo}C^{pegBio}	-	-	-	-	-	+	-
mSA	+	+	+	+	+	+	+

Figure S19B. EMSA analysis of 5'-(6-FAM)-labelled **60DNA_A^{X}C^{Y}**, 5'-(6-FAM)-labelled **70DNA_A^{X}C^{Y}** (X = Natural, pegHalo; Y = Natural, Bio, pegBio) with mSA protein. FAM channel scan of 15% protein native PAGE. (*) DNA-protein complex.

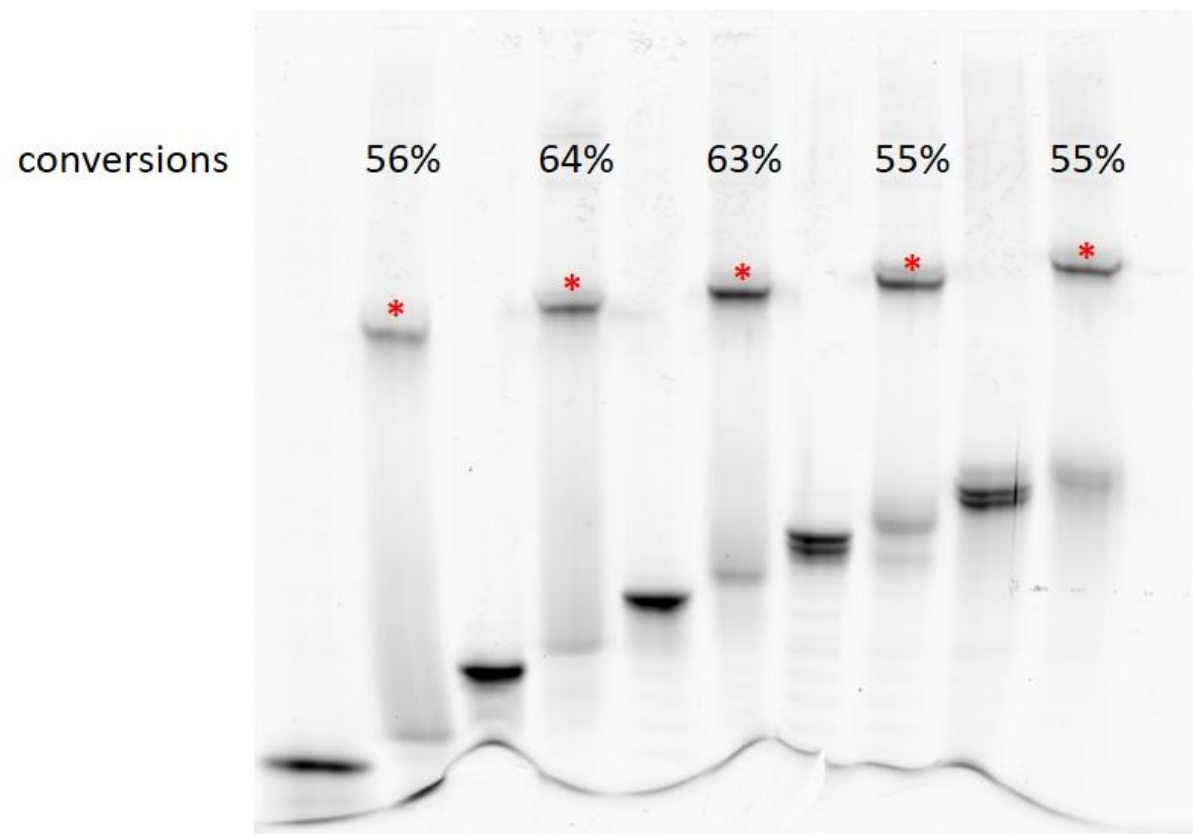

	(1)	(2)	(3)	(4)	(5)	(6)	(7)	(8)	(9)	(10)	(11)
30DNA_ A^{Natural}C^{Natural}	+	-	-	-	-	-	-	-	-	-	-
30DNA_ A^{pegHalo}C^{pegBio}	-	+	-	-	-	-	-	-	-	-	-
40DNA_ A^{Natural}C^{Natural}	-	-	+	-	-	-	-	-	-	-	-
40DNA_ A^{pegHalo}C^{pegBio}	-	-	-	+	-	-	-	-	-	-	-
50DNA_ A^{Natural}C^{Natural}	-	-	-	-	+	-	-	-	-	-	-
50DNA_ A^{pegHalo}C^{pegBio}	-	-	-	-	-	+	-	-	-	-	-
60DNA_ A^{Natural}C^{Natural}	-	-	-	-	-	-	+	-	-	-	-
60DNA_ A^{pegHalo}C^{pegBio}	-	-	-	-	-	-	-	+	-	-	-
70DNA_ A^{Natural}C^{Natural}	-	-	-	-	-	-	-	-	+	-	-
70DNA_ A^{pegHalo}C^{pegBio}	-	-	-	-	-	-	-	-	-	+	-
mSA	+	+	+	+	+	+	+	+	+	+	+

Figure S20. EMSA analysis of selected samples 5'-(6-FAM)-labelled **30DNA_A^{X}C^{Y}**, 5'-(6-FAM)-labelled **40DNA**_A^{X}C^{Y}, 5'-(6-FAM)-labelled **50DNA_A^{X}C^{Y}**, 5'-(6-FAM)-labelled **60DNA_A^{X}C^{Y}** and 5'-(6-FAM)-labelled **70DNA_A^{X}C^{Y}** (X = Natural, pegHalo; Y = Natural, pegBio) with mSA protein. FAM channel scan of 12% protein native PAGE. (*) DNA-protein complex. Calculated conversions of complexes formation.

2.9.10. EMSA of double modified dsDNA (30-, 40-, 50-, 60- and 70-mer) with combination of recombinant mSA protein and recombinant GST-Halotag protein

The reaction mixture (10 µL) containing one of 5'-(6-FAM)-labelled **30DNA_A^{pegHalo}C^{X}**, 5'-(6-FAM)-labelled **40DNA_A^{pegHalo}C^{X}**, 5'-(6-FAM)-labelled **50DNA_A^{pegHalo}C^{X}**, 5'-(6-FAM)-labelled **60DNA_A^{pegHalo}C^{X}** or 5'-(6-FAM)-labelled **70DNA_A^{pegHalo}C^{X}** (X = Bio, pegBio; 0.5 µM, 1 equiv.; prepared as described above in Section 2.5.) in PBS2 buffer was incubated with mSA protein (50 µM, 100 equiv.), or with GST-Halotag protein (25 µM, 50 equiv.), or with combination of mSA protein (50 µM, 100 equiv.) and GST-Halotag protein (25 µM, 50 equiv.) at 37 °C for 17 h in a thermal cycler (with heated lid at 65 °C). After this time, the samples were combined with 2 µL of sterile glycerol, separated by 7% protein native PAGE and visualized using fluorescence imaging (Figure S21A,B).

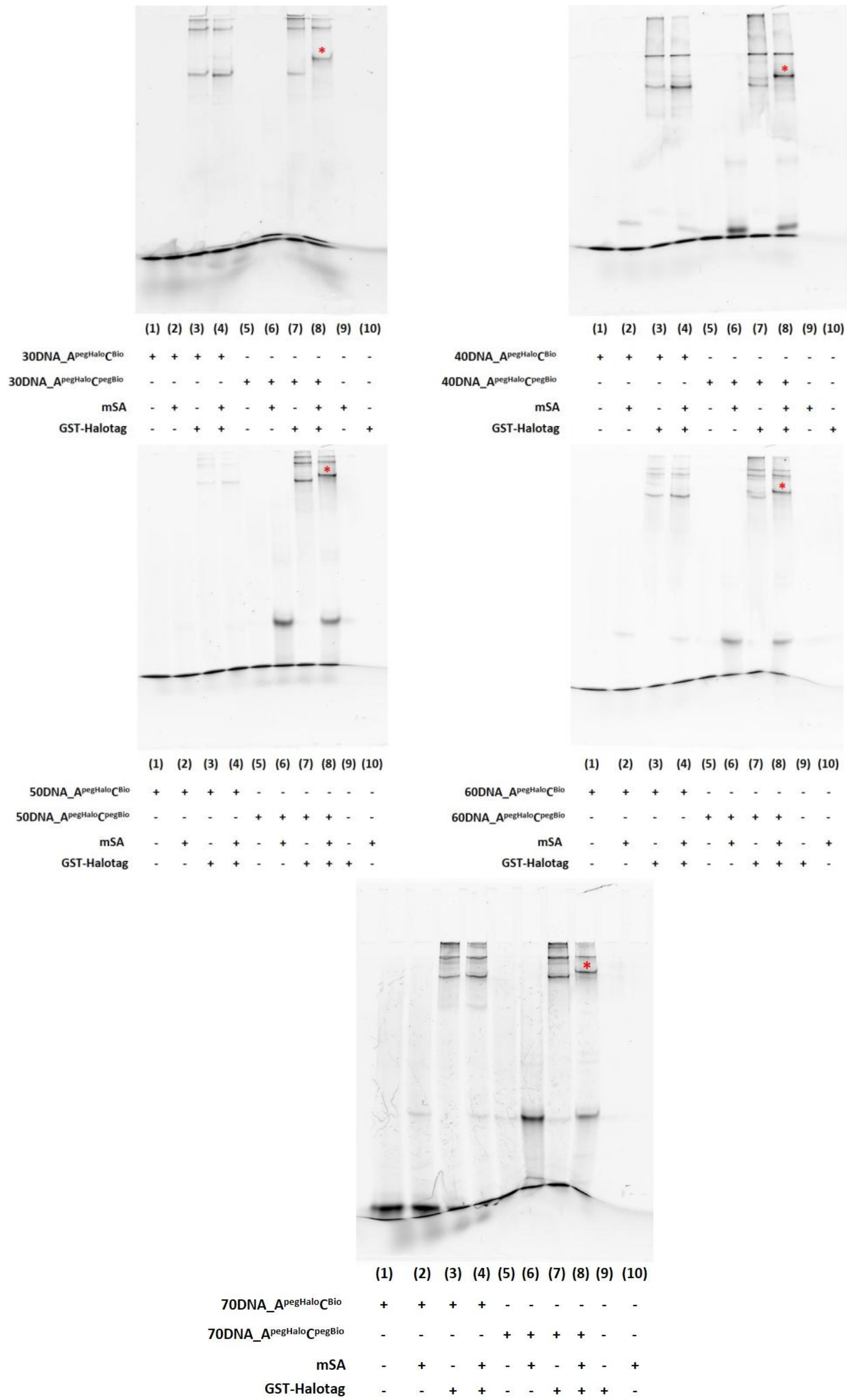

Figure S21A. EMSA analysis of 5'-(6-FAM)-labelled **30DNA_A^{pegHalo}C^{X}**, 5'-(6-FAM)-labelled **40DNA_A^{pegHalo}C^{X}**, 5'-(6-FAM)-labelled **50DNA_A^{pegHalo}C^{X}**, 5'-(6-FAM)-labelled **60DNA_A^{pegHalo}C^{X}** and 5'-(6-FAM)-labelled **70DNA_A^{pegHalo}C^{X}** (X = Bio, pegBio) with mSA protein, with GST-Halotag protein, or with combination of mSA and GST-Halotag proteins. FAM channel scan of 7% protein native PAGE. (*) Ternary DNA-Halotag-mSA complex.

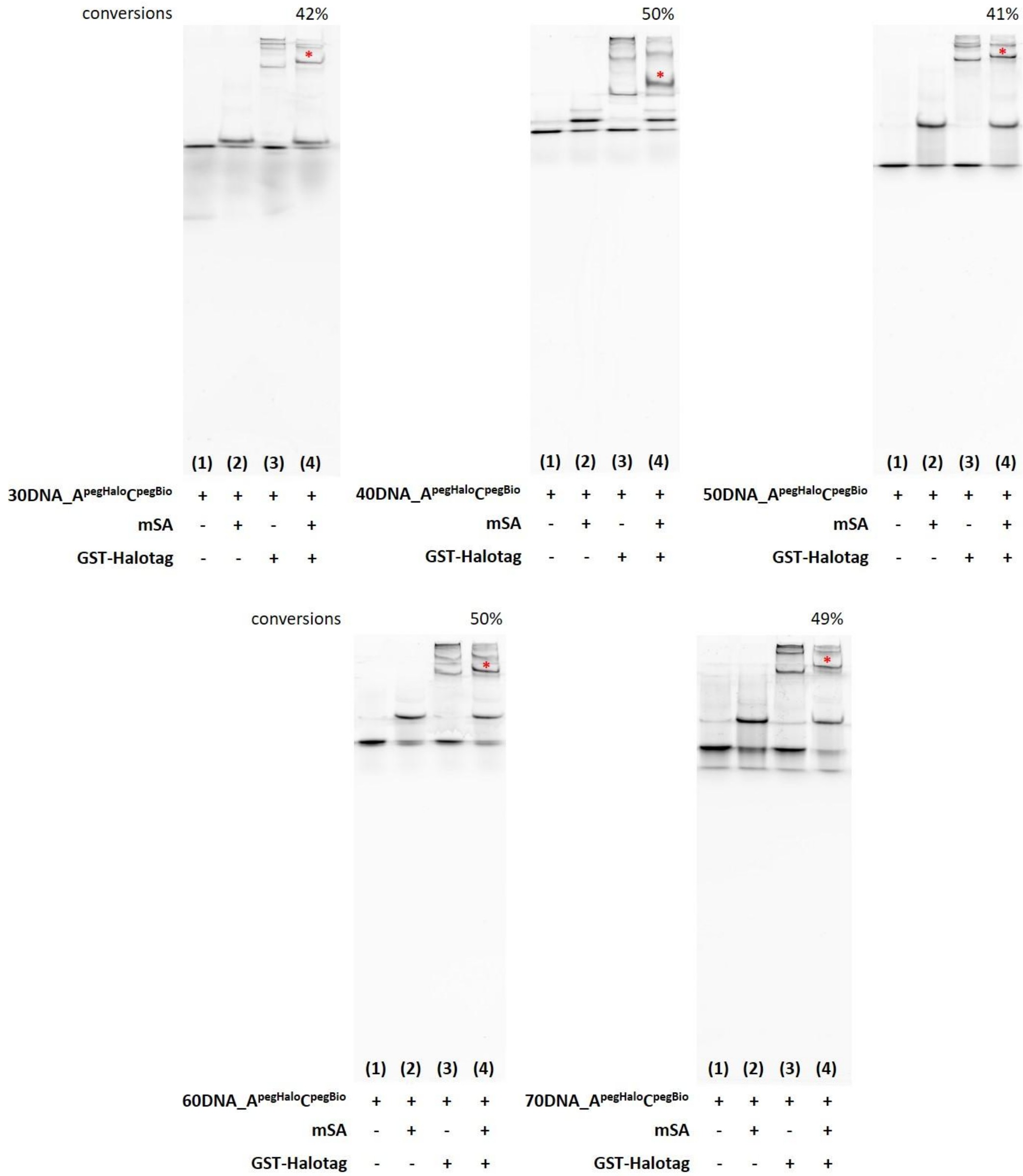

Figure S21B. EMSA analysis of selected samples 5'-(6-FAM)-labelled **30DNA_A^{pegHalo}C^{pegBio}**, 5'-(6-FAM)-labelled **40DNA_A^{pegHalo}C^{pegBio}**, 5'-(6-FAM)-labelled **50DNA_A^{pegHalo}C^{pegBio}**, 5'-(6-FAM)-labelled **60DNA_A^{pegHalo}C^{pegBio}** and 5'-(6-FAM)-labelled **70DNA_A^{pegHalo}C^{pegBio}** with mSA protein, with GST-Halotag protein, or with combination of mSA and GST-Halotag proteins. FAM channel scan of 7% protein native PAGE after 1 h of electrophoretic separation. (*) Ternary DNA-Halotag-mSA complex. Calculated conversions of ternary complexes formation.

2.9.11. EMSA of double modified ssDNA (32-mer) with combination of recombinant mSA iFluor 555 protein and recombinant GST-Halotag protein

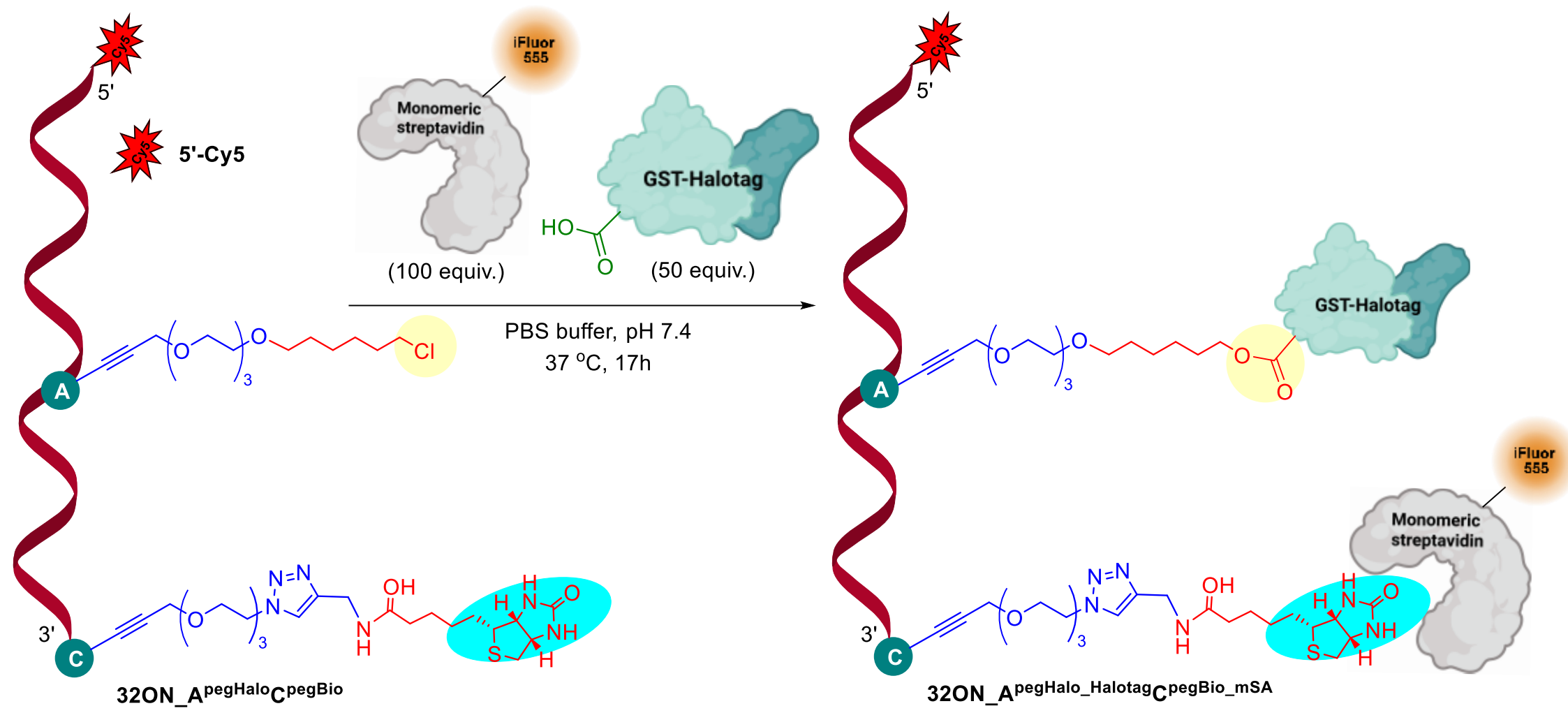

Scheme S6. Reaction of **32ON_A^{pegHalo}C^{pegBio}** with both GST-Halotag and monomeric streptavidin iFluor 555 proteins at the same time.

The reaction mixture (10 µL) containing 5'-(Cy5)-labelled **32ON_A^{pegHalo}C^{pegBio}** (0.5 µM, 1 equiv.; prepared as described above in Section 2.4.) in PBS2 buffer was incubated with mSA iFluor 555 protein (50 µM, 100 equiv.), or with GST-Halotag protein (25 µM, 50 equiv.), or with combination of mSA iFluor 555 protein (50 µM, 100 equiv.) and GST-Halotag protein (25 µM, 50 equiv.) at 37 °C for 17 h in a thermal cycler (with heated lid at 65 °C). After this time, the samples were combined with 2 µL of sterile glycerol, separated by 7% protein native PAGE and visualized using fluorescence imaging (Figure S22).

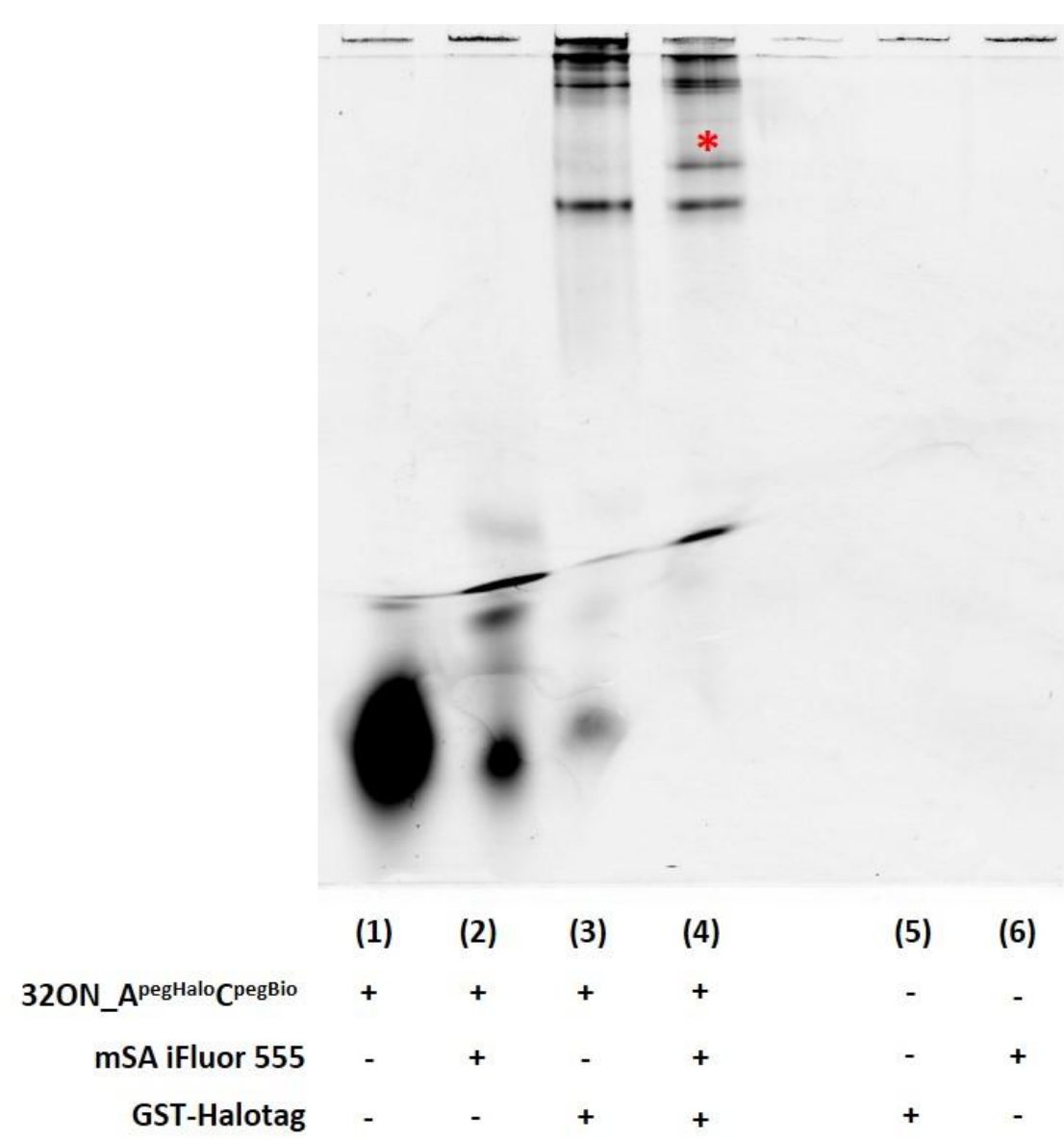

Figure S22. EMSA analysis of 5'-(Cy5)-labelled **32ON_A^{pegHalo}C^{pegBio}** with mSA iFluor 555 protein, with GST-Halotag protein, or with combination of mSA iFluor 555 and GST-Halotag proteins. Cy5 channel scan of 7% protein native PAGE. (*) Ternary DNA-Halotag-mSA iFluor 555 complex.

2.10. Representative examples of DNA-Halotag-mSA assemblies

Visualization of DNA-Halotag-mSA assemblies based on AlphaFold 3 and molecular modeling. The inputs were: unmodified sequences of 30-mer and 70-mer dsDNA; protein sequences from commercial supplier EastPort LifeScience for GST-Halotag protein, and Protein Data Bank (PDB ID: 4JNJ) for mSA protein; SMILES for the ligands. Non-covalent complexes were modified in open-source PyMol to create the covalently-bonded ones. Note that only the relative sizes of molecules but not their conformations are realistic. Color coding: DNA forward strand: red; DNA reverse strand: blue; mSA protein: grey; Halotag protein: green; GST tag: dark green; biotin tag carbons: cyan; Halotag ligand: yellow (Figure S23).

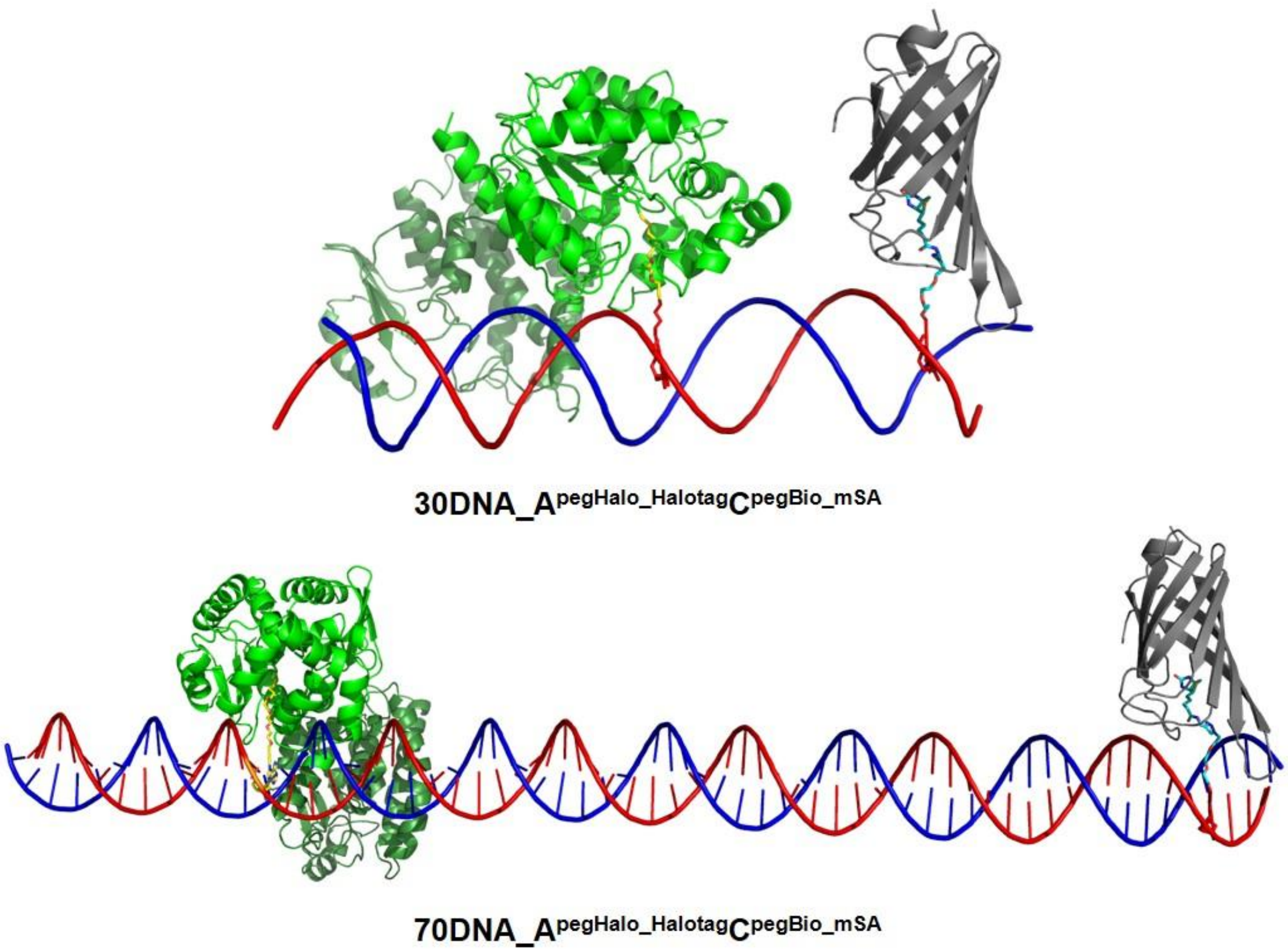

Figure S23. Representative examples of DNA-Halotag-mSA assemblies for **30DNA_A**$^{pegHalo\_Halotag}$**C**$^{pegBio\_mSA}$ and **70DNA_A**$^{pegHalo\_Halotag}$**C**$^{pegBio\_mSA}$.

2.11. Fluorescent Nickel-plate binding affinity assay (FNBA)

His-tagged mSA protein (20 pmol, 250 nM in 80 µL of PBS2 buffer) was immobilized on Nickel-coated Plate by incubation for 1 hour at 25 °C with shaking at 500 rpm. Wells were washed twice with 200 µL of PBS2 buffer containing 0.05% [v/v] Tween-20, followed by one wash with 200 µL of PBS2 buffer. Then, 80 µL of 5'-(Cy5)-labelled **19DNA_C**^{pegBio} (prepared as described above in Section 2.5.) at different concentrations (ranging from 150 nM to 0.31 nM) in PBS2 buffer were

added to the wells and incubated for 1 hour at 25 °C with shaking at 500 rpm. After incubation, wells were washed three times with 200 µL of PBS2 buffer. PBS2 buffer (100 µL) was added to each well, and fluorescence intensity was measured. Binding curve and dissociation constant (K_D) were shown in Figure S24.

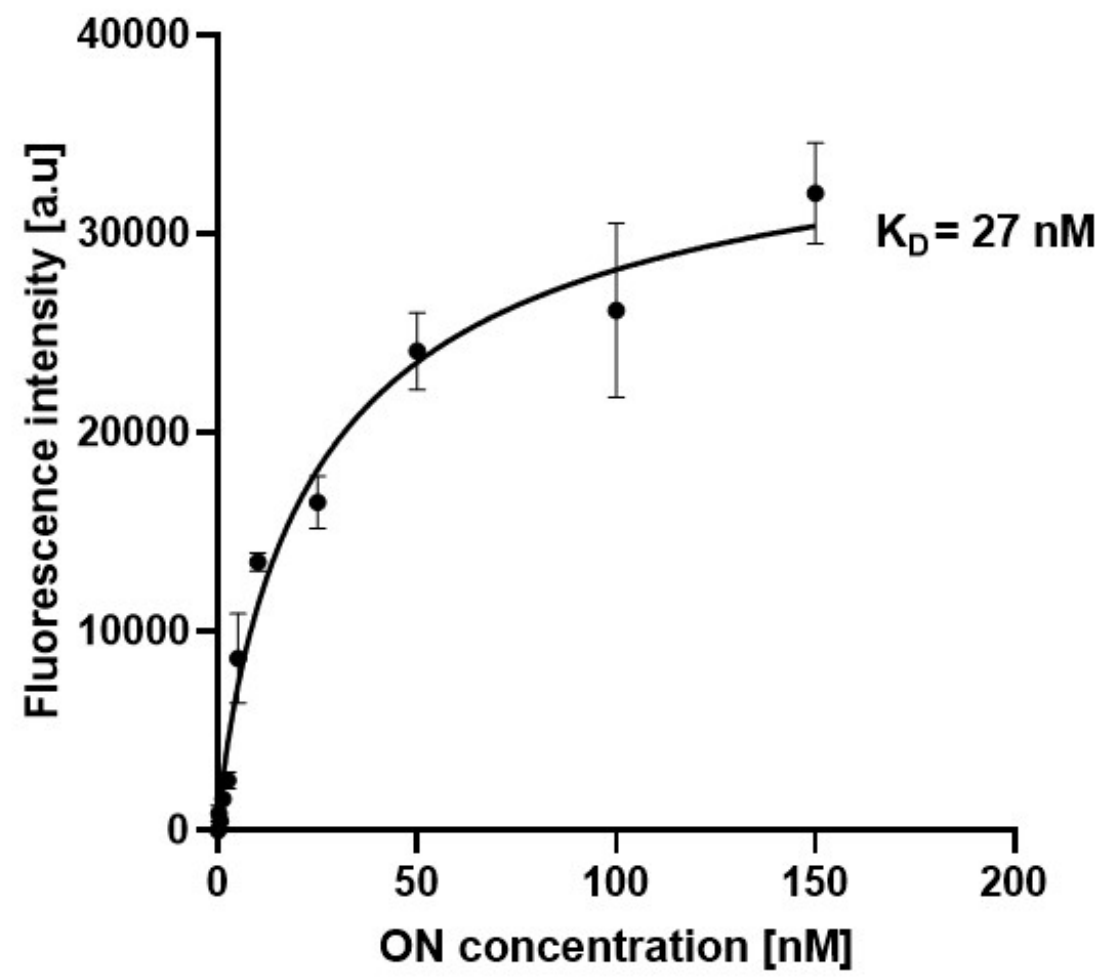

Figure S24. Fluorescent Nickel-plate binding affinity assay of 5'-(Cy5)-labelled **19DNA_C^{pegBio}** toward His-tagged mSA protein. Error bars represent the mean ± s.d. from three technical replicates.

2.12. Modified TUNEL assay using Terminal Deoxynucleotidyl Transferase (TdT) mediated incorporation of dC^{pegBio}TP and flow cytometric detection with recombinant mSA iFluor 647 protein

Cell culture

Human adenocarcinoma HeLa cells were obtained from the America Type Culture Collection (ATCC, CCL-2) and cultured in RPMI-1640 medium (Dutch modification, Sigma-Aldrich, Cat. No. R7638) supplemented with 10% heat inactivated fetal bovine serum (Sigma-Aldrich, Cat. No. F7524) at 37 °C in a humidified incubator containing 5% CO_2. Cells were passaged using 0.25% trypsin–EDTA (Sigma-Aldrich, Cat. No. T4049) and were used for the assay at approximately 80% confluence.

Cell harvesting, fixation, and permeabilization

HeLa cells were trypsinized, neutralized with complete culture medium, collected, and counted. For four flow-cytometry samples, a total of 2.0×10^6 cells was used, corresponding to 5.0×10^5 cells per sample. Cells were washed twice with PBS by centrifugation at 300 × g for 5 min at room temperature.

The cell pellet was resuspended in 100 µL of PBS to obtain a homogeneous single-cell suspension before fixation. An equal volume of 4% formaldehyde (BD Cytofix, BD Biosciences, Cat. No. 554655) was added to the cell suspension while mixing, giving a final formaldehyde concentration of 2%. Cells were incubated for 10 min at room temperature. The fixation reaction was diluted by

addition of 700 µL of PBS containing 2% bovine serum albumin (BSA), and cells were collected by centrifugation at 500 × g for 5 min. Cells were then washed once with 800 µL of PBS.
For permeabilization, the fixed cell pellet was resuspended in 400 µL of PBS containing 0.25% Triton X-100 and incubated for 15 min at room temperature. Cells were then collected by centrifugation at 500 × g for 5 min and washed once with PBS.
After permeabilization, the cells were divided into four samples containing approximately 5.0×10^5 cells each:

1. Fixed/permeabilized unstained control, (**"Untreated cells"**, Figure S25A);
2. TdT negative control: cells treated with DNase I, **dC^{pegBio}TP**, mSA iFluor 647 protein (**"Without TdT"**, Figure S25B);
3. DNase I negative control: cells treated with TdT, **dC^{pegBio}TP**, mSA iFluor 647 protein (**"Without DNase I"**, Figure S25C);
4. Complete-reaction sample: cells treated with DNase I, TdT, **dC^{pegBio}TP**, mSA iFluor 647 protein (**"With TdT and DNase I"**, Figure S25D).

DNase I treatment

DNase I treatment was used to generate DNA strand breaks, thereby providing a positive control with accessible 3′-OH DNA ends for TdT mediated incorporation of **dC^{pegBio}TP**.
The TdT negative control, DNase I negative control, and complete-reaction sample were washed once with DNase I reaction buffer consisting of 50 mM Tris-HCl, pH 7.5, 10 mM $MgCl_2$, and 1 mg/mL BSA. Cells were collected by centrifugation at 500 × g for 5 min.
For DNase I treatment, the TdT negative control and complete-reaction sample were resuspended in 200 µL of DNase I reaction buffer containing 10 U DNase I (Roche, Cat. No. 04536282001) per sample and incubated for 20 min at room temperature. The DNase I negative control was incubated in 200 µL of DNase I reaction buffer without DNase I under otherwise identical conditions.
After incubation, 700 µL of PBS were added to each sample. Cells were collected by centrifugation at 500 × g for 5 min, washed once with PBS, washed once with Milli-Q water, and then washed once with 1× TdT reaction buffer.

TdT mediated incorporation of dC^{pegBio}TP

After centrifugation at 500 × g for 5 min, the DNase I negative control and complete-reaction sample were resuspended in 50 µL of TdT reaction mixture containing 1× TdT reaction buffer, 20 U of Terminal Deoxynucleotidyl Transferase (TdT), and 10 µM of **dC^{pegBio}TP**. The TdT negative control was resuspended in the same reaction mixture, except that TdT was omitted. The fixed/permeabilized unstained control was not incubated with TdT, **dC^{pegBio}TP**, or mSA iFluor 647 protein.
The samples were incubated for 60 min at 37 °C. After incubation, 500 µL of PBS containing 1% BSA was added to each sample. Cells were collected by centrifugation at 500 × g for 5 min, and

the supernatant was removed. This washing step was repeated once more with PBS containing 1% BSA.

mSA iFluor 647 protein staining

mSA iFluor 647 protein was diluted from a 1 mg/mL stock solution to a 1 µg/mL solution in PBS containing 1% BSA. Each sample was resuspended in 100 µL of the diluted mSA iFluor 647 protein solution and incubated for 30 min at room temperature protected from light.
After staining, cells were washed once with 500 µL of PBS containing 1% BSA and once with PBS containing 1% BSA and 0.05% Triton X-100. Finally, cells were resuspended in 100 µL of PBS and analyzed by flow cytometry

Flow cytometry acquisition and analysis

Flow cytometry analysis was performed on a CytoFLEX LX flow cytometer (Beckman Coulter) using CytExpert software version 2.3.1.22. iFluor 647 fluorescence was excited with the red laser (638 nm) and detected in the APC channel using a 660/10 nm band pass filter. Detector gains were set using the unstained fixed/permeabilized control and the complete-reaction samples, and identical acquisition settings were used for all samples within the experiment. Samples were acquired at medium flow rate (30 µL/min). At least 15,000 total events were recorded per sample. Since only iFluor 647 fluorescence was measured, fluorescence compensation was not required.
Data were analyzed using CytExpert version 2.6.0.105. Debris was excluded using a forward-scatter area (FSC-A) versus side-scatter area (SSC-A) gate. Single cells were selected using FSC-A versus FSC-H. iFluor 647 positive cells were quantified as the percentage of singlet gated events exceeding a threshold defined from the TdT negative control sample. The same gate was then applied to all samples. Results were reported as the percentage of iFluor 647 positive cells (Figure S25A,B,C,D).

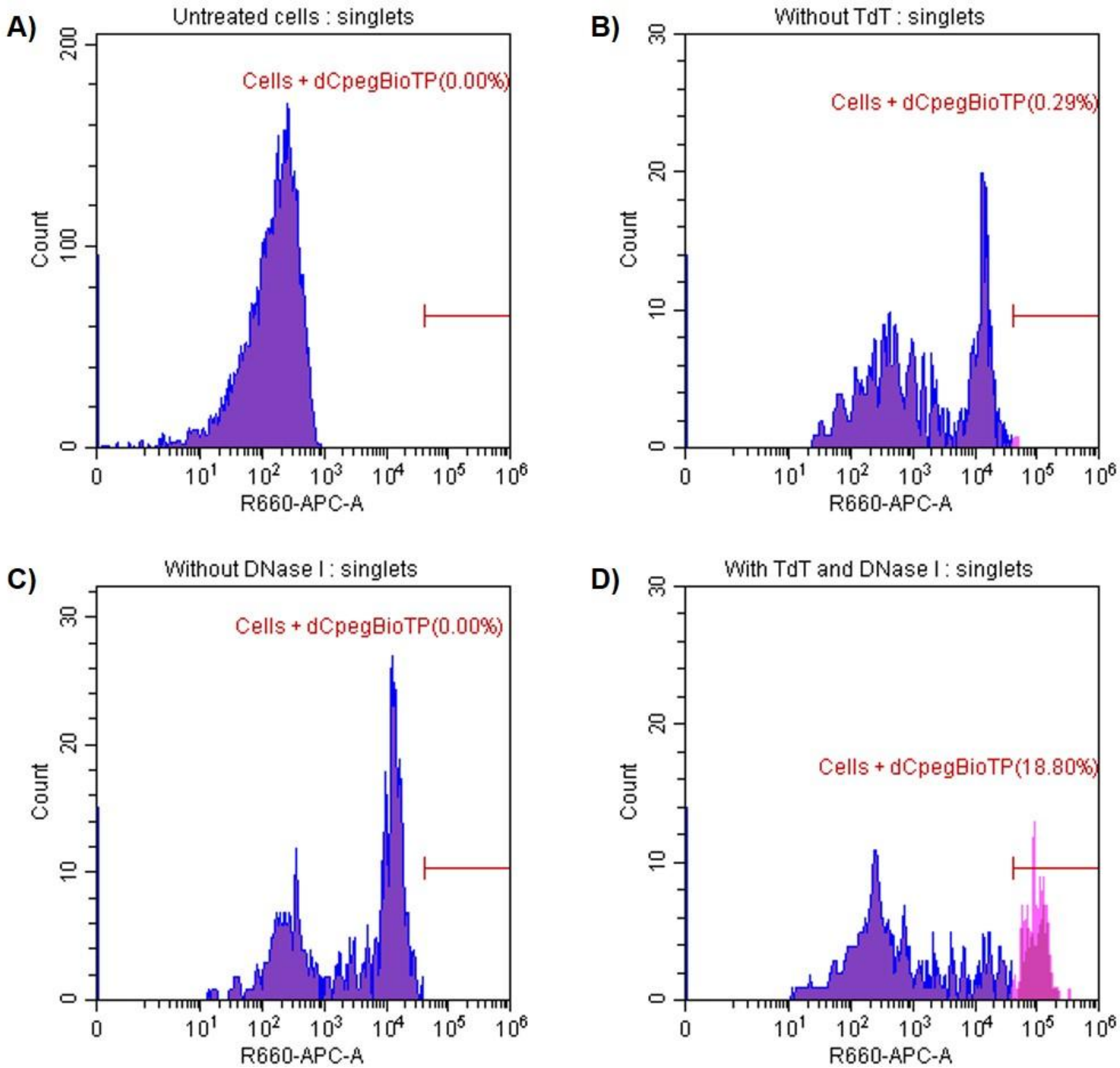

Figure S25. Flow cytometry analysis. Results reported as the percentage of iFluor 647 positive cells. (A) **Untreated cells**: fixed/permeabilized unstained control. (B) **Without TdT** (TdT negative control): cells treated with DNase I, **dC^{pegBio}TP**, mSA iFluor 647 protein. (C) **Without DNase I** (DNase I negative control): cells treated with TdT, **dC^{pegBio}TP**, mSA iFluor 647 protein. (D) **With TdT and DNase I** (complete-reaction sample): cells treated with DNase I, TdT, **dC^{pegBio}TP**, mSA iFluor 647 protein.

3. Copies of MALDI-TOF and LC-MS mass spectra

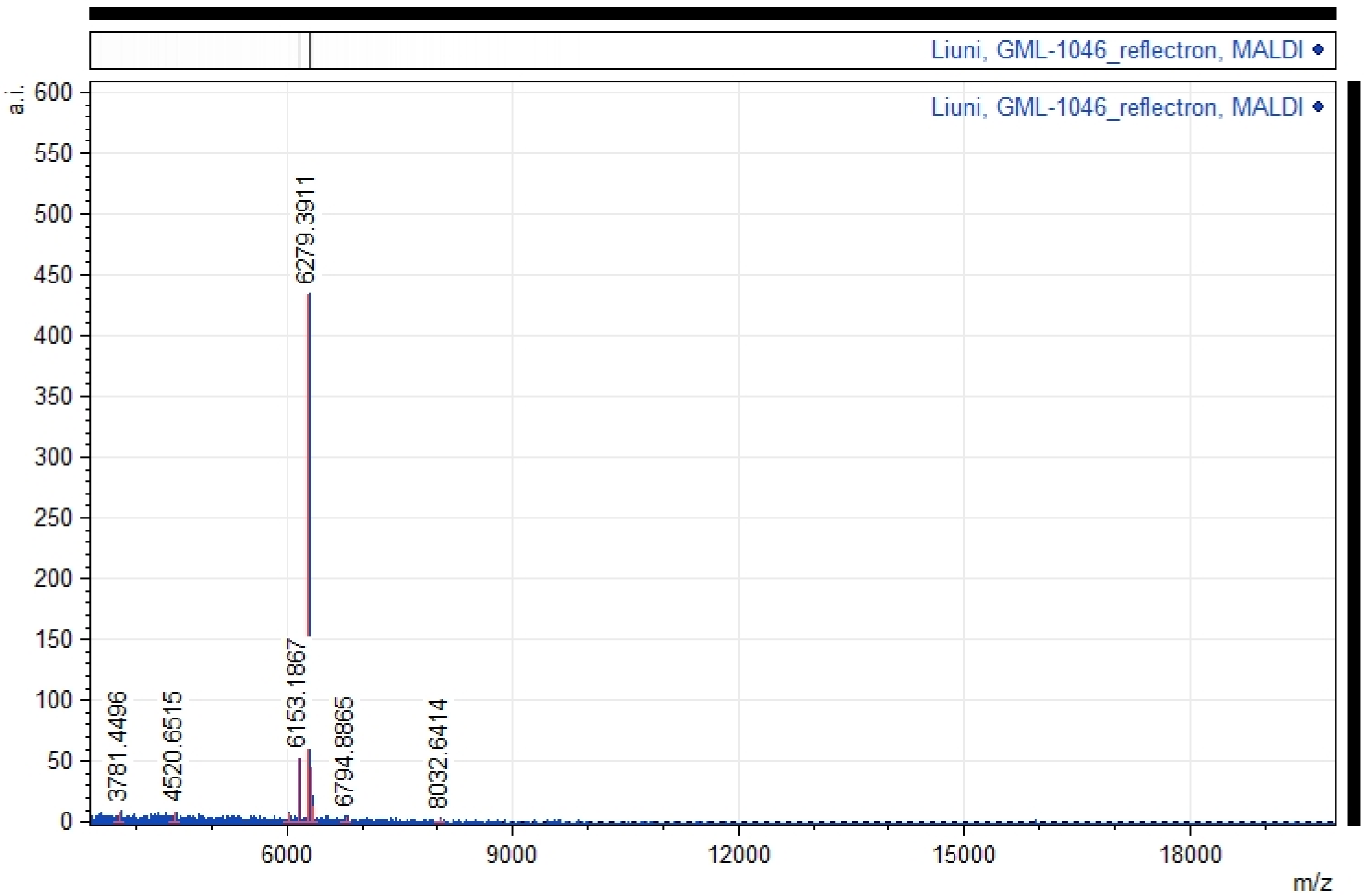

Figure S26. MALDI-TOF spectrum of **19ON_A^{pegHalo}**: calculated: 6279.06 Da; found: 6279.39 Da; Δ = 0.3.

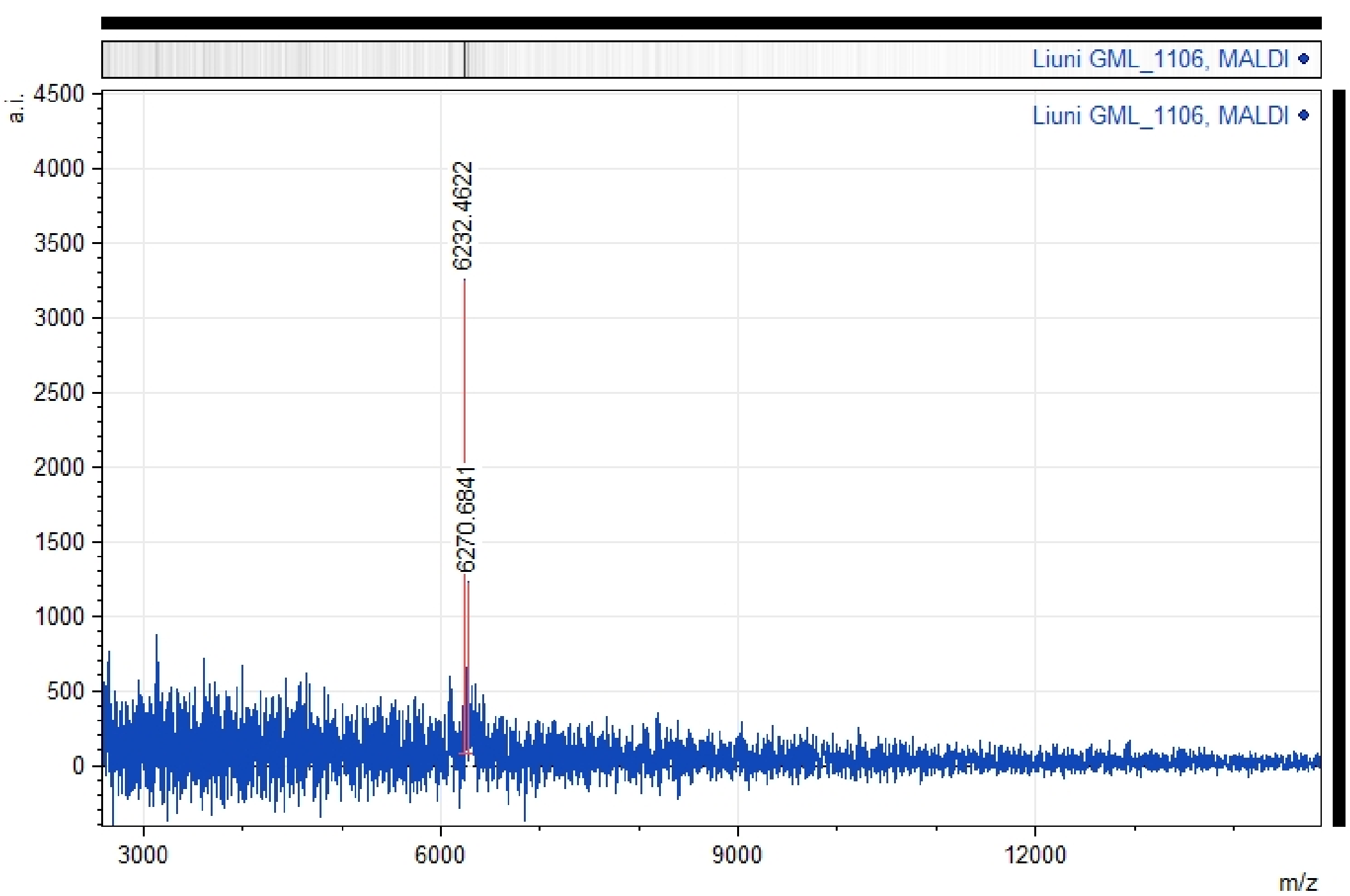

Figure S27. MALDI-TOF spectrum of **19ON_C^{Bio}**: calculated: 6230.02 Da; found: 6232.46 Da; Δ = 2.4. The peak at *m/z* = 6270.68 is assigned to **19ON_C^{Bio}** plus K^+.

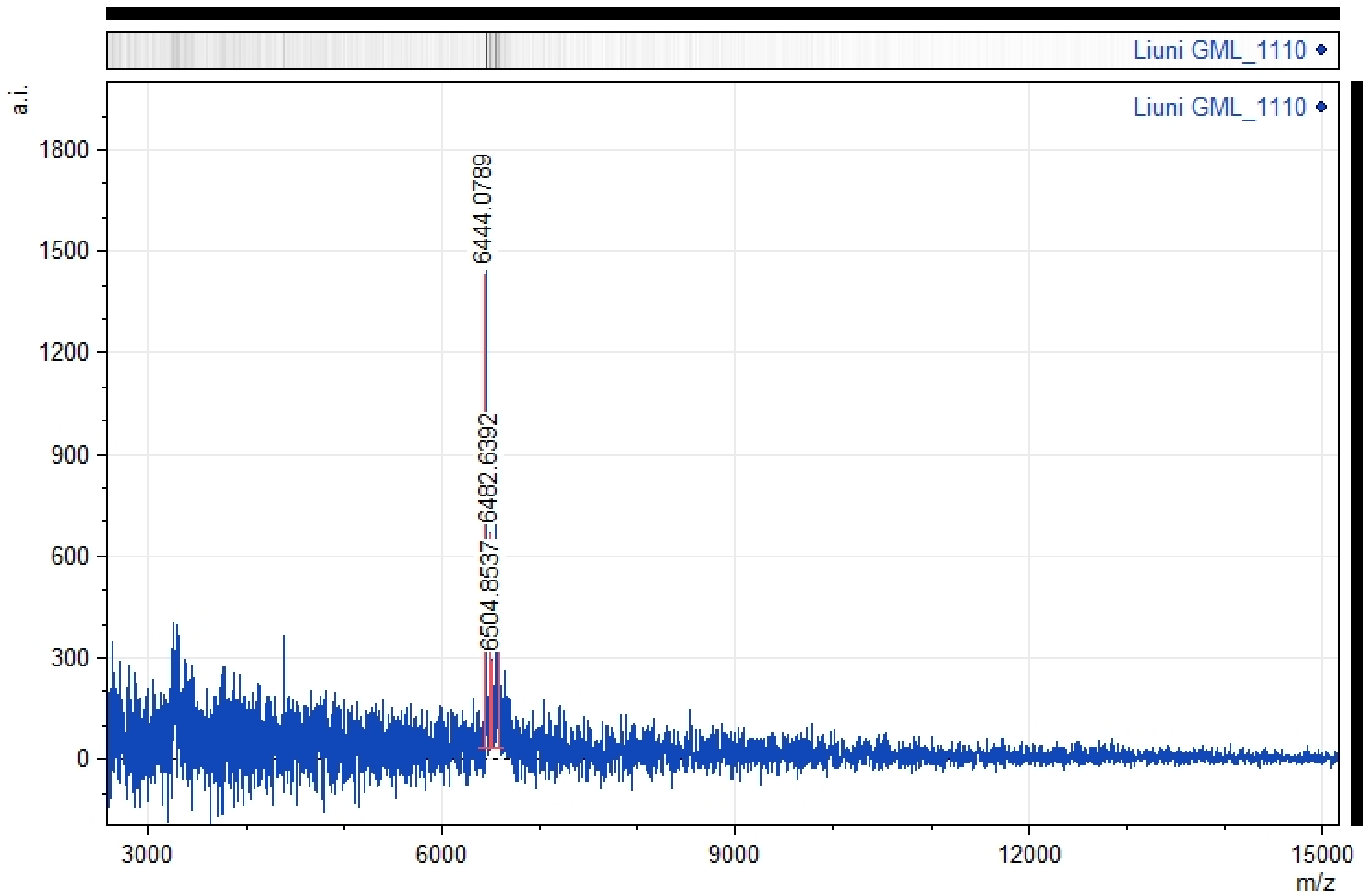

Figure S28. MALDI-TOF spectrum of **19ON_C^{pegBio}**: calculated: 6443.13 Da; found: 6444.07 Da; Δ = 0.9. The peak at *m/z* = 6482.63 is assigned to **19ON_C^{pegBio}** plus K^+.

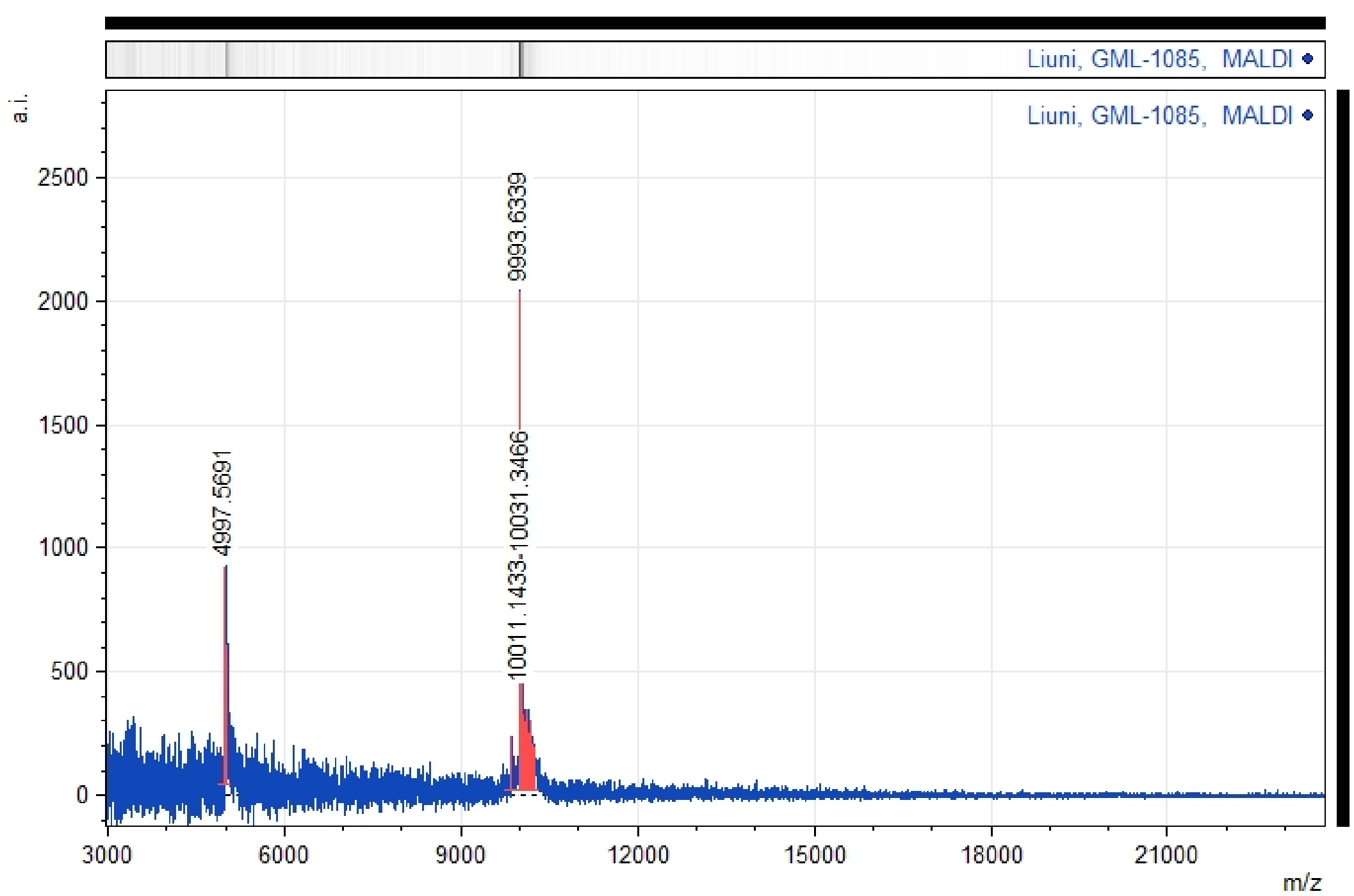

Figure S29. MALDI-TOF spectrum of **30ON_A^{pegHalo}**: calculated: 9993.42 Da; found: 9993.63 Da; Δ = 0.2. The peak at *m/z* = 4997.56 is assigned to **30ON_A^{pegHalo}** with *z*=2; the peak at *m/z* = 10011.14 is assigned to **30ON_A^{pegHalo}** plus H_2O; the peak at *m/z*= 10031.34 is assigned to **30ON_A^{pegHalo}** plus K^+.

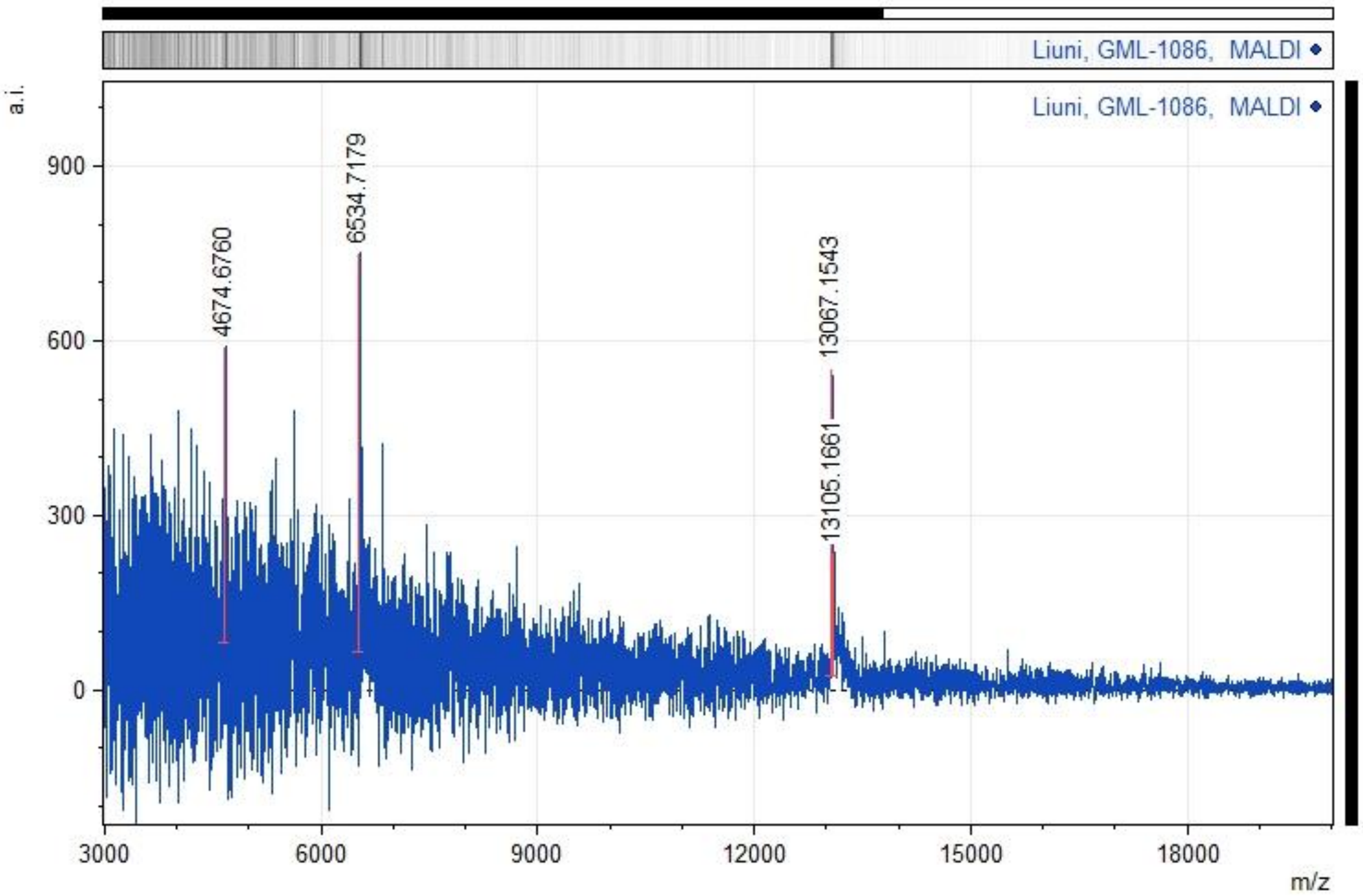

Figure S30. MALDI-TOF spectrum of **40ON_A^{pegHalo}**: calculated: 13065.42 Da; found: 13067.15 Da; Δ = 1.7. The peak at *m/z* = 4674.67 is assigned to primer **Prim248short**; the peak at *m/z* = 6534.71 is assigned to **40ON_A^{pegHalo}** with *z*=2; the peak at *m/z* = 13105.16 is assigned to **40ON_A^{pegHalo}** plus K^+.

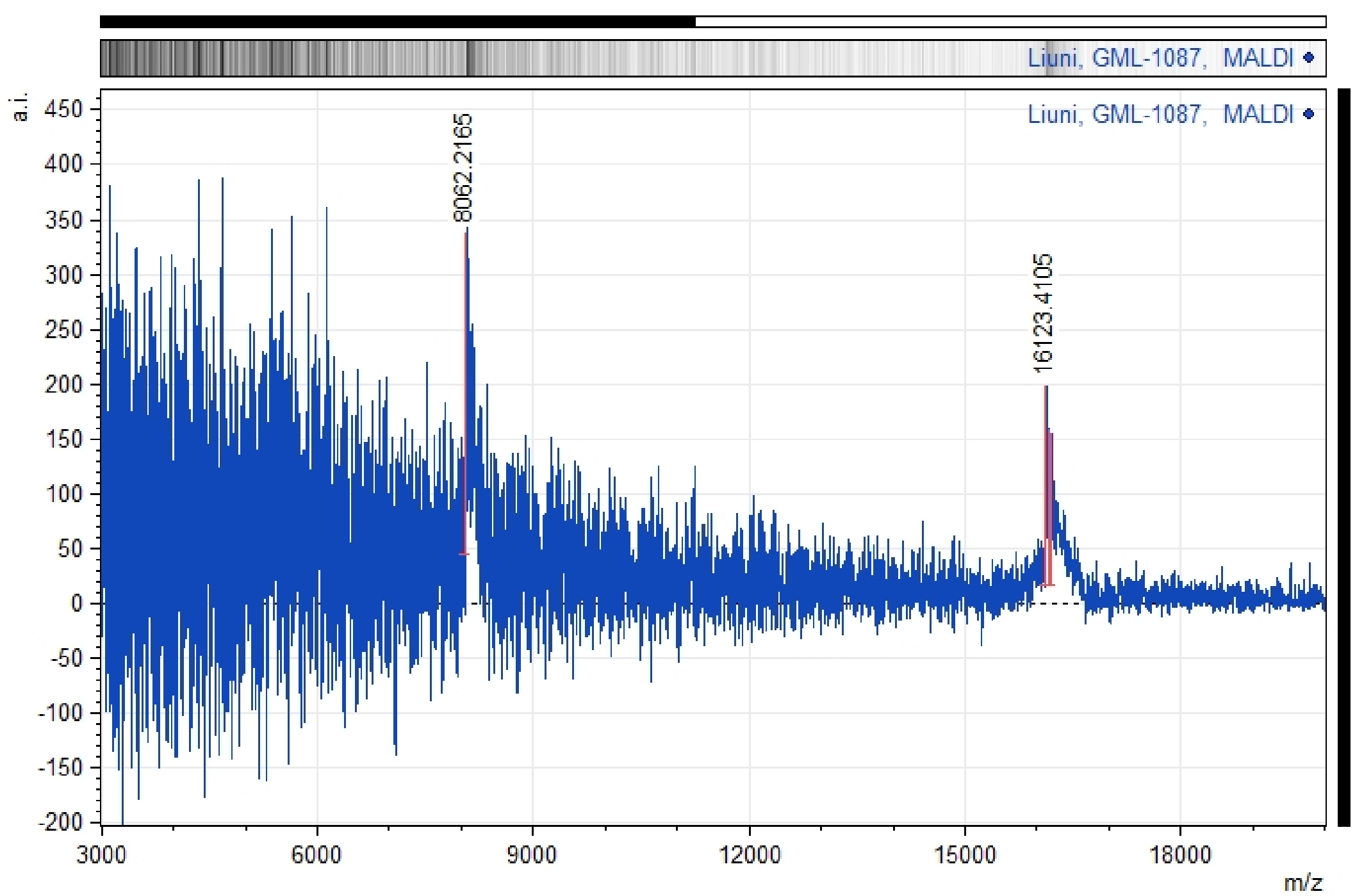

Figure S31. MALDI-TOF spectrum of **50ON_A^{pegHalo}**: calculated: 16122.32 Da; found: 16123.41 Da; Δ = 1.1. The peak at *m/z* = 8062.21 is assigned to **50ON_A^{pegHalo}** with *z*=2.

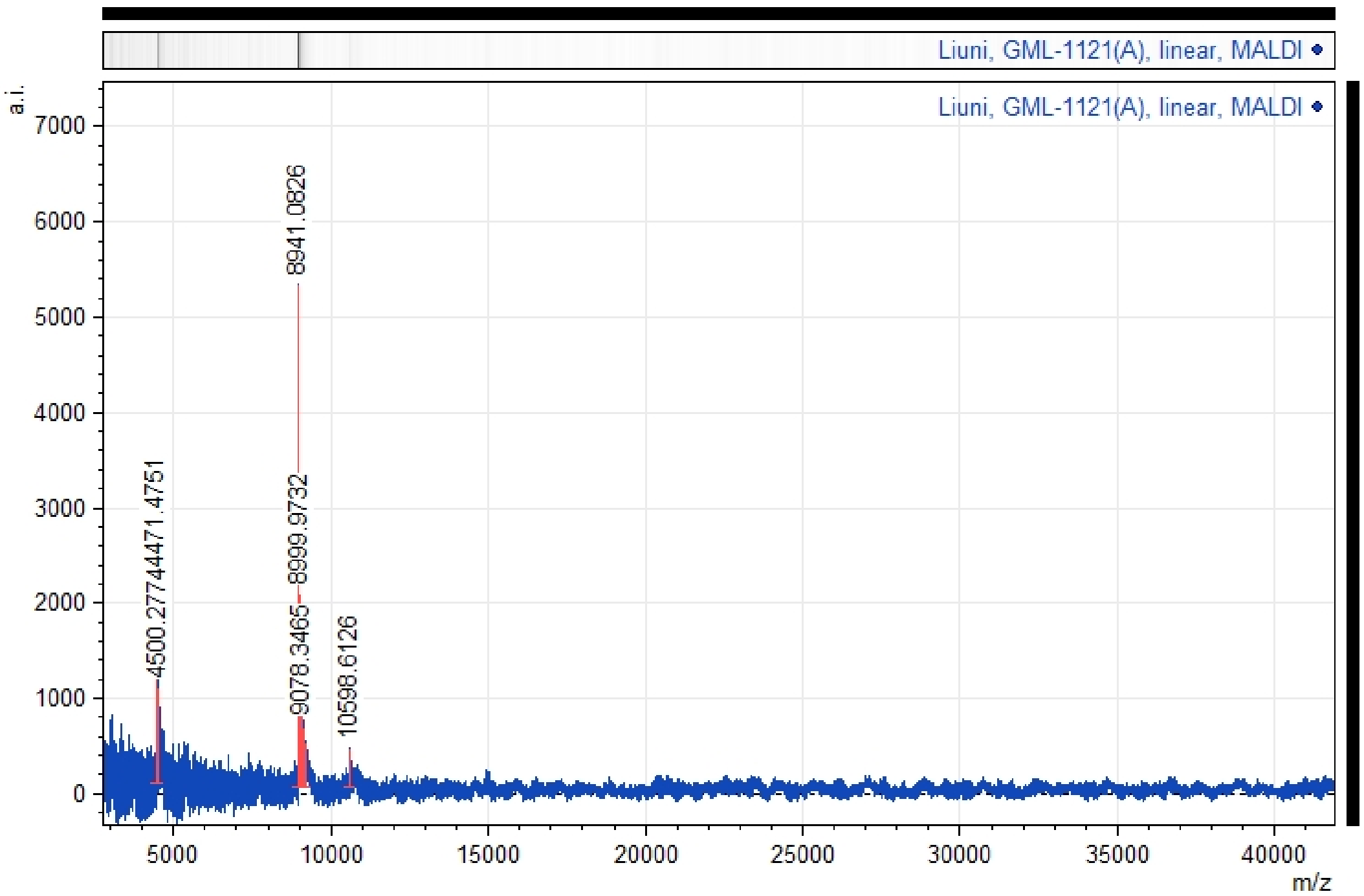

Figure S32. MALDI-TOF spectrum of 5'-(Cy5)-labelled **30DNA_A^pegHalo^C^Bio^**; mass calculated for 5'-(Cy5)-labelled **30ON_A^pegHalo^C^Bio^**: calculated 10598.18 Da; found 10598.61 Da; Δ = 0.4. The peak at *m/z* = 4471.14 is assigned to template **T30-10** with *z*=2; the peak at *m/z* = 8941.08 is assigned to template **T30-10**.

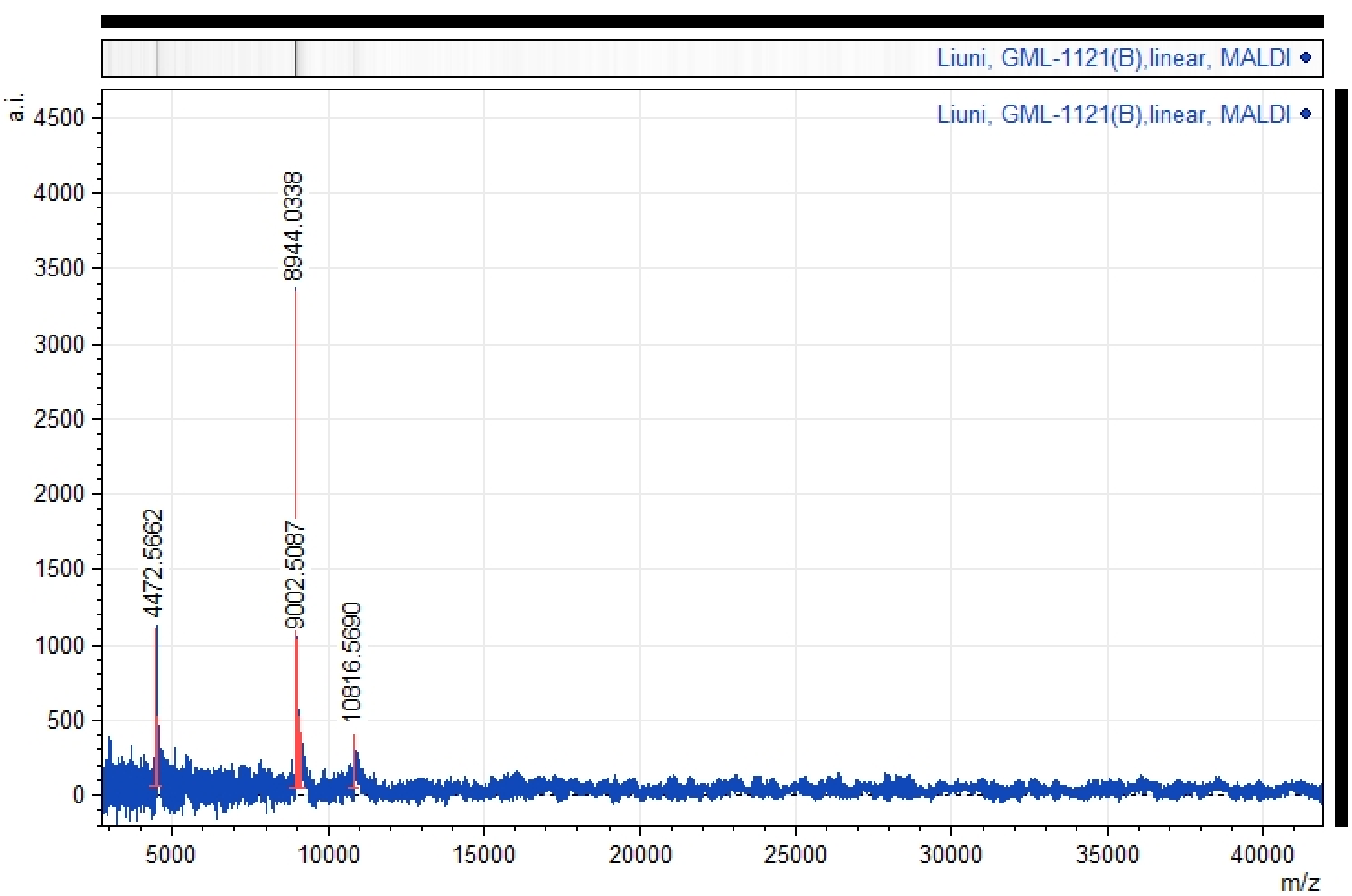

Figure S33. MALDI-TOF spectrum of 5'-(Cy5)-labelled **30DNA_A^pegHalo^C^pegBio^**; mass calculated for 5'-(Cy5)-labelled **30ON_A^pegHalo^C^pegBio^**: calculated 10811.29 Da; found 10816.56 Da; Δ = 5.3. The peak at *m/z* = 4472.56 is assigned to template **T30-10** with *z*=2; the peak at *m/z* = 8944.03 is assigned to template **T30-10**.

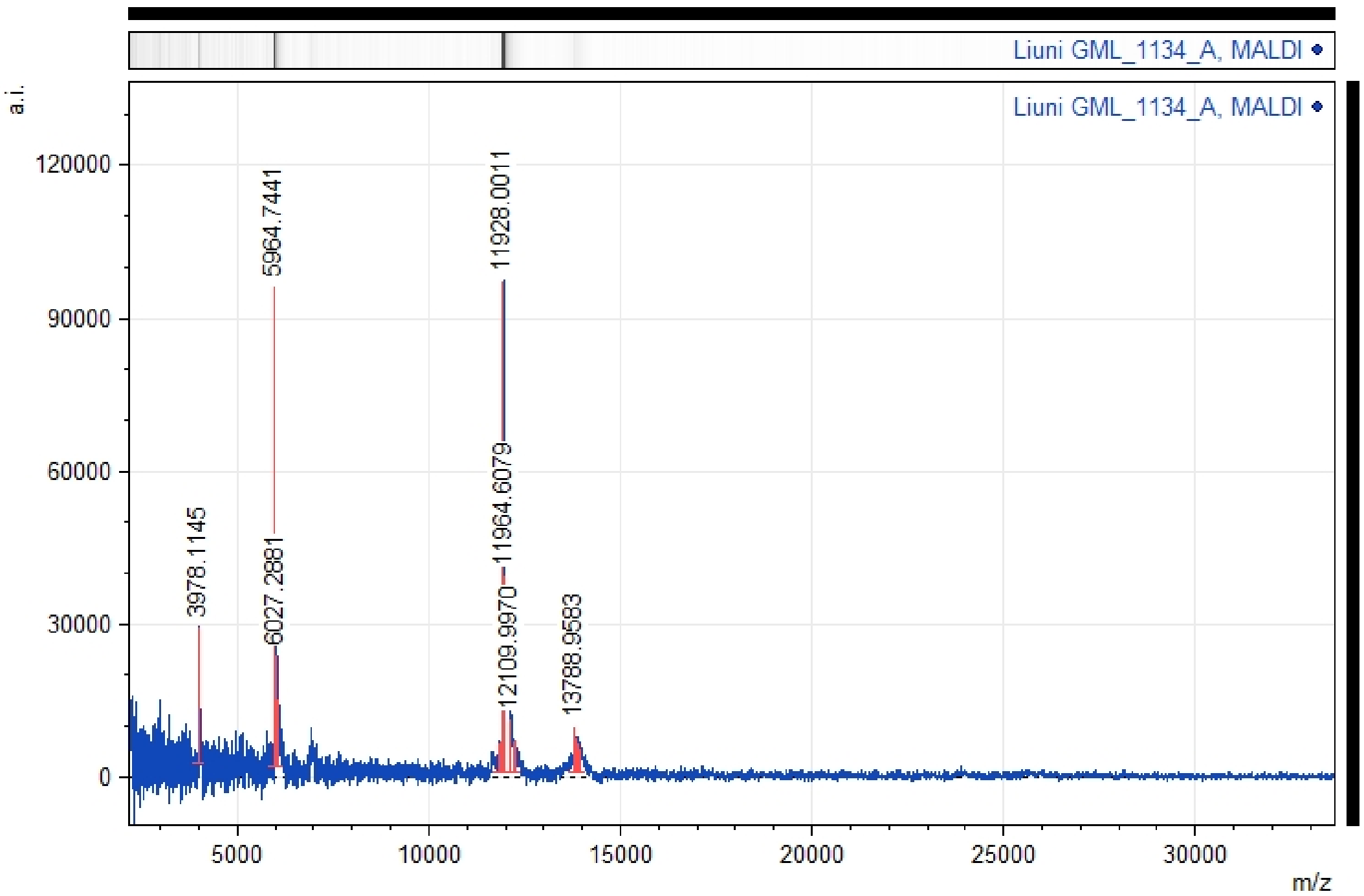

Figure S34. MALDI-TOF spectrum of 5'-(Cy5)-labelled **40DNA_A$^{\mathbf{pegHalo}}$C$^{\mathbf{Bio}}$**; mass calculated for 5'-(Cy5)-labelled **40ON_A$^{\mathbf{pegHalo}}$C$^{\mathbf{Bio}}$**: calculated 13792.08 Da; found 13788.95 Da; Δ = 3.1. The peak at *m/z* = 3978.11 is assigned to template **T40-20** with *z*=3; the peak at *m/z* = 5964.74 is assigned to template **T40-20** with *z*=2; the peak at *m/z* = 11928.00 is assigned to template **T40-20**; the peak at *m/z* = 11964.60 is assigned to template **T40-20** plus K^{+}.

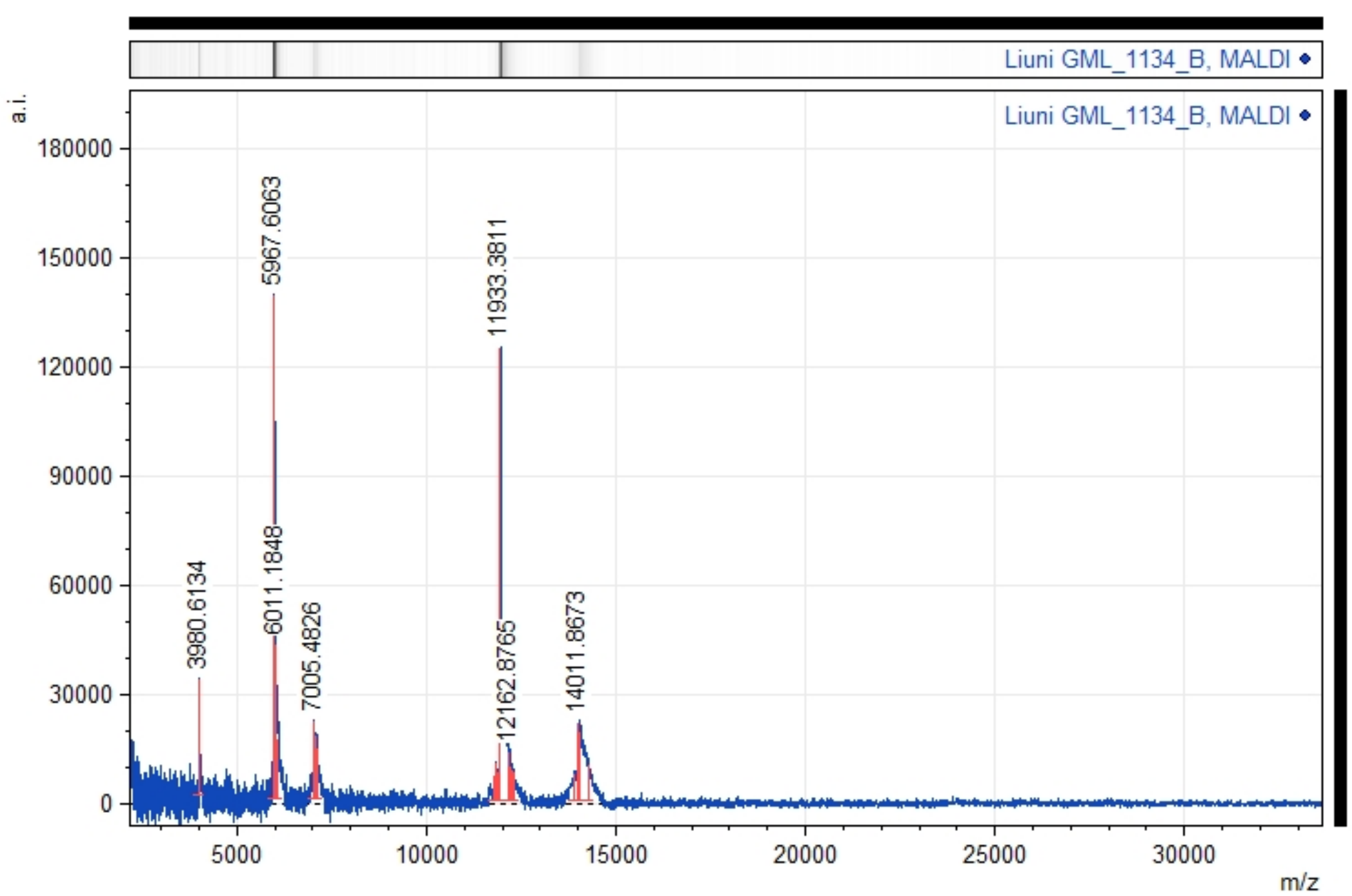

Figure S35. MALDI-TOF spectrum of 5'-(Cy5)-labelled **40DNA_A$^{\mathbf{pegHalo}}$C$^{\mathbf{pegBio}}$**; mass calculated for 5'-(Cy5)-labelled **40ON_A$^{\mathbf{pegHalo}}$C$^{\mathbf{pegBio}}$**: calculated 14005.19 Da; found 14011.86 Da; Δ = 6.7. The peak at *m/z* = 3980.61 is assigned to template **T40-20** with *z*=3; the peak at *m/z* = 5967.60 is assigned to template **T40-20** with *z*=2; the peak at *m/z* = 7005.48 is assigned to **40ON_A$^{\mathbf{pegHalo}}$C$^{\mathbf{pegBio}}$** with *z*=2; the peak at *m/z* = 11933.38 is assigned to template **T40-20**.

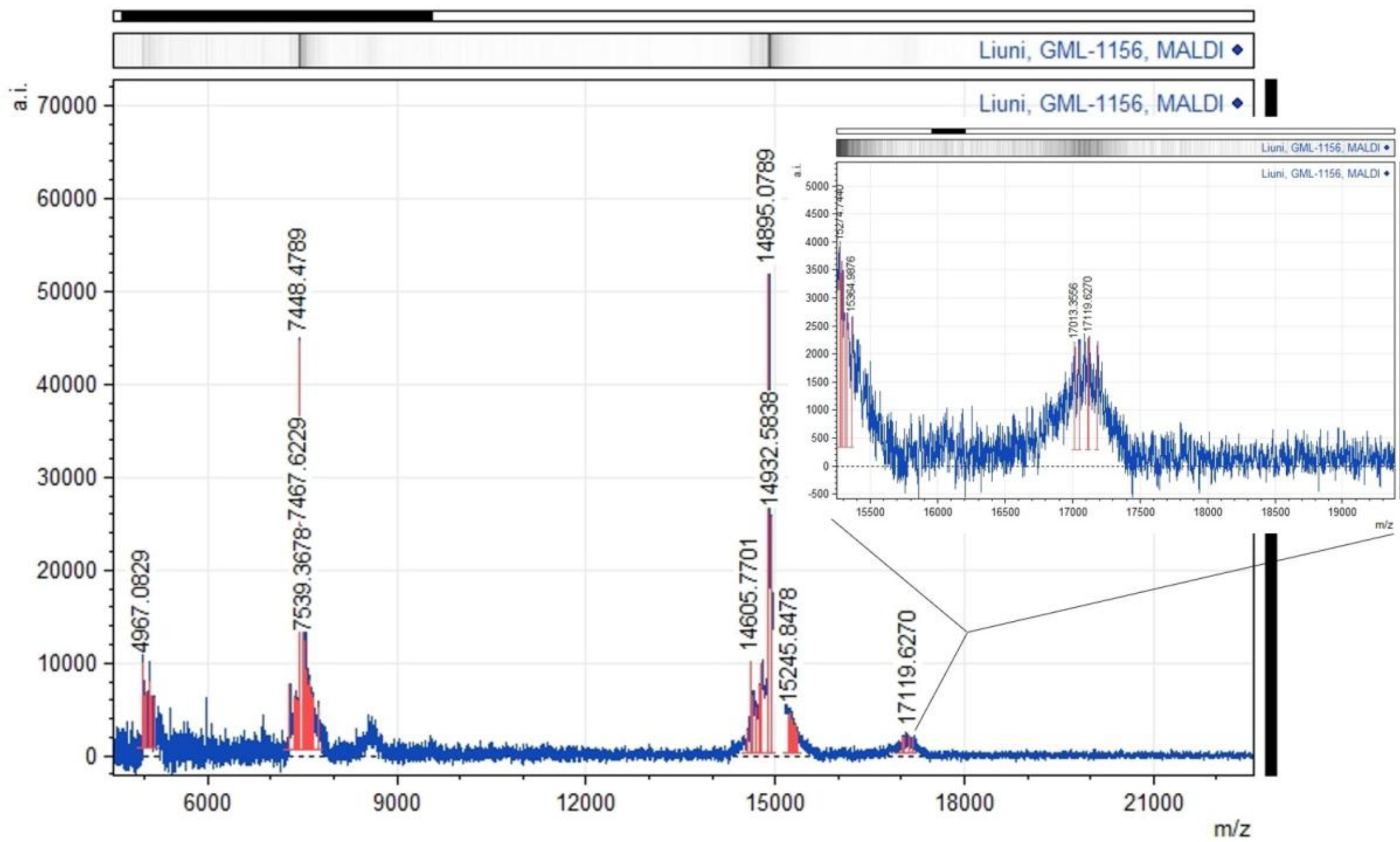

Figure S36. MALDI-TOF spectrum of 5'-(Cy5)-labelled **50DNA_A$^{\mathbf{pegHalo}}$C$^{\mathbf{Bio}}$**; mass calculated for 5'-(Cy5)-labelled **50ON_A$^{\mathbf{pegHalo}}$C$^{\mathbf{Bio}}$**: calculated 17009.18 Da; found 17013.35 Da; Δ = 4.2. The peak at *m/z* = 4967.08 is assigned to template **T50-30** with *z*=3; the peak at *m/z* = 7448.47 is assigned to template **T50-30** with *z*=2; the peak at *m/z* = 14895.07 is assigned to template **T50-30**; the peak at *m/z* = 17119.62 is assigned to 5'-(Cy5)-labelled **50ON_A$^{\mathbf{pegHalo}}$C$^{\mathbf{Bio}}$** plus 3K^+.

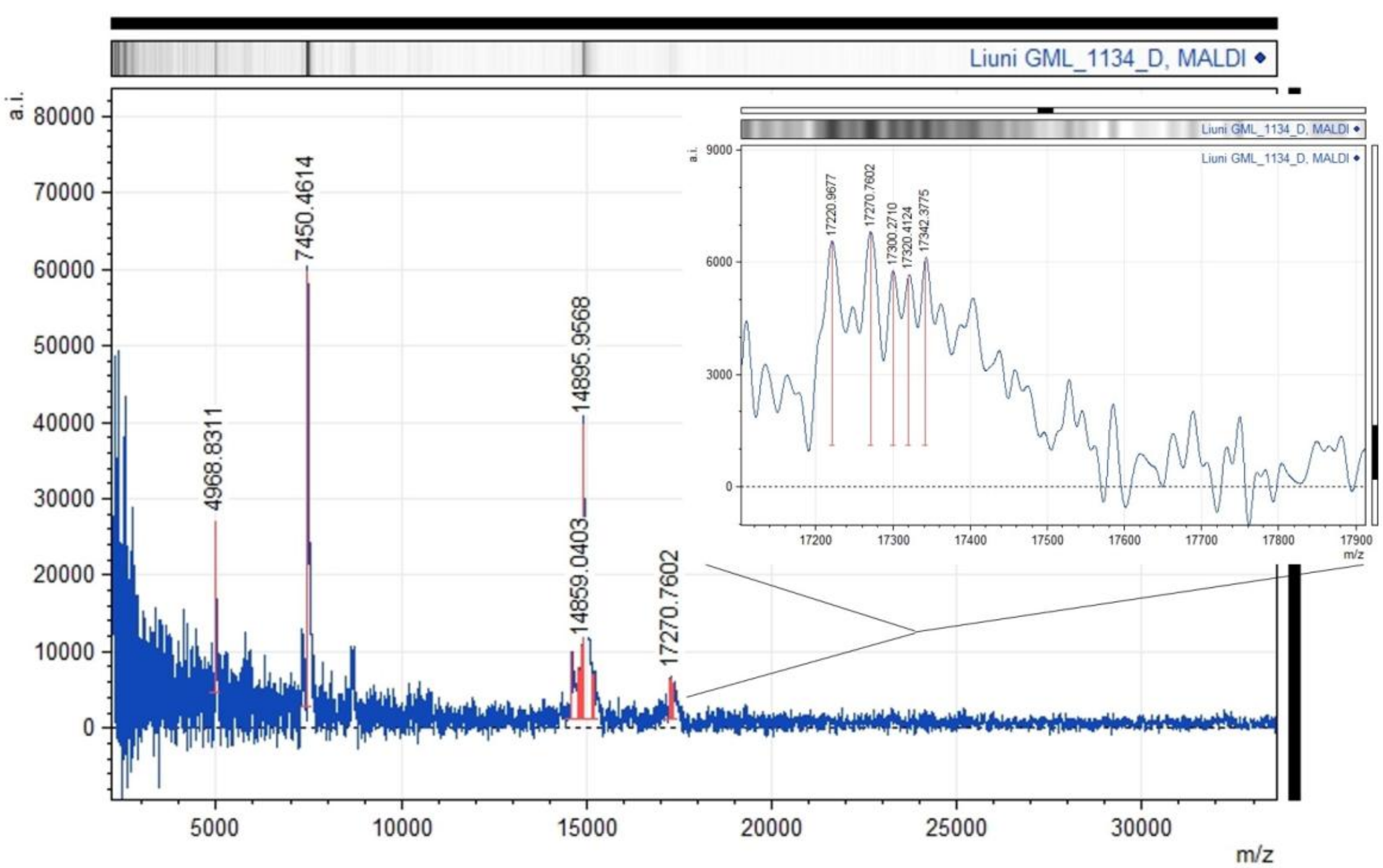

Figure S37. MALDI-TOF spectrum of 5'-(Cy5)-labelled **50DNA_A$^{\mathbf{pegHalo}}$C$^{\mathbf{pegBio}}$**; mass calculated for 5'-(Cy5)-labelled **50ON_A$^{\mathbf{pegHalo}}$C$^{\mathbf{pegBio}}$**: calculated 17222.29 Da; found 17220.96 Da; Δ = 1.3. The peak at *m/z* = 4968.83 is assigned to template **T50-30** with *z*=3; the peak at *m/z* = 7450.46 is assigned to template **T50-30** with *z*=2; the peak at *m/z* = 14895.95 is assigned to template **T50-30**.

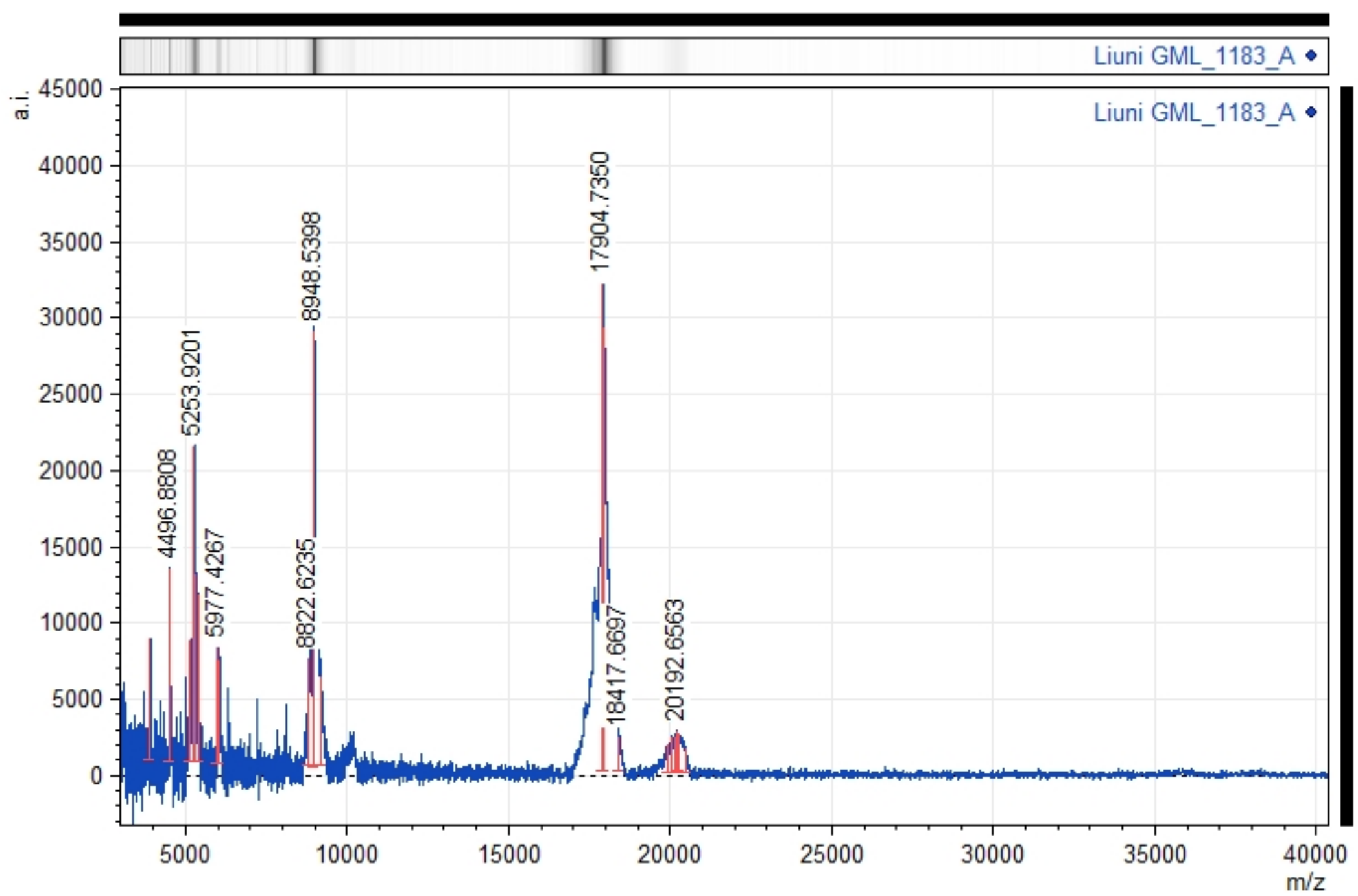

Figure S38. MALDI-TOF spectrum of 5'-(Cy5)-labelled **60DNA_A$^{\mathbf{pegHalo}}$C$^{\mathbf{Bio}}$**; mass calculated for 5'-(Cy5)-labelled **60ON_A$^{\mathbf{pegHalo}}$C$^{\mathbf{Bio}}$**: calculated 20200.08 Da; found 20192.65 Da; Δ = 7.4. The peak at *m/z* = 4496.88 is assigned to template **T60-40** with *z*=4; the peak at *m/z* = 8948.53 is assigned to template **T60-40** with *z*=2; the peak at *m/z* = 17904.73 is assigned to template **T60-40**.

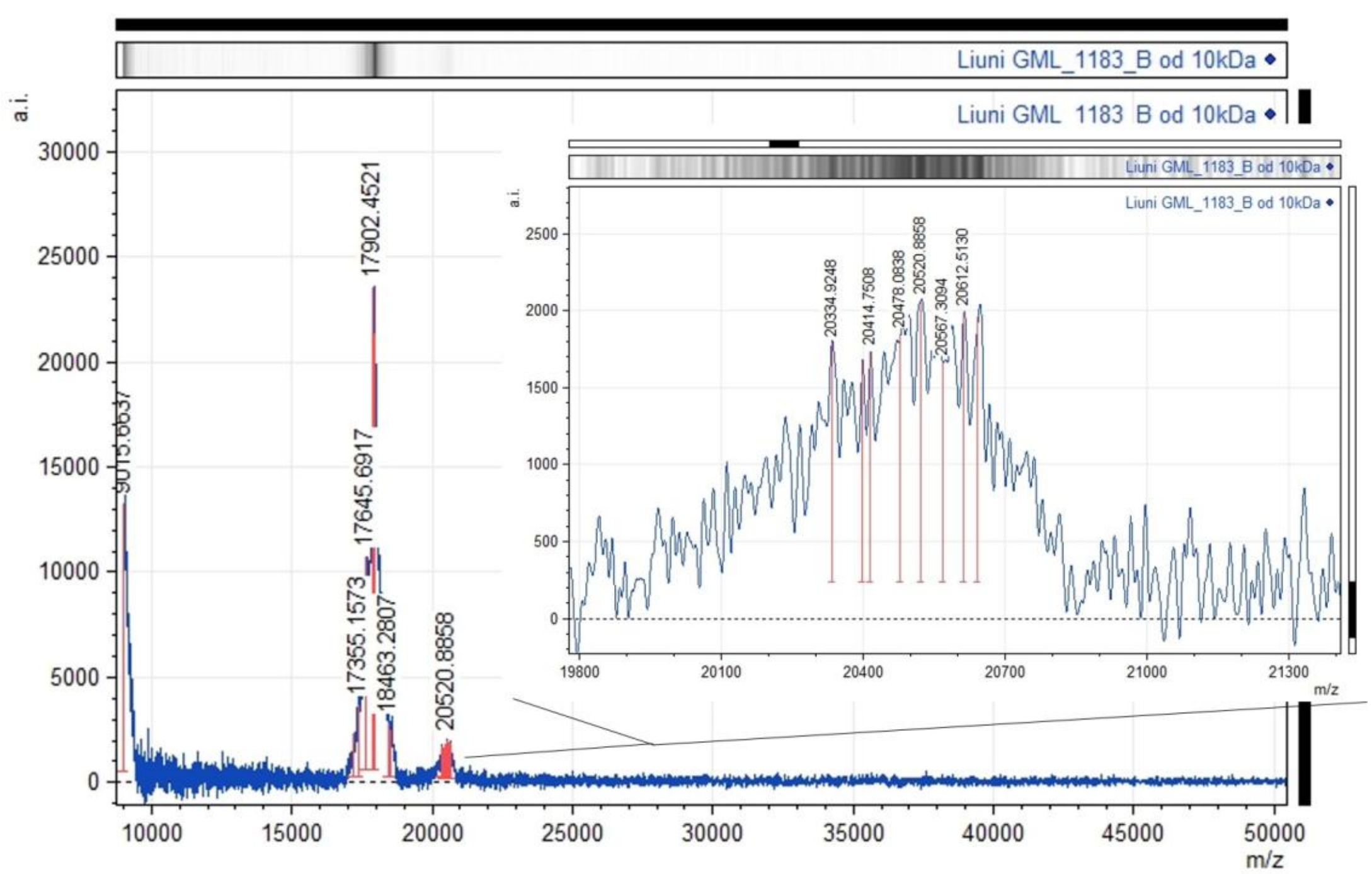

Figure S39. MALDI-TOF spectrum of 5'-(Cy5)-labelled **60DNA_A$^{\mathbf{pegHalo}}$C$^{\mathbf{pegBio}}$**; mass calculated for 5'-(Cy5)-labelled **60ON_A$^{\mathbf{pegHalo}}$C$^{\mathbf{pegBio}}$**: calculated 20413.19 Da; found 20414.75 Da; Δ = 1.6. The peak at *m/z* = 17902.42 is assigned to template **T60-40**.

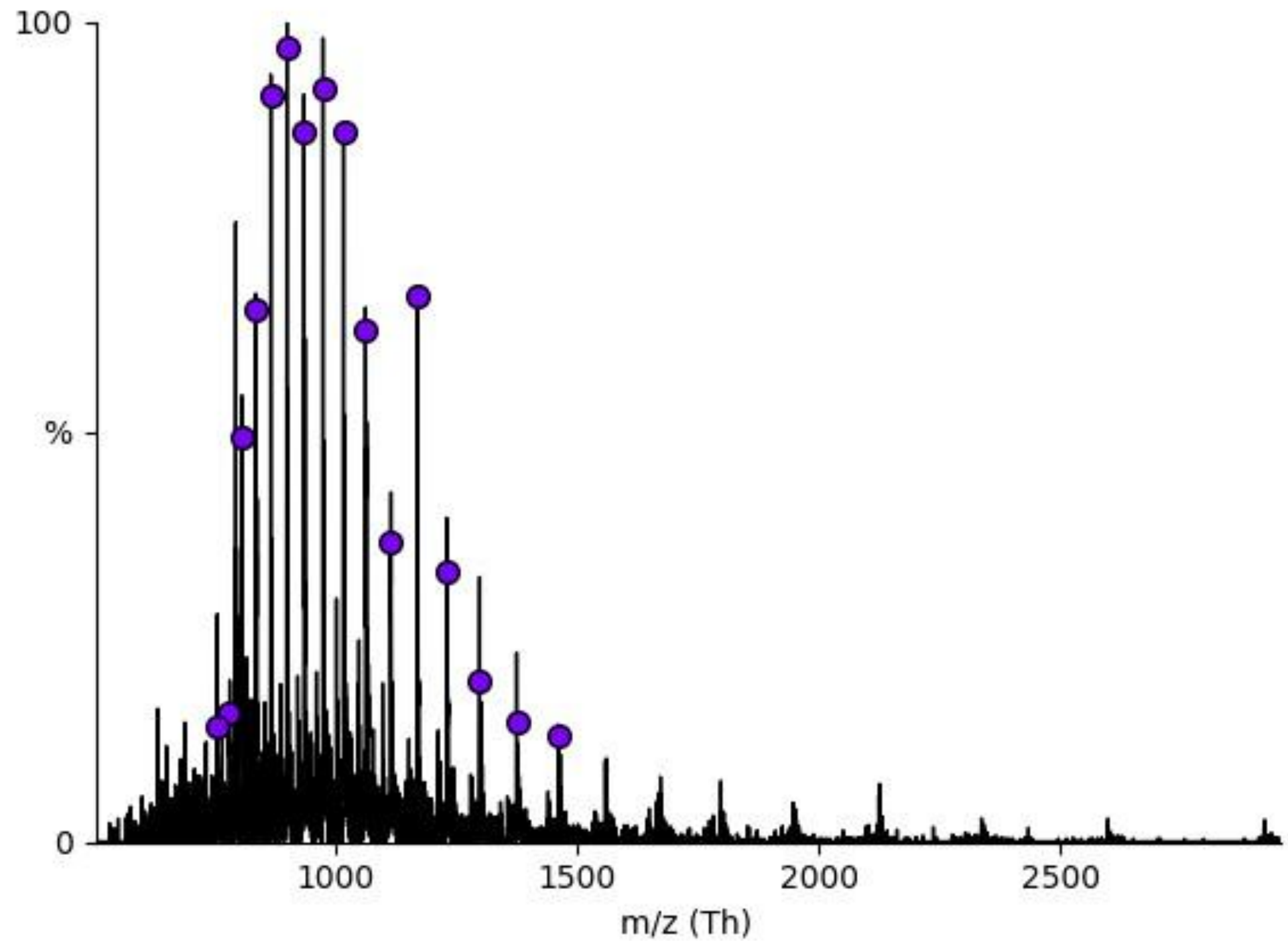

Figure S40A. Raw MS spectrum of 5'-(Cy5)-labelled **70ON_A^{pegHalo}C^{Bio}**.

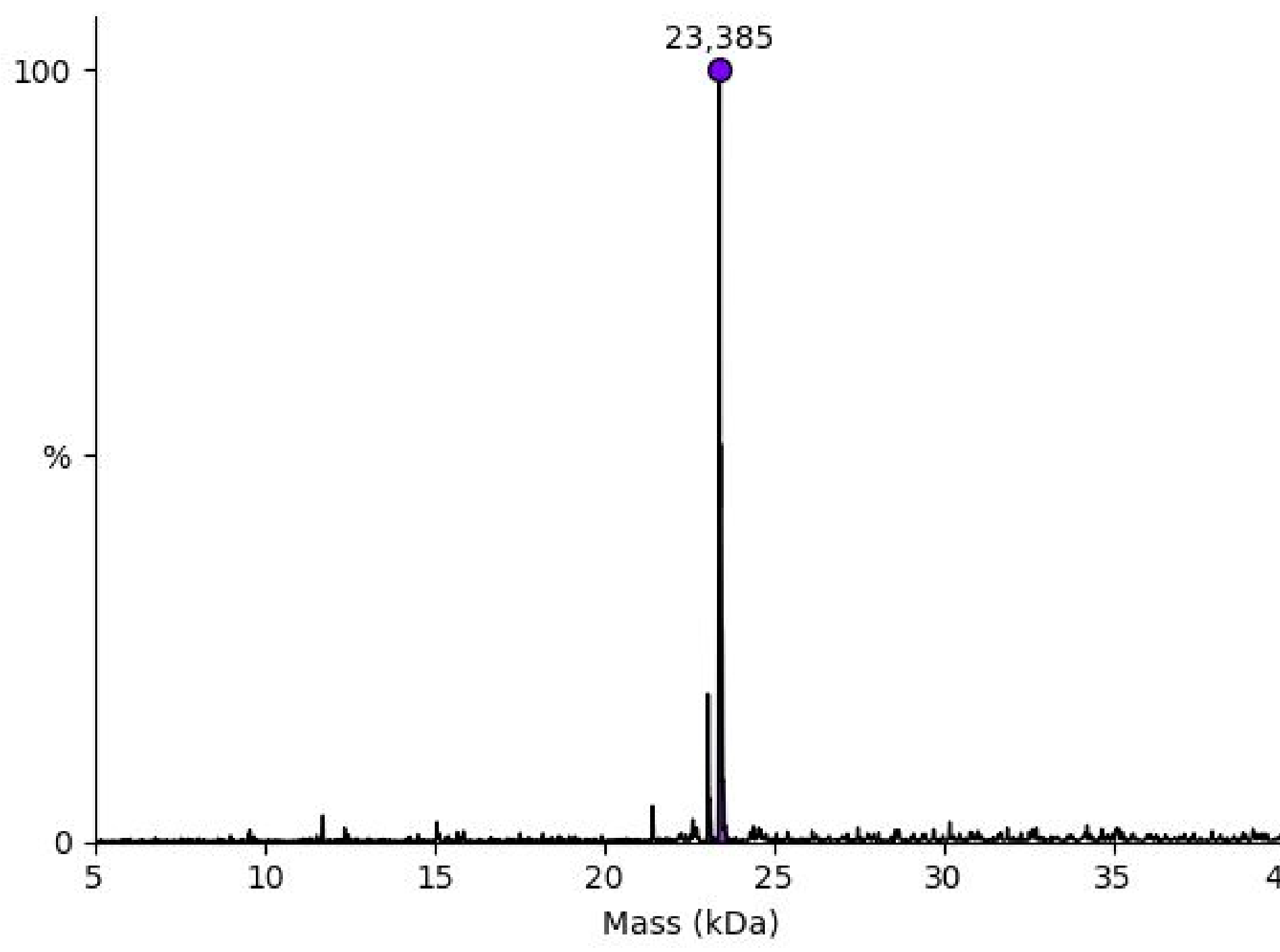

Figure S40B. Deconvoluted MS spectrum of 5'-(Cy5)-labelled **70ON_A^{pegHalo}C^{Bio}**: calculated 23392.08 Da; found 23385.00 Da; Δ = 7.1.

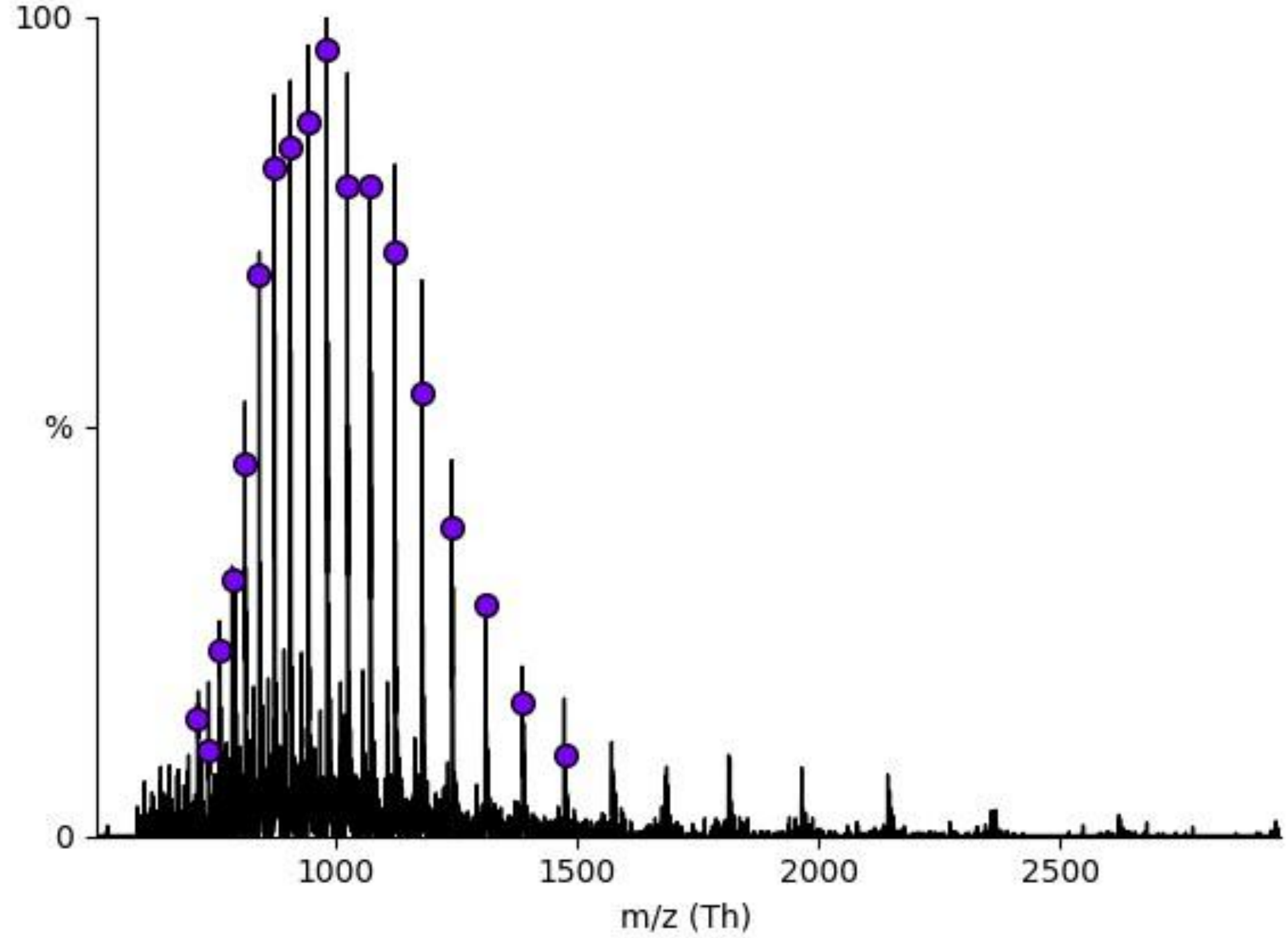

Figure S41A. Raw MS spectrum of 5'-(Cy5)-labelled **70ON_A^{pegHalo}C^{pegBio}**.

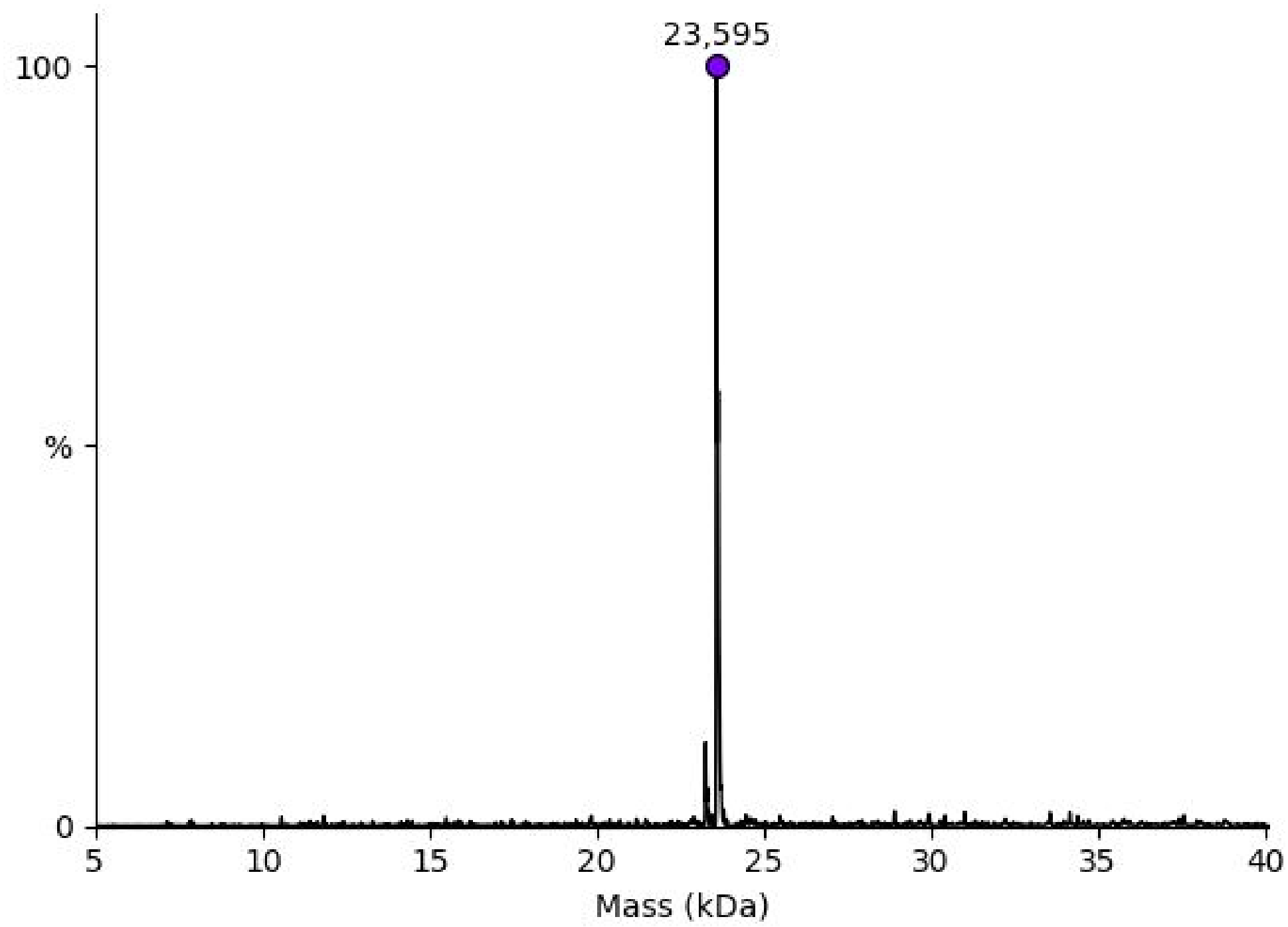

Figure S41B. Deconvoluted MS spectrum of 5'-(Cy5)-labelled **70ON_A^{pegHalo}C^{pegBio}**: calculated 23605.19 Da; found 23595.00 Da; Δ = 10.2.

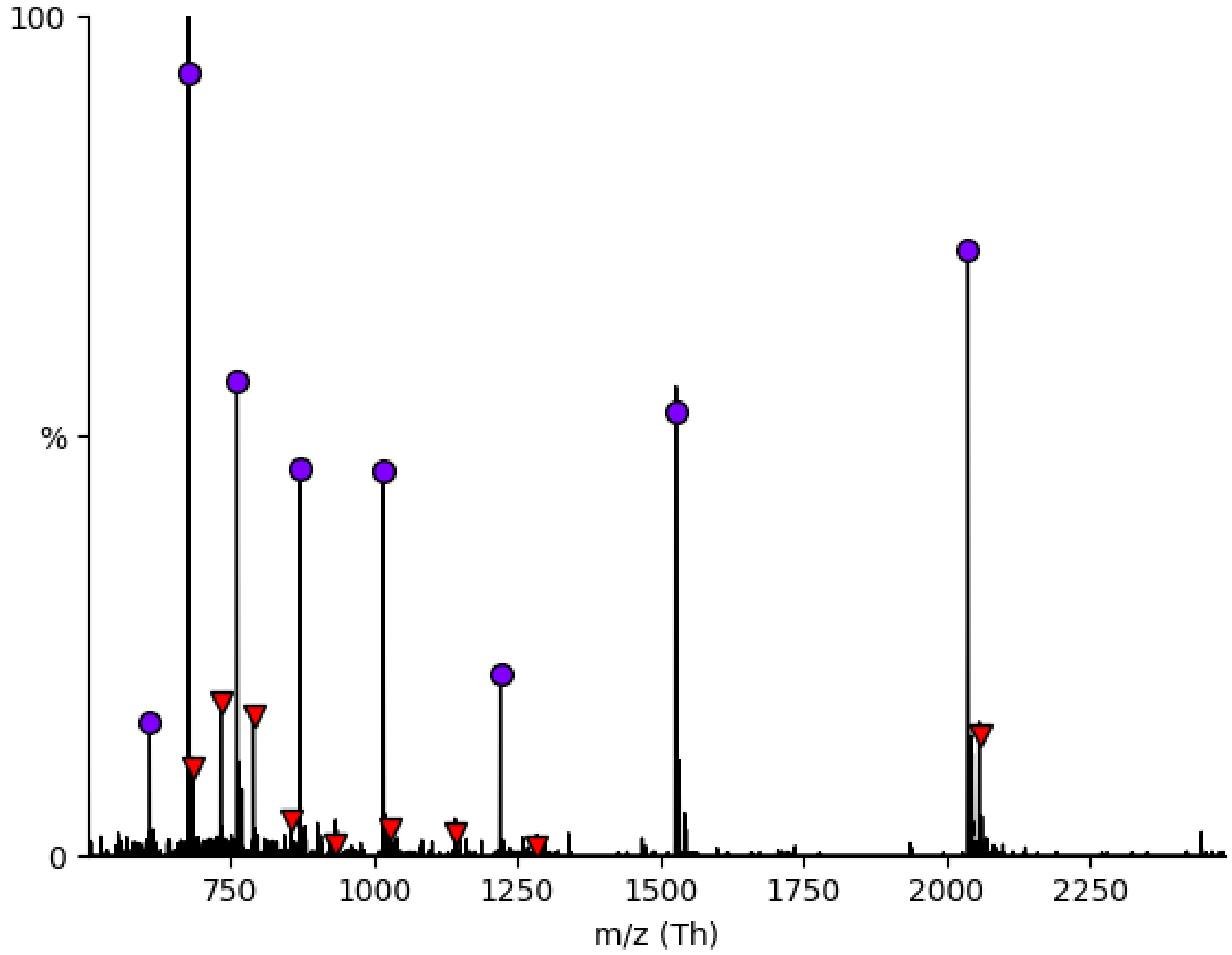

Figure S42A. Raw MS spectrum of 5'-(Cy5)-labelled **17ON_A$^{\mathbf{pegHalo}}$**.

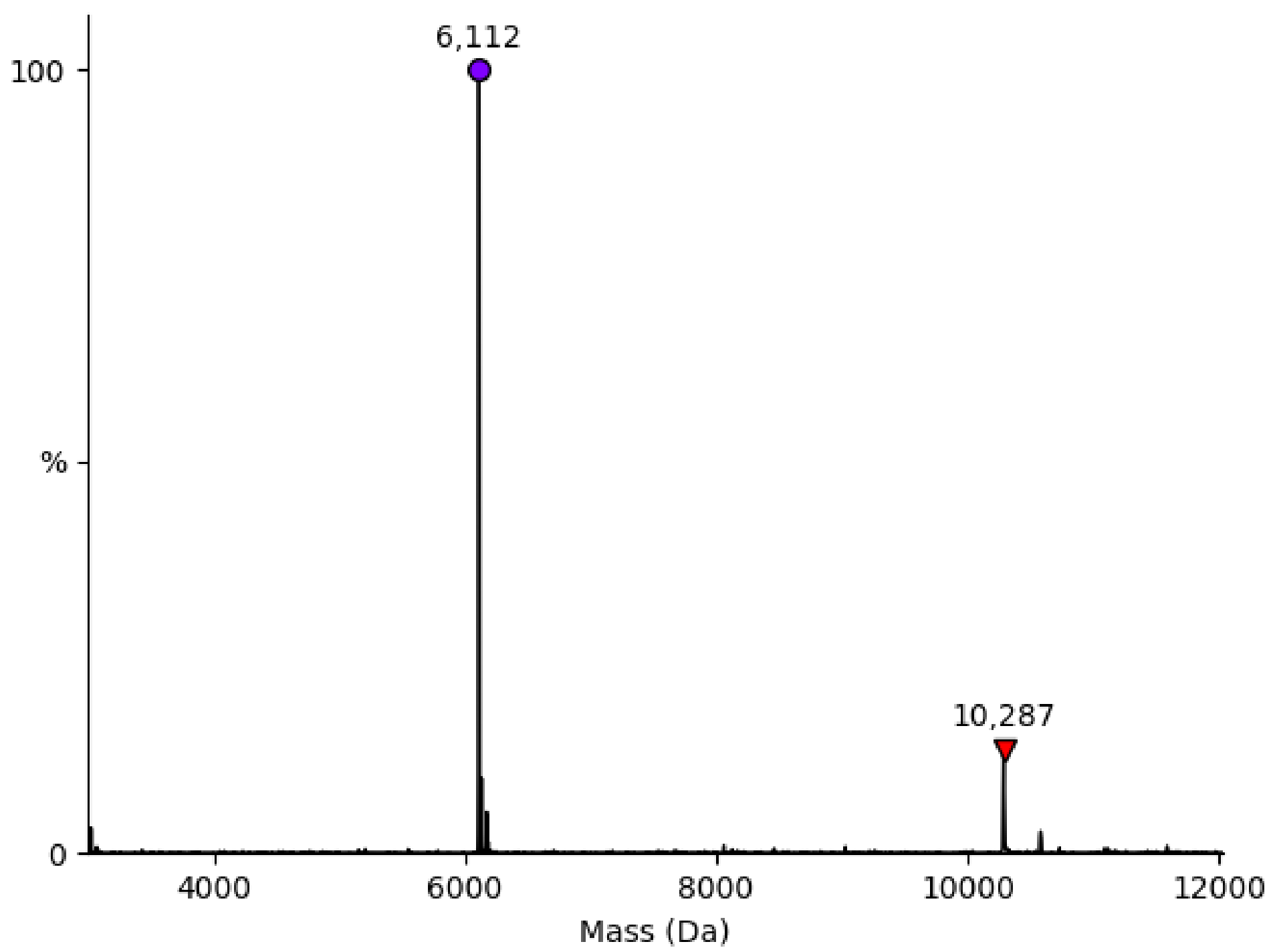

Figure S42B. Deconvoluted MS spectrum of 5'-(Cy5)-labelled **17ON_A$^{\mathbf{pegHalo}}$**: calculated 6114.26 Da; found 6112.00 Da; Δ = 2. The peak at *m/z* = 10287 is assigned to 5'-dual biotinylated template **Prb4basII-bio**.

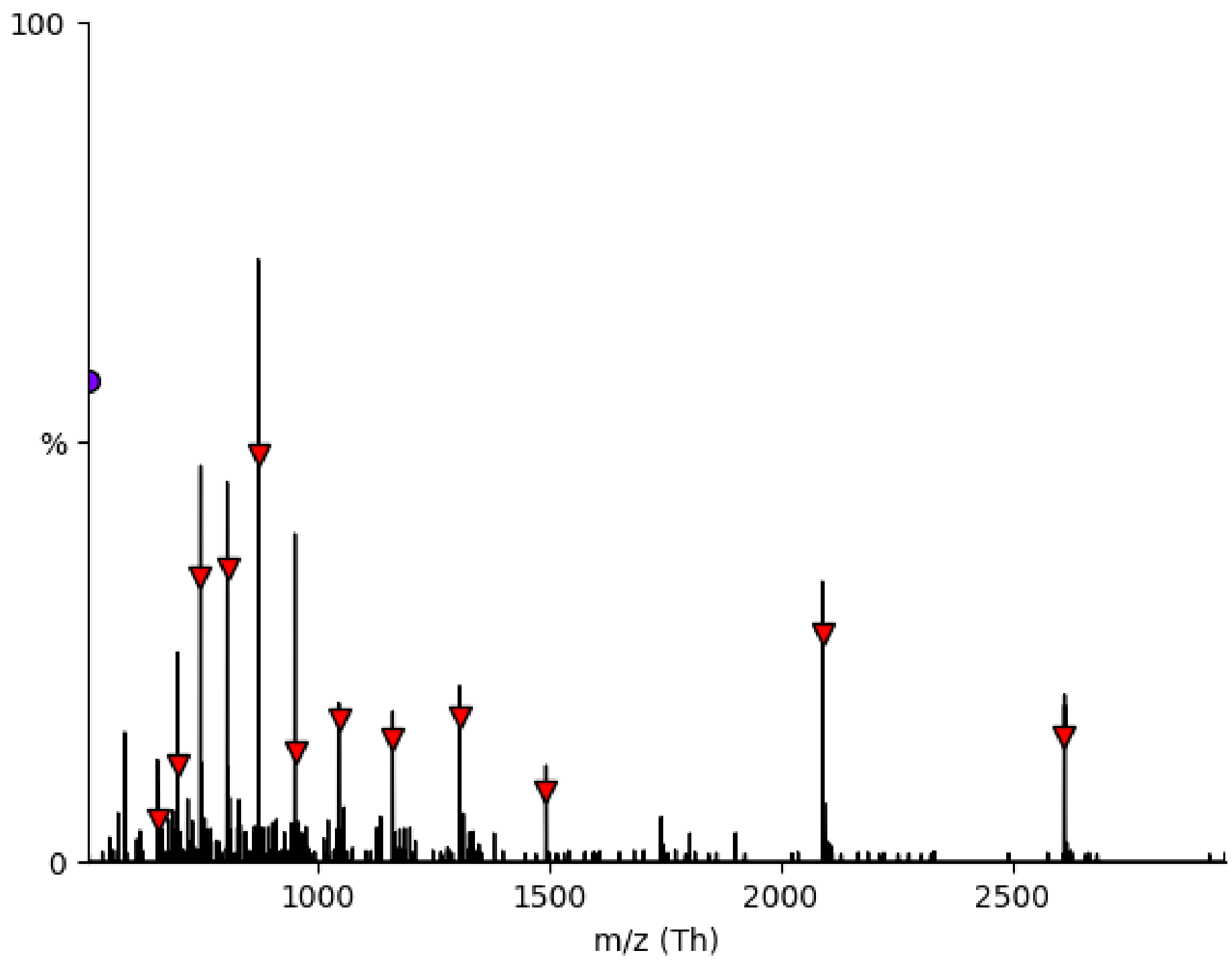

Figure S43A. Raw MS spectrum of 5'-(Cy5)-labelled **31ON_A^{pegHalo}**.

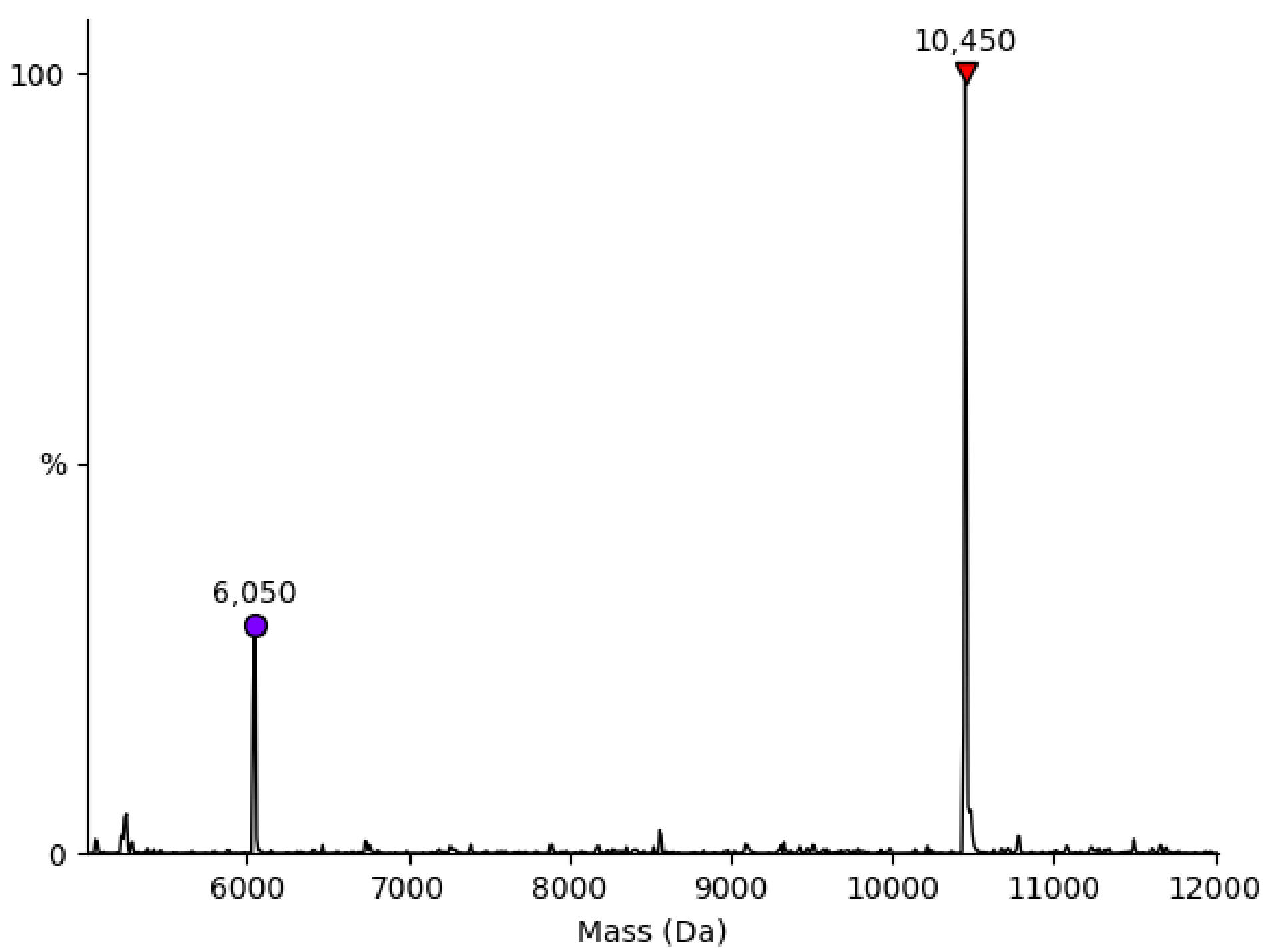

Figure S43B. Deconvoluted MS spectrum of 5'-(Cy5)-labelled **31ON_A^{pegHalo}**: calculated 10455.06 Da; found 10450.00 Da; Δ = 5. The peak at *m/z* = 6050.00 is assigned to a shorter oligonucleotide not fully extended after PEX reaction.

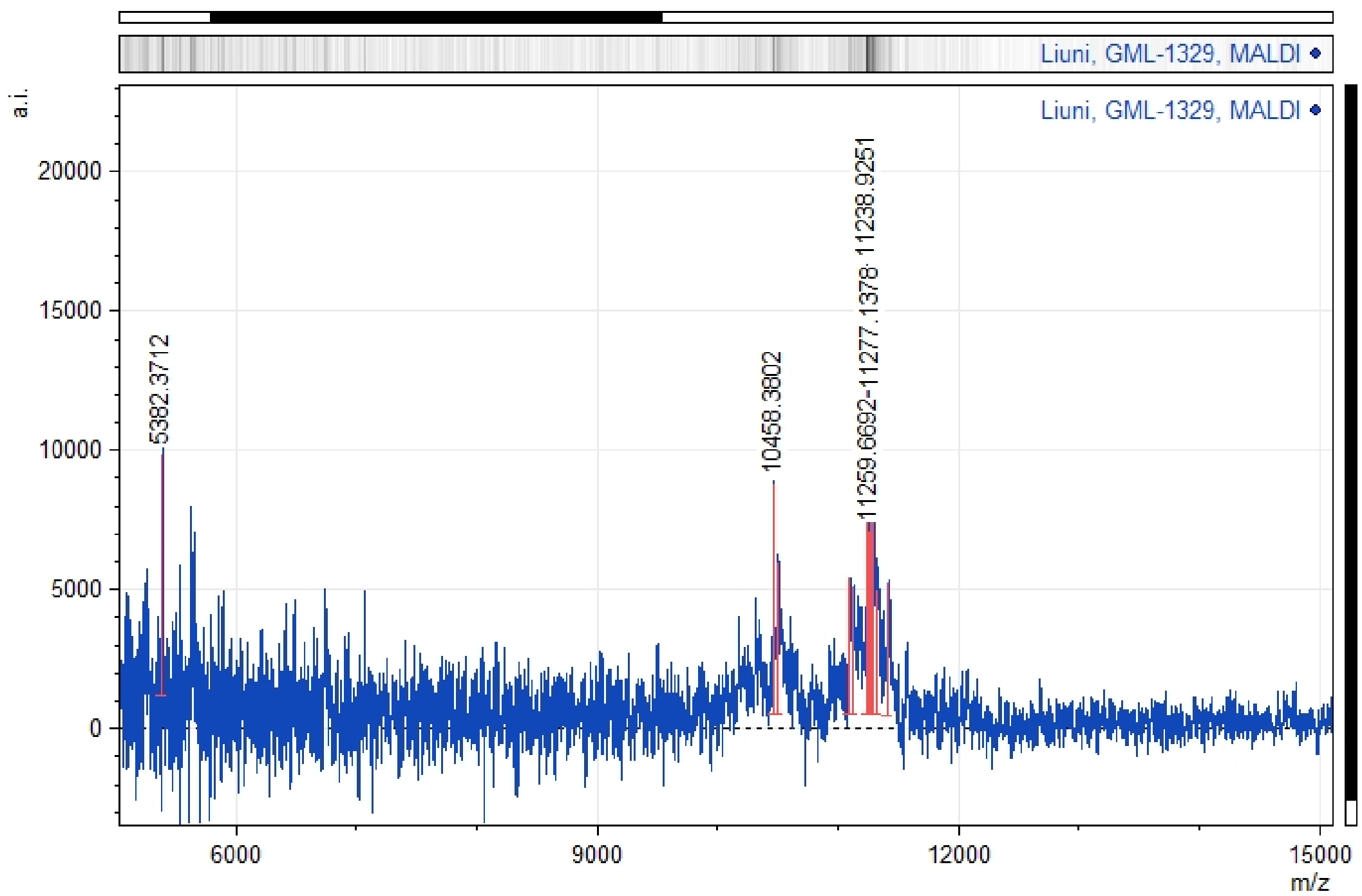

Figure S44. MALDI-TOF spectrum of 5'-(Cy5)-labelled **32ON_A^{pegHalo}C^{pegBio}**: calculated 11236.49 Da; found 11238.92 Da; Δ = 2.4. The peak at *m/z* = 10458.38 is assigned to 5'-(Cy5)-labelled **31ON_A^{pegHalo}**; the peak at *m/z* = 11277.13 is assigned to 5'-(Cy5)-labelled **32ON_A^{pegHalo}C^{pegBio}** plus K^+.

4. Copies of NMR spectra

^1H, ^{13}C spectra in $CDCl_3$ of **2**

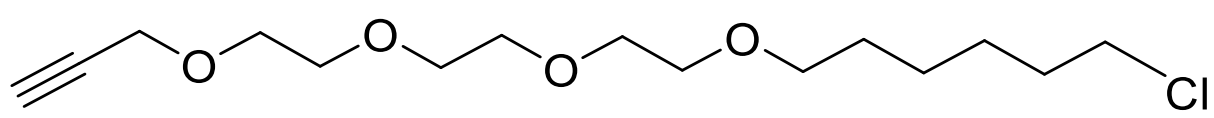

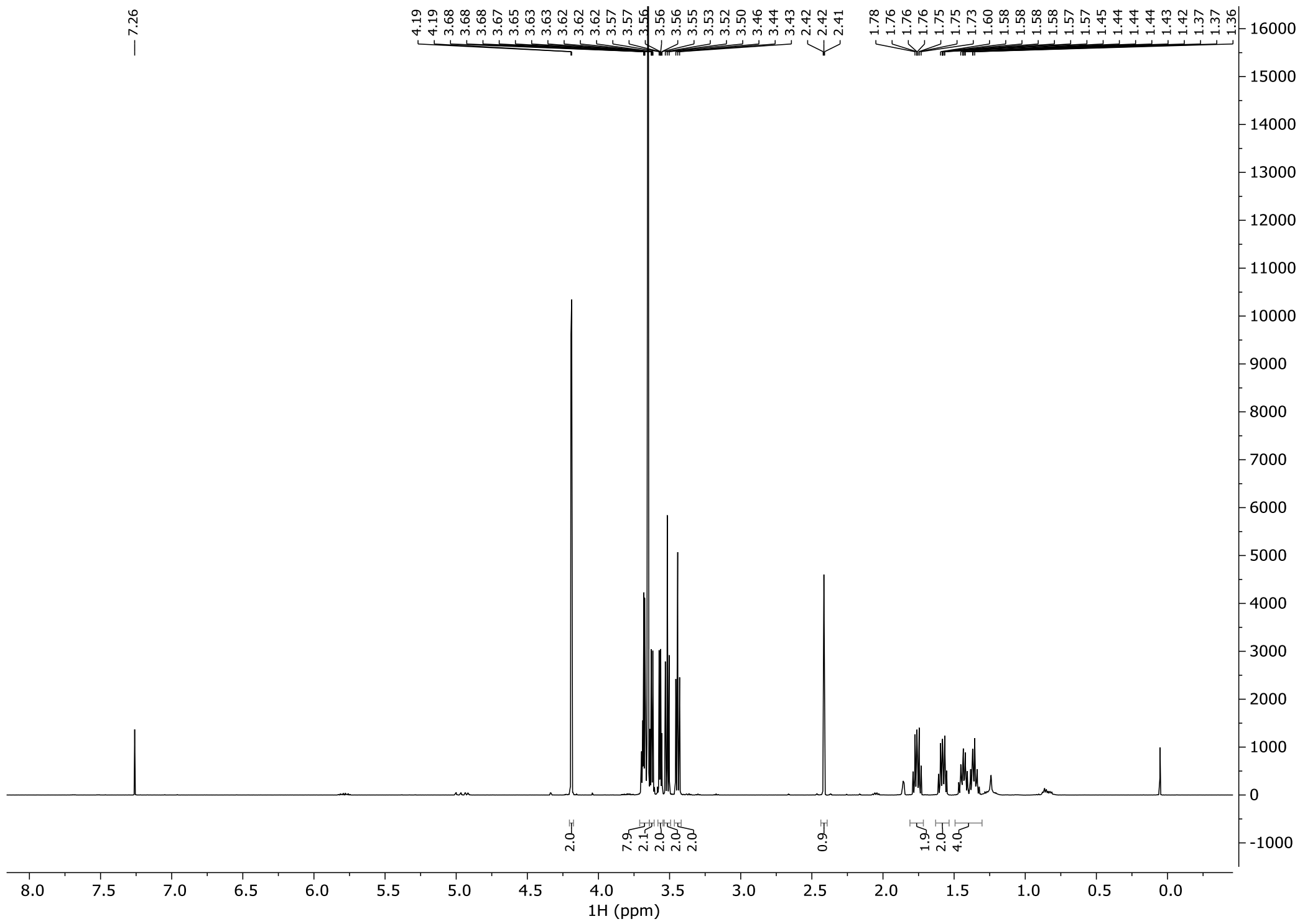

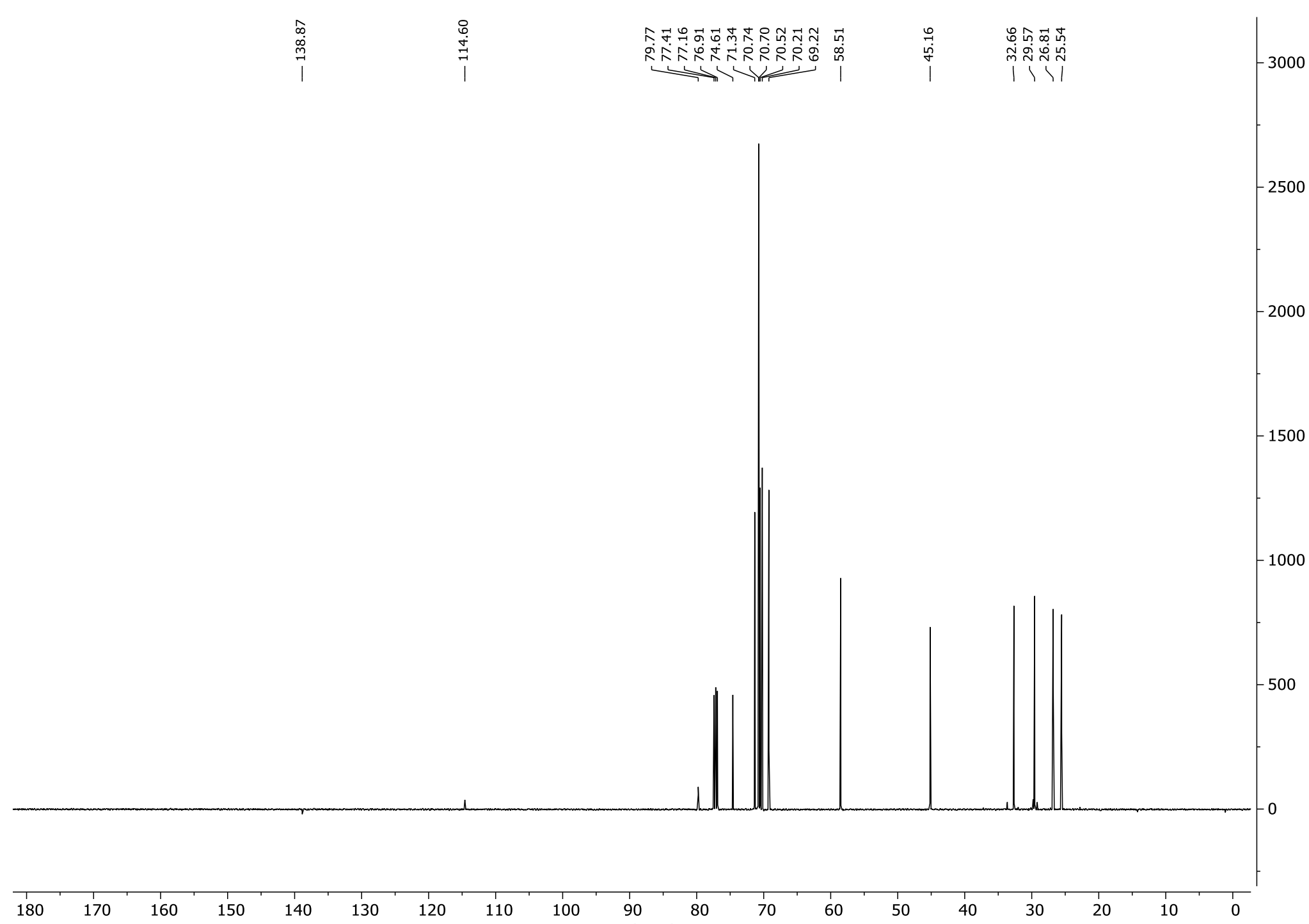

^{1}H, ^{13}C, ^{31}P{^{1}H} spectra in D_2O of **dA[pegHalo]TP**

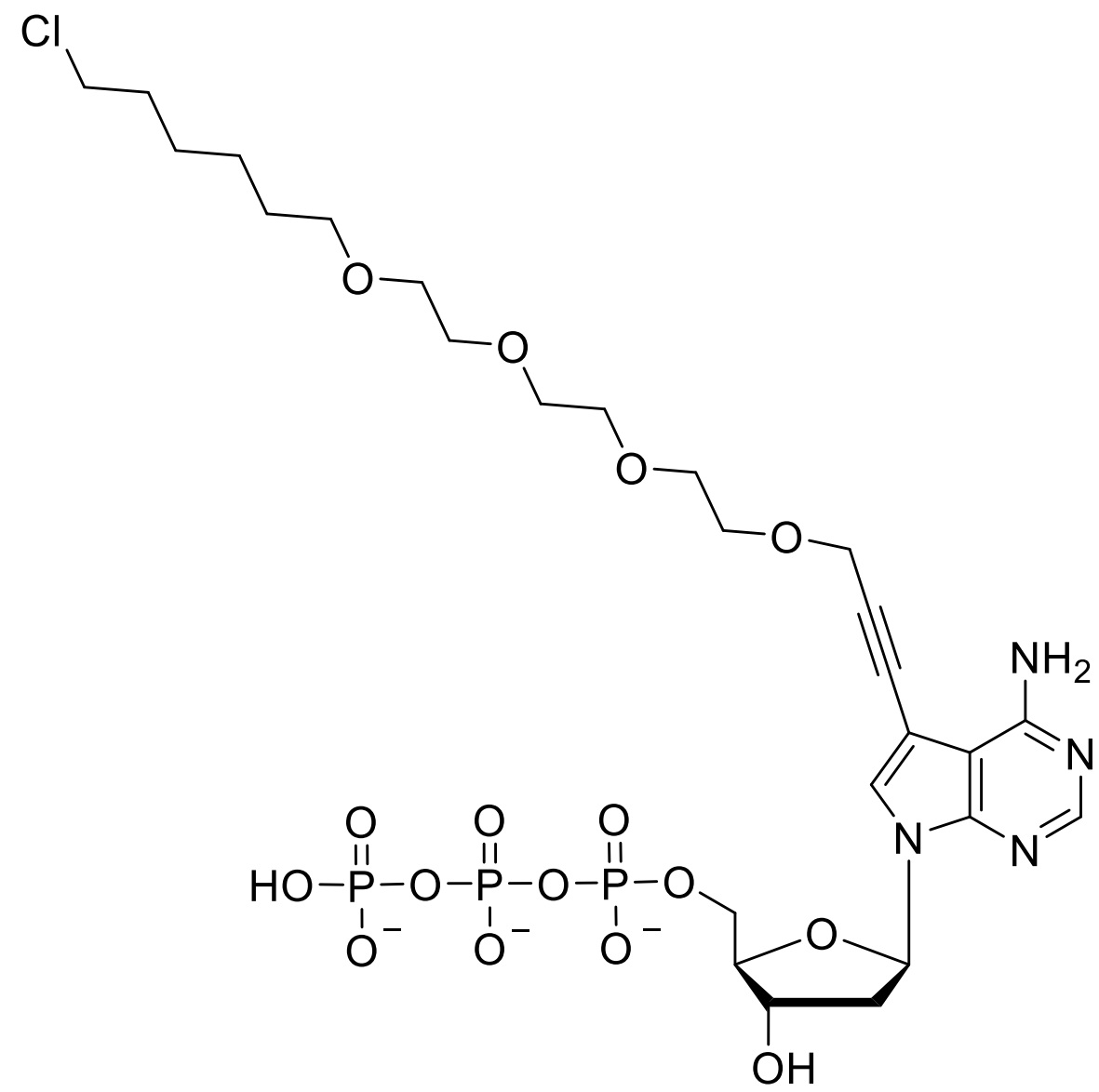

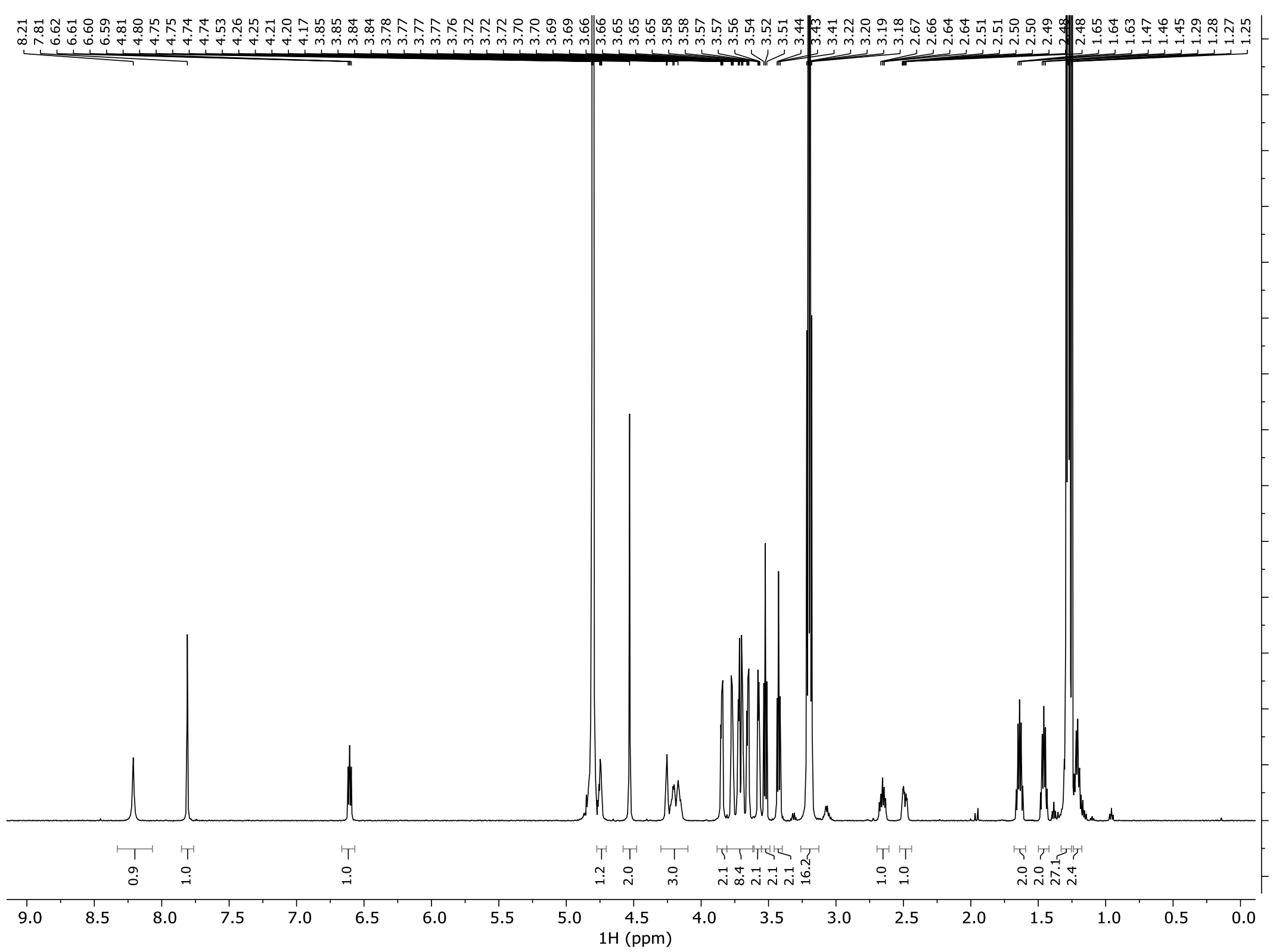

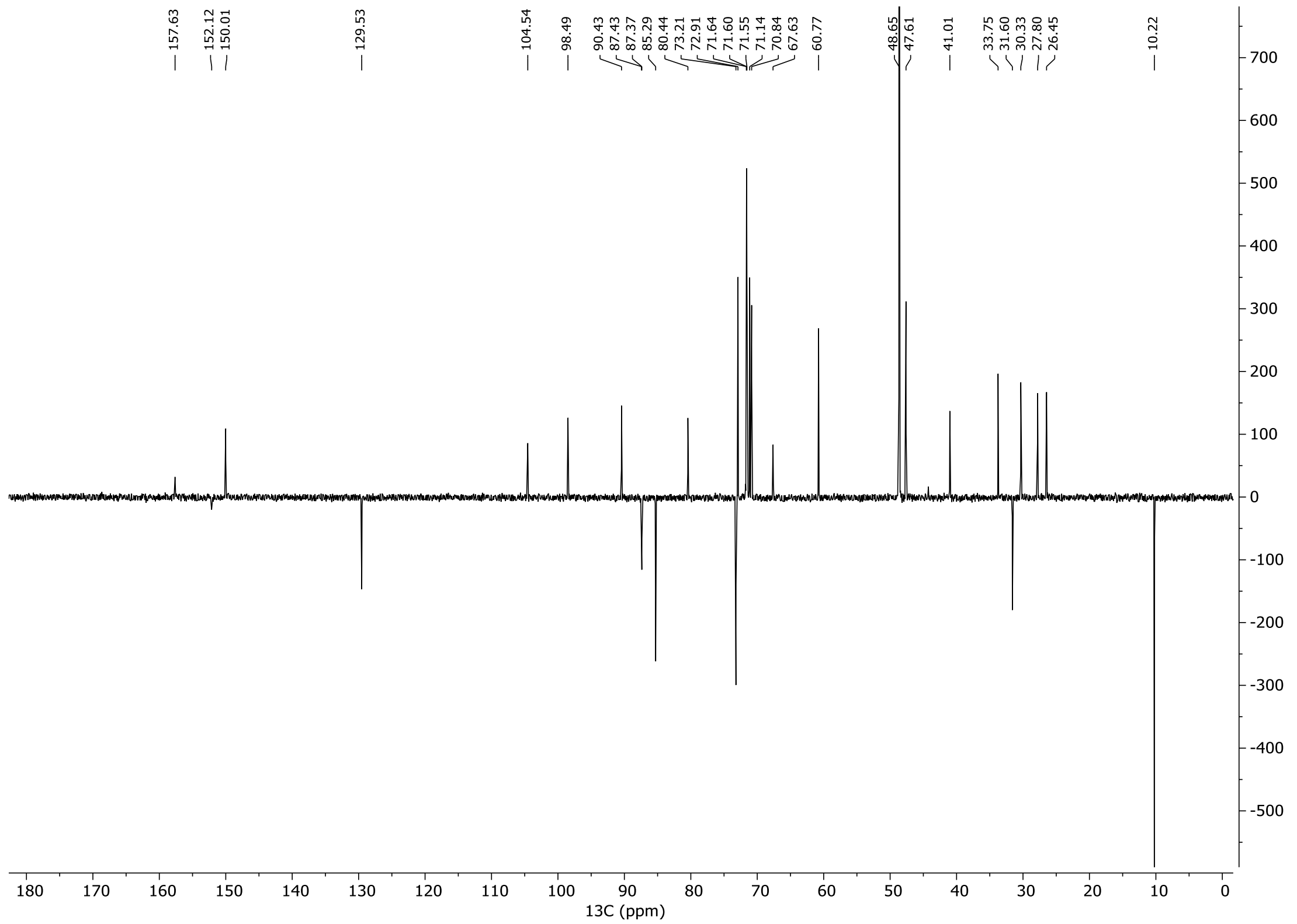
157.63
152.12
150.01
129.53
104.54
98.49
90.43
87.43
87.37
85.29
80.44
73.21
72.91
71.64
71.60
71.55
71.14
70.84
67.63
60.77
48.65
47.61
41.01
33.75
31.60
30.33
27.80
26.45
10.22
13C (ppm)

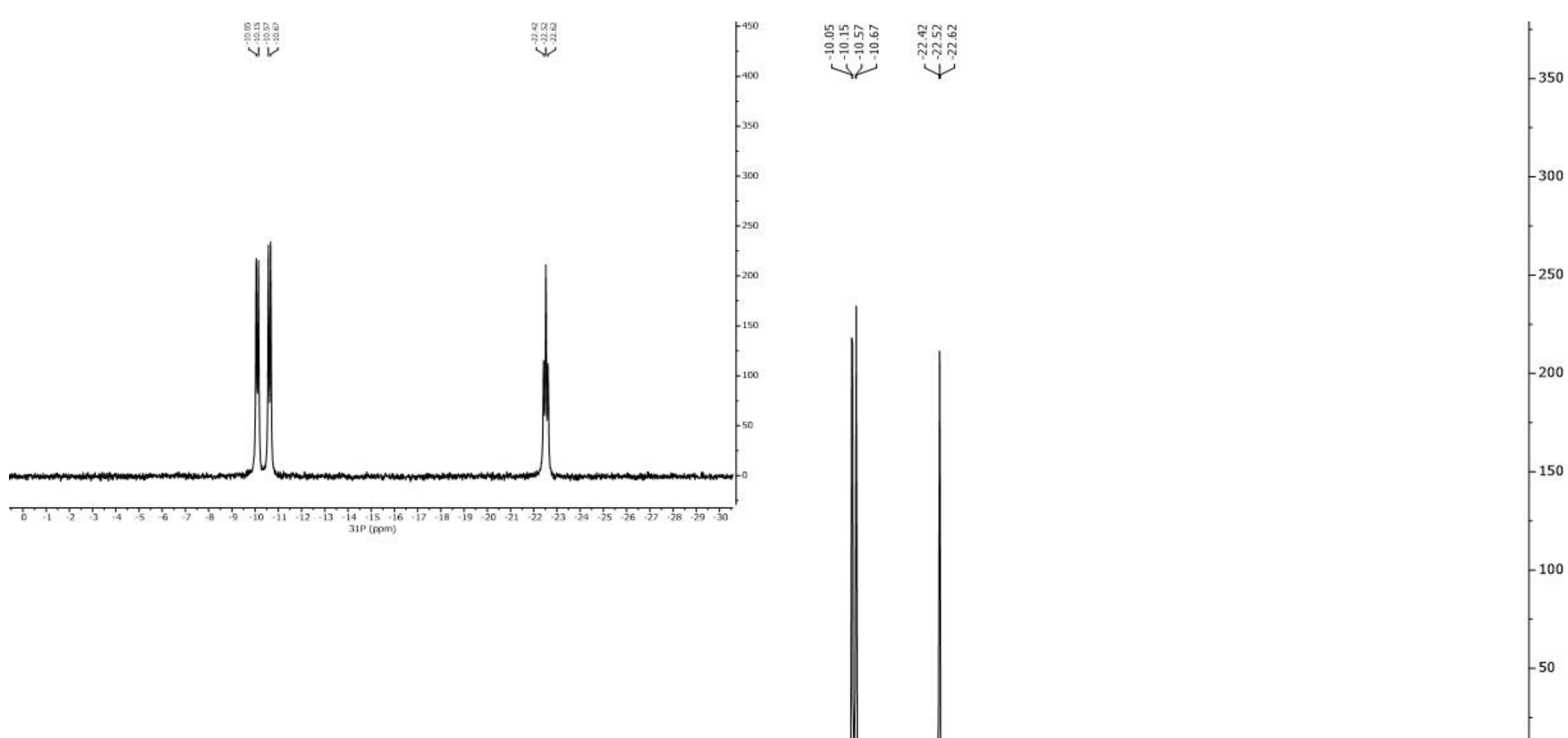
-10.05
-10.15
-10.57
-10.67
-22.42
-22.52
-22.62
31P (ppm)

^{1}H, ^{13}C, ^{31}P{^{1}H} spectra in D_2O of **dC^Bio^TP**

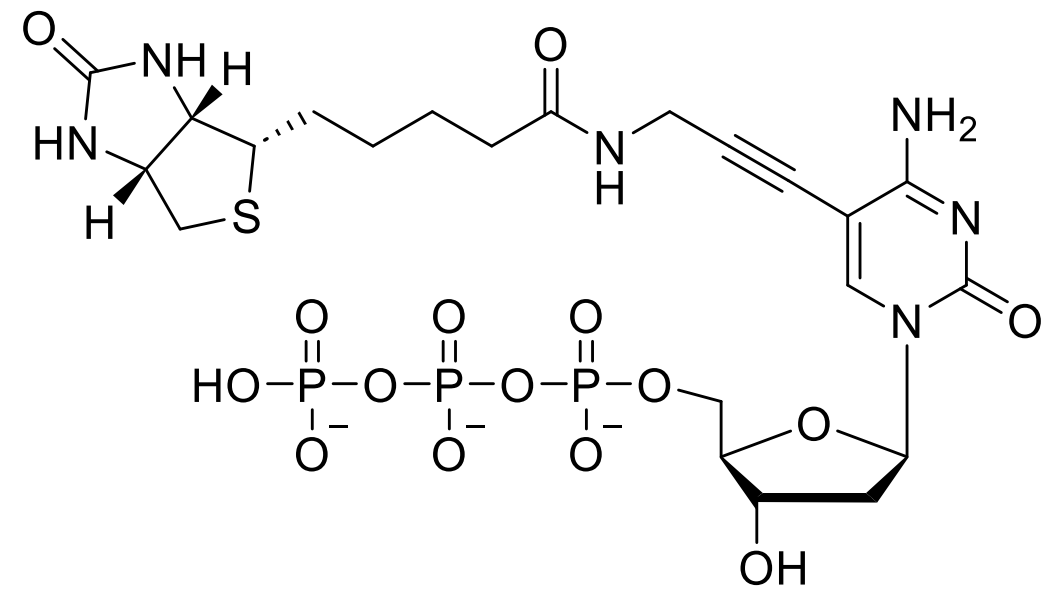

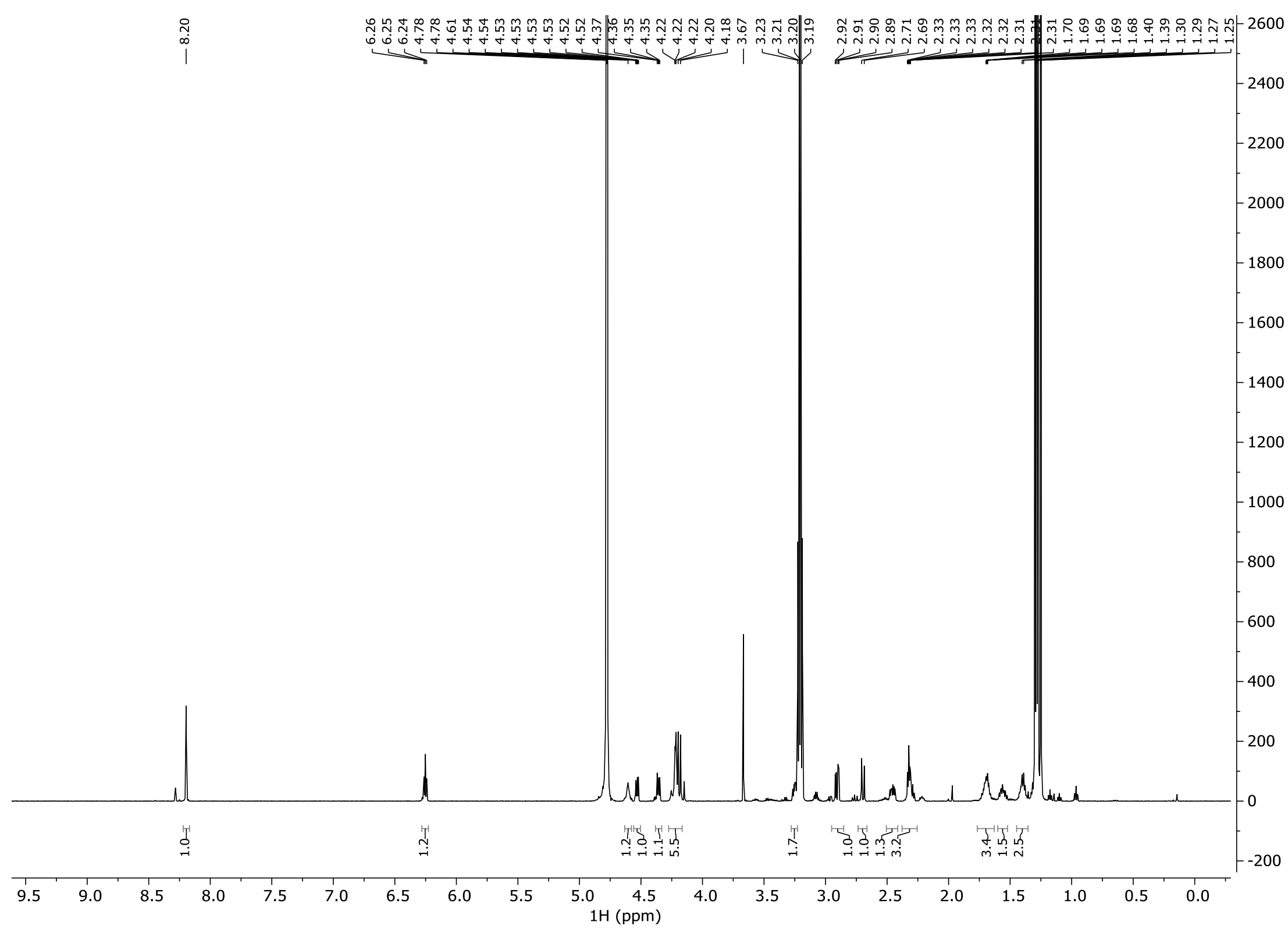

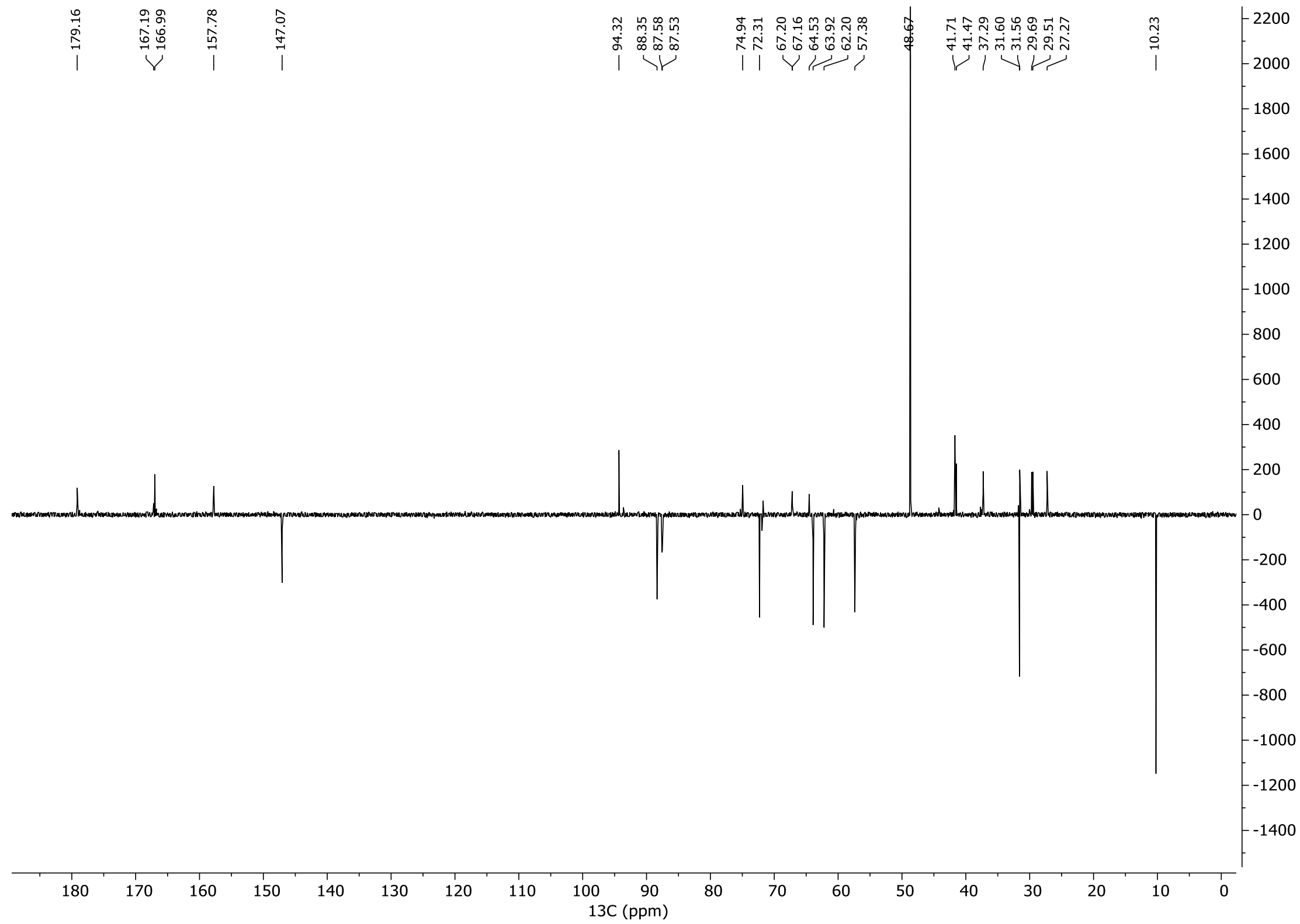
179.16
167.19
166.99
157.78
147.07
94.32
88.35
87.58
87.53
74.94
72.31
67.20
67.16
64.53
63.92
62.20
57.38
48.67
41.71
41.47
37.29
31.60
31.56
29.69
29.51
27.27
10.23
13C (ppm)

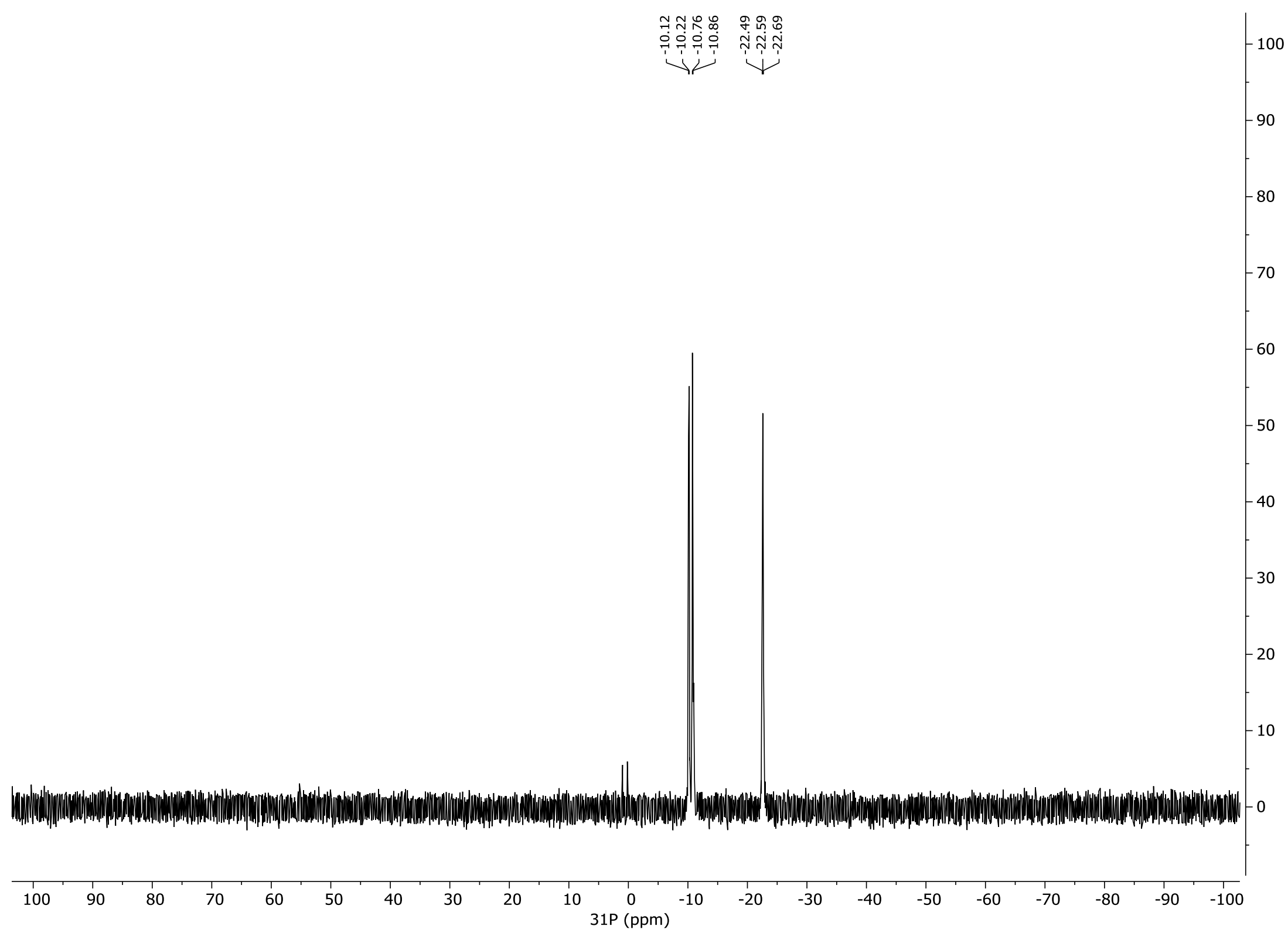
-10.12
-10.22
-10.76
-10.86
-22.49
-22.59
-22.69
31P (ppm)

^{1}H, ^{13}C, $^{31}P\{^{1}H\}$ spectra in D_2O of **dC^{pegBio}TP**

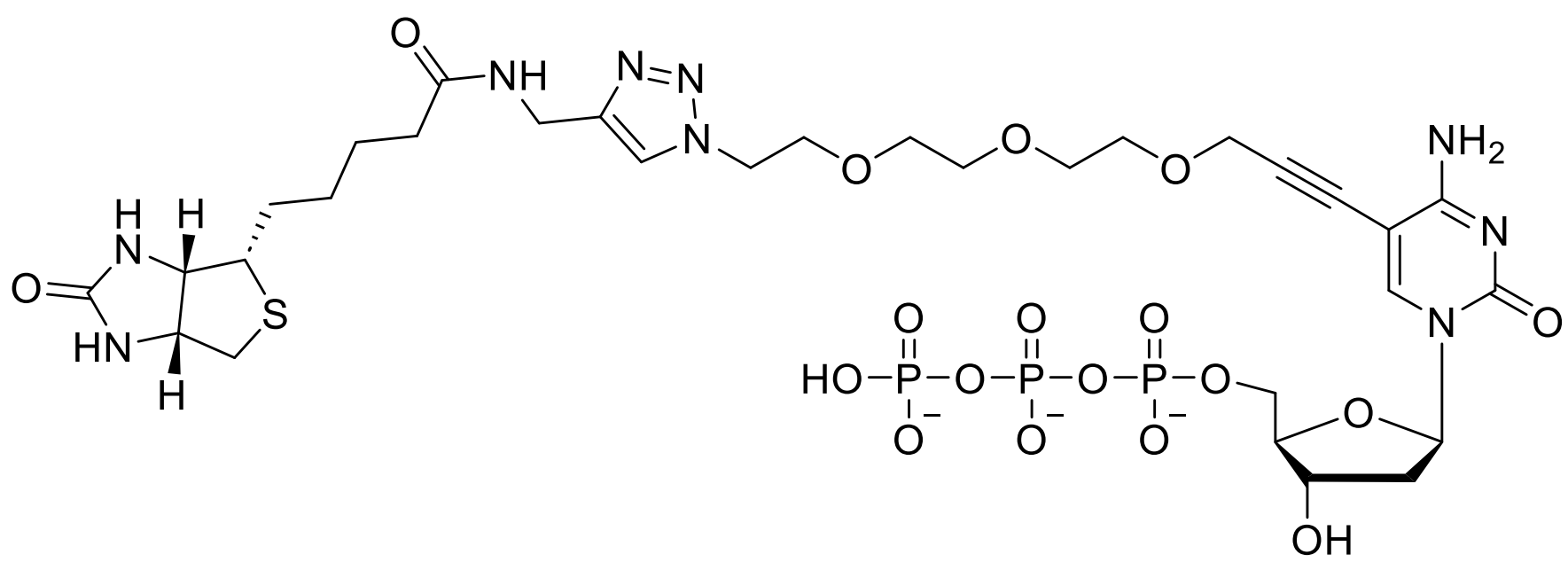

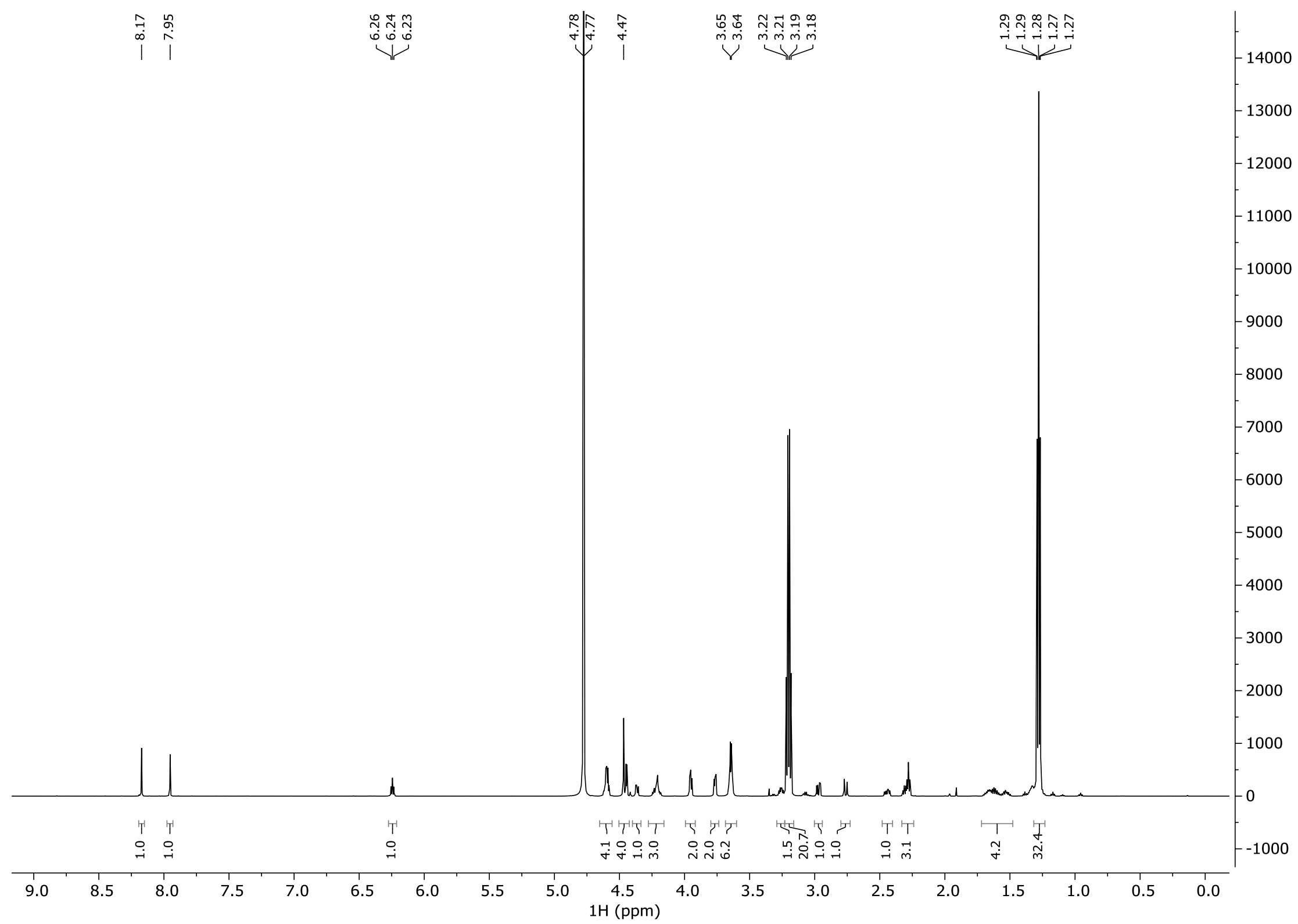

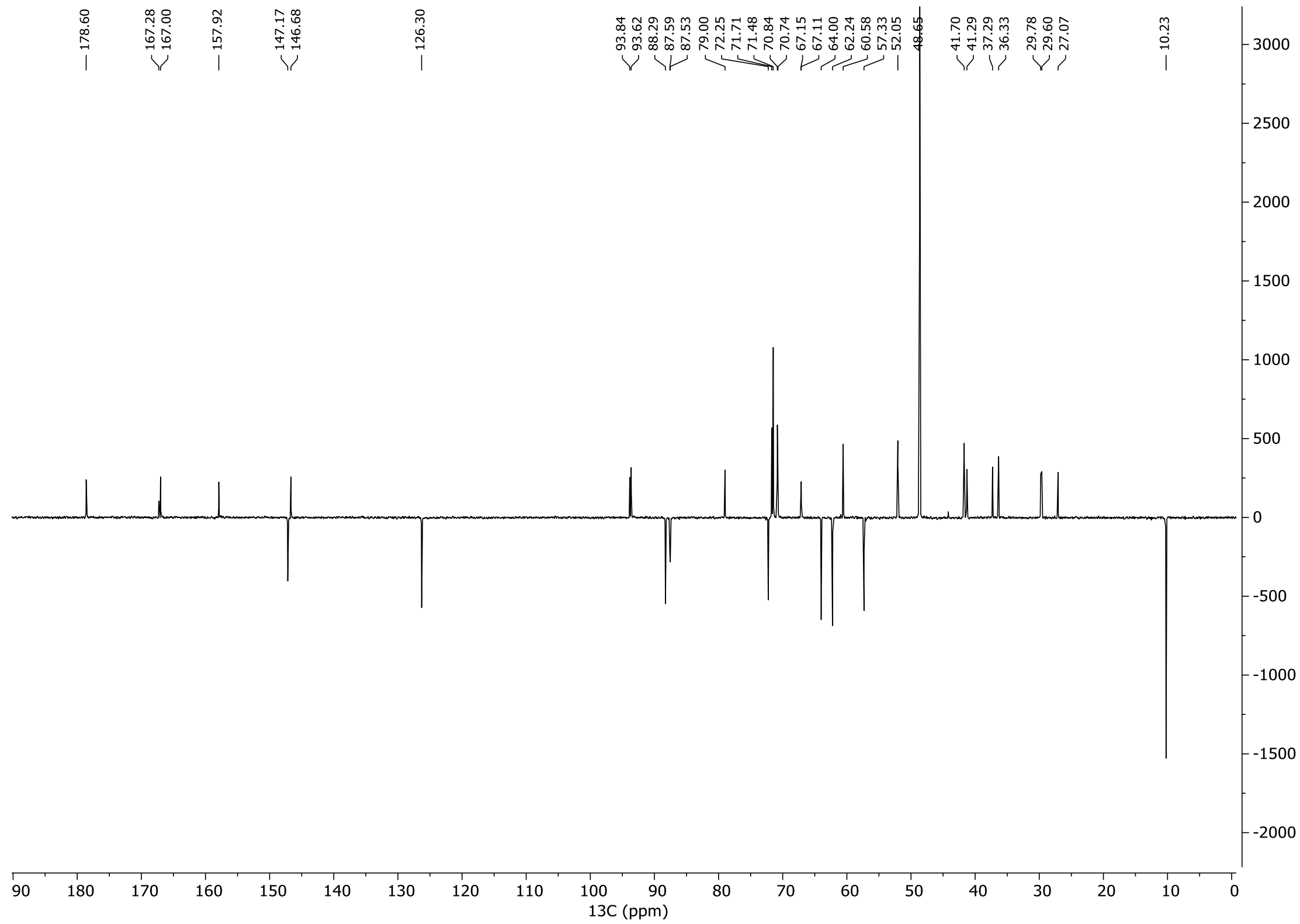

178.60
167.28
167.00
157.92
147.17
146.68
126.30
93.84
93.62
88.29
87.59
87.53
79.00
72.25
71.71
71.48
70.84
70.74
67.15
67.11
64.00
62.24
60.58
57.33
52.05
48.65
41.70
41.29
37.29
36.33
29.78
29.60
27.07
10.23
13C (ppm)

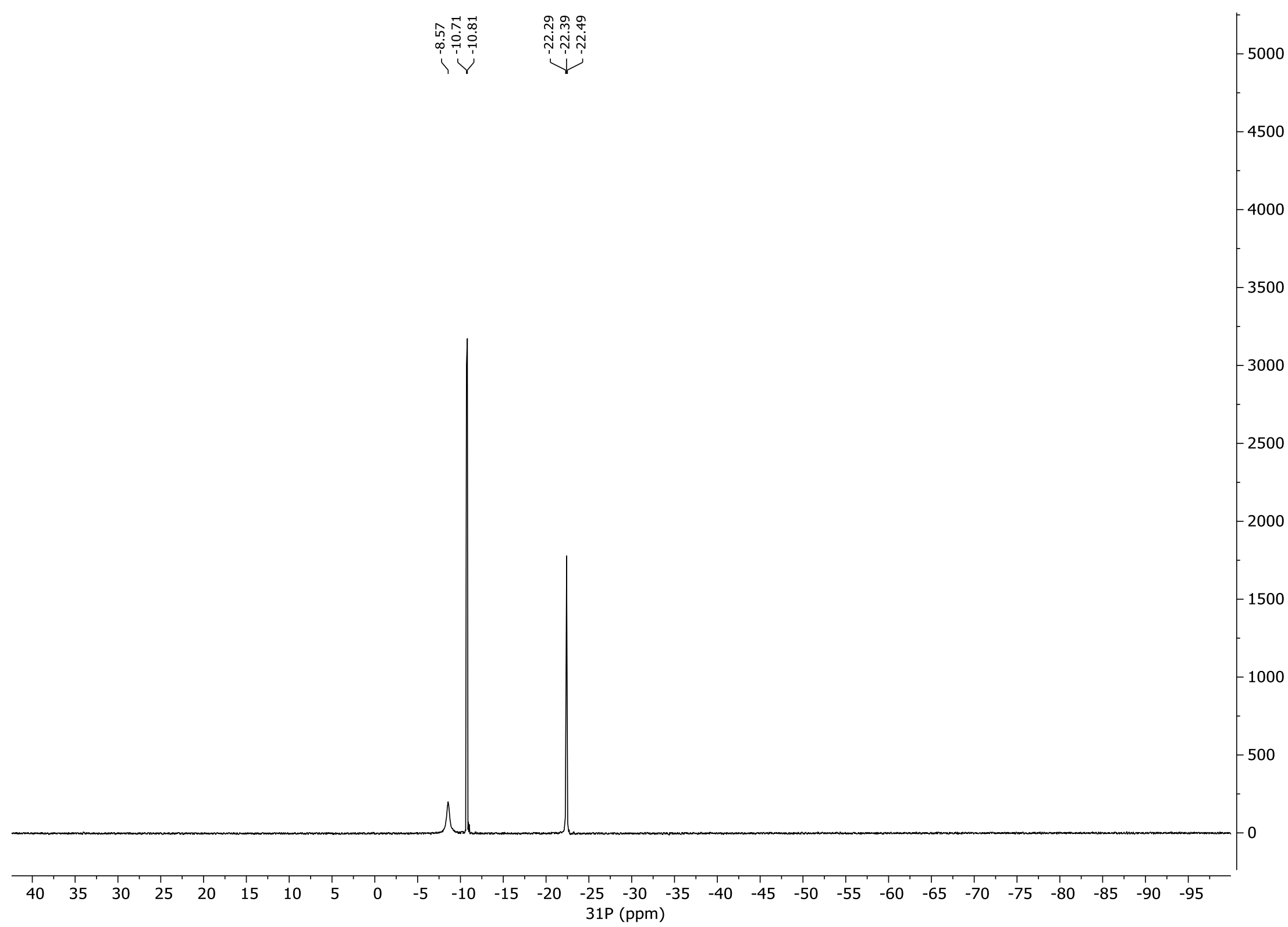

-8.57
-10.71
-10.81
-22.29
-22.39
-22.49
31P (ppm)

5. References

[1] P. Čapek, H. Cahová, R. Pohl, M. Hocek, C. Gloeckner, A. Marx, "An Efficient Method for the Construction of Functionalized DNA Bearing Amino Acid Groups through Cross-Coupling Reactions of Nucleoside Triphosphates Followed by Primer Extension or PCR" *Chemistry - A European Journal* **13**, (2007): 6196. https://doi.org/10.1002/chem.200700220.

[2] V. Percec, P. Leowanawat, H.-J. Sun, O. Kulikov, C. D. Nusbaum, T. M. Tran, A. Bertin, D. A. Wilson, M. Peterca, S. Zhang, N. P. Kamat, K. Vargo, D. Moock, E. D. Johnston, D. A. Hammer, D. J. Pochan, Y. Chen, Y. M. Chabre, T. C. Shiao, M. Bergeron-Brlek, S. André, R. Roy, H.- J. Gabius, P. A. Heiney, "Modular Synthesis of Amphiphilic Janus Glycodendrimers and Their Self-Assembly into Glycodendrimersomes and Other Complex Architectures with Bioactivity to Biomedically Relevant Lectins" *Journal of the American Chemical Society* **135**, (2013): 9055. https://doi.org/10.1021/ja403323y.

[3] P.-C. Lin, S.-H. Ueng, S.-C. Yu, M.-D. Jan, A. K. Adak, C.-C. Yu, C.-C. Lin, "Surface Modification of Magnetic Nanoparticle via Cu(I)-Catalyzed Alkyne-azide [2 + 3] Cycloaddition" *Organic Letters* **9**, (2007): 2131. https://doi.org/10.1021/ol070588f.

[4] A. S. K. Hashmi, T. Häffner, W. Yang, S. Pankajakshan, S. Schäfer, L. Schultes, F. Rominger, W. Frey, "Gold Catalysis: Non-Spirocyclic Intermediates in the Conversion of Furanynes by the Formal Insertion of an Alkyne into an Aryl–Alkyl C-C Single Bond" *Chemistry - A European Journal* **18**, (2012): 10480. https://doi.org/10.1002/chem.201200306.

[5] N. K. Garg, C. C. Woodroofe, C. J. Lacenere, S. R. Quake, B. M. Stoltz, "A ligand-free solid-supported system for Sonogashira couplings: applications in nucleoside chemistry" *Chemical Communications*, (2005): 4551. https://doi.org/10.1039/B505737J.

[6] S. Jäger, G. Rasched, H. Kornreich-Leshem, M. Engeser, O. Thum, M. Famulok, "A Versatile Toolbox for Variable DNA Functionalization at High Density" *Journal of the American Chemical Society* **127**, (2005): 15071. https://doi.org/10.1021/ja051725b.

[7] Y. Lu, S. Song, C. Hou, S. Pang, X. Li, X. Wu, C. Shao, Y. Pei, Z. Pei, "Facile fabrication of branched-chain carbohydrate chips for studying carbohydrate-protein interactions by QCM biosensor" *Chinese Chemical Letters* **1**, (2018): 65. https://doi.org/10.1016/j.cclet.2017.08.003.

[8] M. Kuba, P. Khoroshyy, M. Lepšík, E. Kužmová, D. Kodr, T. Kraus, M. Hocek, "Real-time Imaging of Nascent DNA in Live Cells by Monitoring the Fluorescence Lifetime of DNA-Incorporated Thiazole Orange-Modified Nucleotides" *Angewandte Chemie International Edition* **62**, (2023): e202307548. https://doi.org/10.1002/anie.202307548.

[9] M. T. Marty, A. J. Baldwin, E. G. Marklund, G. K. A. Hochberg, J. L. P. Benesch, C. V. Robinson, "Bayesian Deconvolution of Mass and Ion Mobility Spectra: From Binary Interactions to Polydisperse Ensembles" *Analytical Chemistry* **87**, (2015): 4370. https://doi.org/10.1021/acs.analchem.5b00140.

[10] J. Schindelin, I. Arganda-Carreras, E. Frise, V. Kaynig, M. Longair, T. Pietzsch, S. Preibisch, C. Rueden, S. Saalfeld, B. Schmid, J.-Y. Tinevez, D. J. White, V. Hartenstein, K. Eliceiri, P. Tomancak, A. Cardona, "Fiji: an open-source platform for biological-image analysis" *Nature Methods* **9**, (2012): 676. https://doi.org/10.1038/nmeth.2019.
